# How About Vanadium‐Based Compounds as Cathode Materials for Aqueous Zinc Ion Batteries?

**DOI:** 10.1002/advs.202206907

**Published:** 2023-01-22

**Authors:** Tingting Lv, Yi Peng, Guangxun Zhang, Shu Jiang, Zilin Yang, Shengyang Yang, Huan Pang

**Affiliations:** ^1^ Interdisciplinary Materials Research Center, Institute for Advanced Study Chengdu University Chengdu Sichuan 610106 P. R. China; ^2^ School of Chemistry and Chemical Engineering Yangzhou University Yangzhou Jiangsu 225009 P. R. China

**Keywords:** aqueous zinc‐ion batteries, cathode materials, vanadium‐based compounds

## Abstract

Aqueous zinc‐ion batteries (AZIBs) stand out among many monovalent/multivalent metal‐ion batteries as promising new energy storage devices because of their good safety, low cost, and environmental friendliness. Nevertheless, there are still many great challenges to exploring new‐type cathode materials that are suitable for Zn^2+^ intercalation. Vanadium‐based compounds with various structures, large layer spacing, and different oxidation states are considered suitable cathode candidates for AZIBs. Herein, the research advances in vanadium‐based compounds in recent years are systematically reviewed. The preparation methods, crystal structures, electrochemical performances, and energy storage mechanisms of vanadium‐based compounds (e.g., vanadium phosphates, vanadium oxides, vanadates, vanadium sulfides, and vanadium nitrides) are mainly introduced. Finally, the limitations and development prospects of vanadium‐based compounds are pointed out. Vanadium‐based compounds as cathode materials for AZIBs are hoped to flourish in the coming years and attract more and more researchers' attention.

## Graphical abstract

The research advances in vanadium‐based compounds in recent years are systematically reviewed. The preparation methods, crystal structures, electrochemical performances, and energy storage mechanisms of vanadium‐based compounds are mainly introduced. Finally, the limitations and development prospects of vanadium‐based compounds are pointed out.

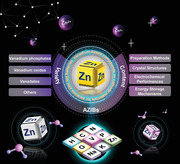

## 1 Introduction

**Scheme 1:**
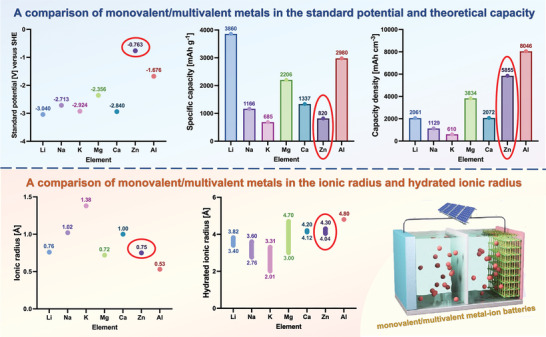
The comparisons of monovalent/multivalent metals in the standard potential and theoretical capacity (Top), ionic radius, and hydrated ionic radius (Bottom).

With the continuous consumption of fossil fuels and the gradual intensification of the greenhouse effect, energy shortages and environmental pollution have become two major problems facing the sustainable development of human society.^[^ ^1^, ^2^, ^3^, ^4^, ^5^, ^6^, ^7^ ^]^ The development of clean and green energy resources, such as wind, solar, and tidal power, has received widespread attention.^[^ ^8^, ^9^, ^10^, ^11^, ^12^ ^]^ Therefore, it's essential for efficient energy storage and conversion devices.^[^ ^13^, ^14^, ^15^, ^16^ ^]^ In recent years, lithium‐ion batteries (LIBs) have gradually occupied the market for commercial rechargeable batteries due to their high energy density, long service life, and mature preparation technology.^[^ ^17^, ^18^, ^19^ ^]^ However, despite the commercial success, the limited lithium resources, high cost, unsafety, and environmental pollution problems caused by the flammable and toxic organic electrolyte have seriously hindered the further development and large‐scale applications of LIBs.^[^ ^20^, ^21^, ^22^ ^]^ Compared with organic electrolytes, which have a large number of safety risks, aqueous electrolytes (AEs) have many technical advantages, such as higher safety, low price, and facile manufacturing, and are expected to be employed to develop the next generation of green rechargeable batteries.^[^ ^23^, ^24^, ^25^, ^26^ ^]^ More importantly, the ionic conductivity of AEs is two orders of magnitude higher than that of organic electrolytes, which can promote power densities and fast charging capability.^[^ ^27^, ^28^, ^29^, ^30^, ^31^ ^]^ Since 1994, Li et al.^[^ ^32^ ^]^ introduced aqueous solution as an electrolyte for LIBs. Nowadays, different kinds of aqueous rechargeable batteries on the basis of charge carriers with various valence states (e.g., Li^+^, Na^+^, K^+^, Mg^2+^, Zn^2+^, Ca^2+^, and Al^3+^) have been developed.^[^ ^33^, ^34^, ^35^, ^36^ ^]^ Among these aqueous rechargeable monovalent/multivalent metal‐ion batteries, aqueous zinc‐ion batteries (AZIBs) are one of the most concerned devices due to their high capacity of 820 mAh g^−1^, excellent volumetric capacity of 5855 mAh cm^−3^, low redox potential of −0.763 V (vs standard hydrogen electrode, SHE), large hydrogen evolution overpotential in AE, repeatable stripping/plating, as well as abundant and cheap (**Scheme** **1**).^[^ ^37^, ^38^, ^39^, ^40^, ^41^ ^]^

**Scheme 2:**
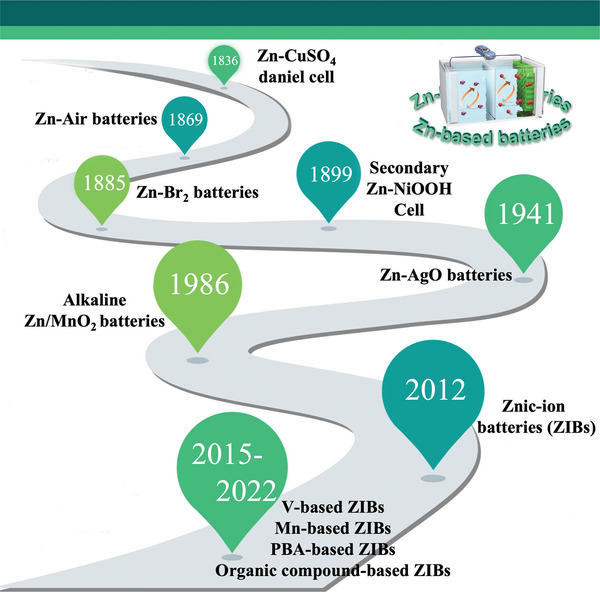
Brief history of Zn‐based batteries.

Before AZIBs flourished as they do today, zinc‐based batteries had a long history of development (**Scheme** **2**). Zinc‐manganese batteries have dominated the primary battery market since their invention by French engineer Georges Le Klain in 1868.^[^ ^42^ ^]^ Alkaline Zn/MnO_2_ batteries were successfully developed on the basis of zinc‐manganese batteries in 1986.^[^ ^43^ ^]^ However, the early Zn/MnO_2_ batteries were relatively backward in technology, costly, and wasteful of resources. To save resources and enhance efficiency, rechargeable alkaline Zn/MnO_2_ batteries were developed. However, rechargeable alkaline Zn/MnO_2_ batteries still have some disadvantages, such as low CE and fast capacity decay due to the irreversible reactions on Zn/MnO_2_ batteries’ electrodes.^[^ ^44^ ^]^ Until 2012, Xu and co‐workers^[^ ^45^ ^]^ successfully developed rechargeable AZIBs using mild zinc sulfate as the electrolyte, *α*‐MnO_2_ as the positive material, and Zn as the negative material. Polycrystalline MnO_2_ (mainly including *α*, *β*, R, *δ*, *T*, *λ*, *ε*, and *γ*), Prussian blue, and its analogs (e.g., copper hexacyanoferrate and zinc hexacyanoferrate) have attracted much attention as cathode materials.^[^ ^46^, ^47^, ^48^, ^49^, ^50^, ^51^ ^]^ However, the crystal structure of MnO_2_ is unstable, and it shows poor cycling performance in the (dis)charging process.^[^ ^52^, ^53^ ^]^ In addition, Prussian blue and its analogs have a stable crystal structure, but poor Zn storage capacity (less than 100 mAh g^−1^).^[^ ^54^, ^55^ ^]^ Therefore, the development of structurally stable and high‐capacity cathode materials has become one of the keys for the research of AZIBs.^[^ ^56^, ^57^, ^58^ ^]^

**Scheme 3:**
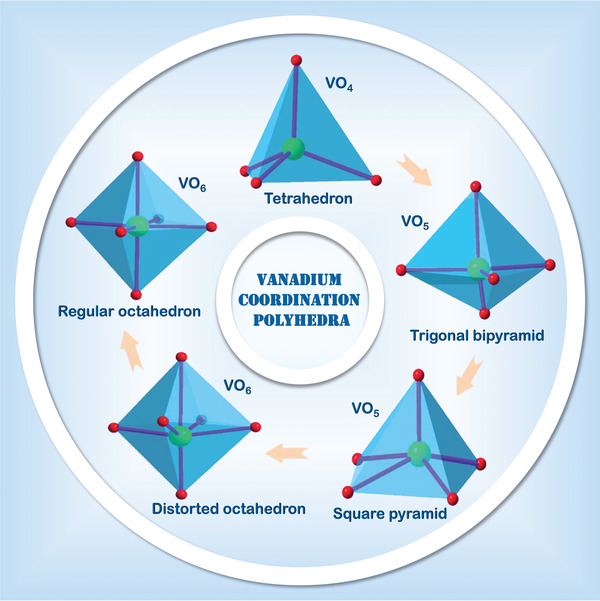
Various vanadium coordination polyhedrons (Red O atoms, green V atoms, and pink V—O bonds).

In 2016, Dipan Kundu and co‐workers^[^ ^59^ ^]^ used layered Zn_0.25_V_2_O_5_ *n*H_2_O as a cathode material to store Zn^2+^. The specific capacity can reach 300 mAh g^−1^ and the capacity retention rate was still more than 80% after the cycle life exceeded 1000 times. After that, due to the low cost and multiple oxidation states of vanadium, different kinds of vanadium‑based compounds (e.g., vanadium phosphates, vanadium oxides, vanadates, vanadium sulfides, and vanadium nitrides (VNs), etc.) were constructed to store Zn^2+^.^[^ ^60^, ^61^, ^62^, ^63^, ^64^, ^65^ ^]^ Vanadium‐based compounds have higher reversible capacities, better reactivity, and longer cycle lives than MnO_2_ and Prussian blue and its analogs.^[^ ^66^, ^67^, ^68^, ^69^ ^]^ In addition, they also have diverse crystal structures, including layered, tunnel, NASICON structure, and so on, which can not only achieve multi‐electron transfer, but also help to achieve local electric neutrality and alleviate the polarization problem caused by Zn^2+^ intercalation.^[^ ^70^ ^]^ The multiple oxidation states of vanadium (ranging from +2 to +5) and the deformation of V—O polyhedral lead to a variety of different compositions and structural frameworks of vanadium‐based compounds constructed by various coordination polyhedral, for instance, VO_4_ tetrahedron, VO_5_ trigonal bipyramid/square pyramid and VO_6_ distorted/regular octahedron (**Scheme** **3**).^[^ ^71^ ^]^ Until now, several types of vanadium‑based compounds have been studied as AZIBs cathodes and have shown superior Zn^2+^ storage capacity.^[^ ^72^, ^73^, ^74^, ^75^ ^]^ However, there are still some key issues for vanadium‐based compounds such as complex energy storage mechanisms, low average operating voltage, and unsatisfactory performance. Furthermore, the open framework of vanadium‐based compounds is prone to collapse, resulting in the dissolution of vanadium after long cycles.^[^ ^76^, ^77^ ^]^

**Scheme 4:**
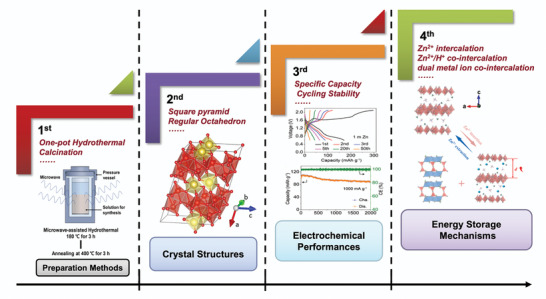
Graphical abstract of the introduction of vanadium‐based compounds in this review.

In this review, we will introduce different vanadium‐based compounds and their applications in AZIBs, such as the preparation methods, crystal structures, electrochemical performances, and energy storage mechanisms (**Scheme** **4**). In the end, further development and prospects of vanadium‐based AZIBs cathodes are introduced.

## 2 Vanadium Phosphates

As a special vanadium‑based compound, vanadium phosphate can enhance the redox potential of vanadium‑based compounds through the induction of polyanion PO_4_^3−^.^[^ ^78^ ^]^ In addition, the strong polarization of O^2−^ will reduce the M—O covalent bonds and further enhance the transition metal redox potential.^[^ ^79^ ^]^ There are two types of vanadium‑based compounds: 1) The layered vanadyl phosphates with large open 2D channel, which provide a wide channel for Zn^2+^ (de)intercalation processes (e.g., layered VOPO_4_, VO(OH)_2_PO_4_).^[^ ^80^ ^]^ 2) Vanadium phosphates with high structural stability, interconnected 3D channels, and flexible active sites that can be constructed internally, which are conducive to the transport of Zn^2+^ (e.g., polyanionic‐type and NASICON‐type vanadium phosphates).^[^ ^81^ ^]^ The electrochemical properties of some common vanadium phosphates as cathode materials are shown in **Table** **1**.

**Table 1:** Electrochemical performance of vanadium phosphates as cathodes materials in AZIBs

Materials	Electrolyte	Specific capacity [mAh g^−1^] (current density [A g^−1^])	Capacity retention (cycles numbers)	Voltage range [V]	Ref.
VOPO_4_	21 m LiTFSI + 1 m Zn(Tr)_2_	139 (0.05)	93% (1000)	0.8–2.1	[82]
VOPO_4_·*x*H_2_O	13 m ZnCl_2_ + 0.8 m H_3_PO_4_	170 (0.1)	≈96% (500)	0.7–1.9	[87]
bilayer‐VOP	2 m ZnSO_4_	313.6 (0.1)	76.8% (2000)	0.2–1.9	[86]
KVOPO_4_	4 m Zn(CF_3_SO_3_)_2_	68 (0.5)	≈100% (400)	0.8–2.0	[90]
Zn_0.4_VOPO_4_	2 m Zn(CF_3_SO_3_)_2_	161.4 (0.1)	71.4% (1000)	0.2–1.9	[91]
PPy‐VOPO4	1 m Zn(CF_3_SO_3_)_2_ + Acetonitrile electrolyte with 10 vol% water	86 (0.025)	90.6% (350)	0.5–2.0	[93]
PA‐VOP	2 m Zn(CF_3_SO_3_)_2_	268.2 (0.1)	92.3% (2000)	0.2–1.9	[92]
Li_3_V_2_(PO_4_)_3_	1 m Zn(ClO_4_)_2_ + Acetonitrile electrolyte with 10.85 vol% water	128 (1 C)	88.7% (1000)	0.7–2.2	[78]
Li_3_V_2_(PO_4_)_3_	1 m Zn(OTf)_2_ + 15 m LiTFSI	126.7 (0.2)	82.3% (2000)	0.6–2.1	[108]
Li_3_V_2_(PO_4_)_3_	1 m Li_2_SO_4_ + 2 m ZnSO_4_	128 (0.2 C)	85.4% (200)	0.7–2.1	[107]
Li_3_V_2_(PO_4_)_3_	2 m Zn(OTf)_2_ with 70 wt.% Polyethylene glycol 400	≈80 (0.5 C)	83.5% (100)	0.2–1.9	[109]
Li_3_V_2_(PO_4_)_3_	1 m Zn(CF_3_SO_3_)_2_ + 20 m LiTFSI	111 (0.2)	93.6% (100)	0.7–1.7	[134]
Li_3_V_2_(PO_4_)_3_@C	2 m ZnSO_4_ + 1 m Li_2_SO_4_	95 (2 C)	91% (50)	0.7–1.7	[81]
Na_3_V_2_(PO_4_)_3_	2 m ZnSO_4_ + 1 m Li_2_SO_4_	96 (0.2 C)	84.1% (200)	0.7–2.1	[107]
Na_3_V_2_(PO_4_)_3_	Zn(CF_3_SO_3_)_2_ in triethyl phosphate	74 (0.5)	≈100% (600)	0.6–1.8	[113]
Na_3_V_2_(PO_4_)_3_	0.5 m Zn(CH_3_CO_2_)_2_	90 (0.1)	80% (100)	0.6–1.8	[135]
Na_3_V_2_(PO_4_)_3_	2 m ZnOTF_2_ + 1 m NaOTF with polyethylene glycol	90 (0.05)	66.7% (300)	0.6–1.8	[136]
Na_3_V_2_(PO_4_)_3_/C	0.5 m Zn(CH_3_OO)_2_	97 (0.5 C)	74% (100)	0.8–1.7	[102]
Na_3_V_2_(PO_4_)_3_/C	0.5 MCH_3_OONa + Zn(CH_3_OO)_2_ solution	92 (0.05)	77% (200)	0.5–1.7	[112]
Na_3_V_2_(PO_4_)_3_/rGO	2 m Zn(CF_3_SO_3_)_2_	114 (0.05)	75% (200)	0.6–1.8	[114]
Sr‐doped Na_3_V_2_(PO_4_)_3_	Hydrated gel electrolyte	108 (0.5 C)	96.2% (400)	(−0.13)–2.6	[115]
Na_3_V_2_(PO_4_)_3_O_1.6_F_1.4_	25 m ZnCl_2_ + 5 m NH_4_Cl	155 (0.05)	73.5% (7000)	0.2–1.4	[128]

### 2.1 Vanadyl Phosphates

#### 2.1.1 VOPO_4_

**Figure 1:**
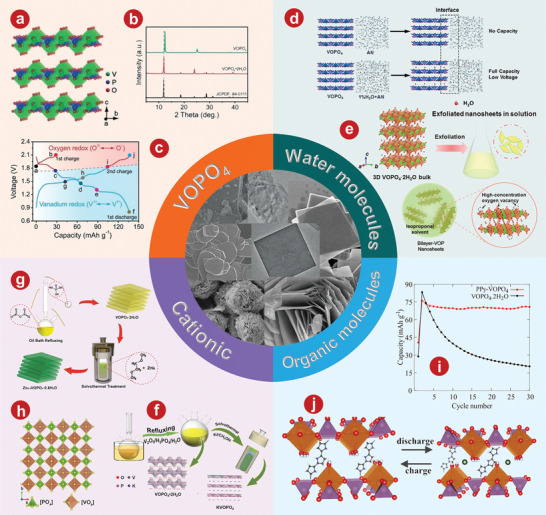
a) Crystal structure of VOPO_4_. b) XRD patterns of VOPO_4_ 2H_2_O and VOPO_4_. c) Charge/discharge curves of Zn/VOPO_4_ batteries at 0.05 A g^−1^. d) Schematic illustrations of VOPO_4_ in 0.1m Zn(OTf)_2_‐AN without water (top) and with 1% H_2_O (bottom). e) Schematic of liquid‐phase exfoliation and predictable oxygen vacancy formation in bilayer‐structured VOPO_4_ 2H_2_O nanosheets. f) Schematic illustration for the preparation process of KVOPO_4_. g) Schematic illustration of the Zn^2+^ incorporation in layered VOPO_4_ 2H_2_O by an appropriate solvothermal process. h) SXRD pattern of the Zn_0.4_VOPO_4_·0.8H_2_O single crystal along the *b*‐axis. i) Comparison of the cycling stabilities for VOPO_4_ 2H_2_O and PPy‐VOPO_4_ at a current rate of 30 mA g^−1^ in 1 m Zn(CF_3_SO_3_)_2_/acetonitrile electrolyte with 10 vol % water. j) Schematic depicting overall mechanism of zinc intercalation or deintercalation processes into the PPy‐VOPO_4_ host structure. Green spheres indicate Zn^2+^ ions. a–c) Reproduced with permission.^[^ ^82^ ^]^ Copyright 2019, Wiley‐VCH. d) Reproduced with permission.^[^ ^85^ ^]^ Copyright 2018, Wiley‐VCH. e) Reproduced with permission.^[^ ^86^ ^]^ Copyright 2021, Wiley‐VCH. f) Reproduced with permission.^[^ ^86^ ^]^ Copyright 2021, Elsevier. g, h) Reproduced with permission.^[^ ^87^ ^]^ Copyright 2020, American Chemical Society. i, j) Reproduced with permission.^[^ ^93^ ^]^ Copyright 2019, American Chemical Society.

Layered VOPO_4_, with typical polyanionic cathodes, has corresponding 2D diffusion channels. Wan et al.^[^ ^82^ ^]^ have developed a highly reversible AZIBs based on layered VOPO_4_ cathode and water‐electrolyte in salt. The VOPO_4_ consists of corner‐sharing VO_6_ octahedron connected to the PO_4_ tetrahedron (**Figure** **1**a).^[^ ^83^ ^]^ Different from VOPO_4_ 2H_2_O, the X‐ray diffraction (XRD) pattern of VOPO_4_ showed only (*00l*) base plane lines and no other (*h0l*) lines (Figure 1b). Zn/VOPO_4_ batteries can be charged directly from the open circuit voltage (point *a*) to 2.1 V (point *b*), from which an obvious platform was observed (Figure 1c).

#### 2.1.2 The Intercalation of H_2_O

##### VOPO_4_ 2H_2_O

In addition to VOPO_4_, the most recent progress reveals layered VOPO_4_ 2H_2_O can also be considered as a promising cathode candidate for AZIBs owing to its higher discharge platform from the induction effect.^[^ ^38^ ^]^ The crystal structure of VOPO_4_ 2H_2_O was revealed by Tietze in 1981.^[^ ^84^ ^]^ The structure is described as a superposition of VO(OH)_2_PO_4_ layers along the c‐axis, with water molecules occupying a large layer spacing. In 2018, Wang et al.^[^ ^85^ ^]^ revealed the storage mechanism of layered VOPO_4_ 2H_2_O framework for zinc ions and studied the influence of moisture content on the diffusion ability of Zn^2+^ in AZIBs system, as well as developing the application of layered VOPO_4_ 2H_2_O in ZIBs cathode for the first time. As shown in Figure 1d, H_2_O migrates from AE into the VOPO_4_ lattice, and simultaneously creates a “wet interface” that assists the Zn^2+^ intercalation, while the dry interface accepts the Zn^2+^ with significant resistance (see the dotted box). Wu and colleagues^[^ ^86^ ^]^ developed a liquid stripping strategy in isopropanol that can introduce a mass of oxygen permeates with high concentration to produce oxygen‐rich vacancy bilayer nanosheets with high yield (Figure 1e). The specific capacity of the bilayer‐structured VOPO_4_ 2H_2_O cathode is 313.6 mAh g^−1^ at 0.1 A g^−1^.

##### VOPO_4_ *x*H_2_O

Layered VOPO_4_ *x*H_2_O, which can be obtained by cryogenic synthesis procedures, provides adjustable interlaminar spacing to accommodate inserted cations. Shi et al.^[^ ^87^ ^]^ prevented the decomposition and dissolution of VOPO_4_ *x*H_2_O by the electrolyte containing a high concentration of inexpensive and highly soluble ZnCl_2_ salt. This H^+^ and Zn^2+^ sequential intercalation structure provides AZIBs with a high capacity of 170 mAh g^−1^.

Recent studies have shown that the capacity and cycling performance of the cathode can be significantly enhanced by cationic intercalation and organic molecule intercalation to adjust the interlayer structure.^[^ ^88^, ^89^ ^]^

#### 2.1.3 The Intercalation of K^+^

##### KVOPO_4_ 2H_2_O

Zhu et al.^[^ ^90^ ^]^ used VOPO_4_ 2H_2_O as a precursor to intercalate K^+^ into the VOPO_4_ layers through ion exchange (Figure 1f). At a current density of 500 mA g^−1^, KVOPO_4_ electrode of AZIBs can achieve over 400 cycles, which shows a superb cycling stability.

#### 2.1.4 The Intercalation of Zn^2+^ and H_2_O

##### Zn_0.4_VOPO_4_ 0.8H_2_O

Cations can partially replace the structural water in VOPO_4_ 2H_2_O or can be inserted directly into VOPO_4_. Wu and colleagues^[^ ^87^ ^]^ developed a layered phosphate, Zn_0.4_VOPO_4_ 0.8H_2_O, by introducing Zn^2+^ into the VOPO_4_ 2H_2_O framework (Figure 1g). There is no change in the interlayer VOPO_4_ framework compared to VOPO_4_ 2H_2_O (Figure 1h). At the power density of 136.2 W kg^−1^, the energy density of Zn// Zn_0.4_VOPO_4_ 0.8H_2_O battery reaches up to 219.8 Wh kg^−1^.

#### 2.1.5 The Intercalation of Polypyridine in VOPO_4_

Highly conductive organic polymers show the potential for further extending the layer spacing due to the dense charge distribution of intercalated metal ions.^[^ ^92^ ^]^ Vivek Verma and colleagues^[^ ^93^ ^]^ greatly improved the output and long‐term capacity retention of AZIBs by the pre‐intercalation of polypyridine between crystalline layers and using water‐controlled electrolytes. Comparison of cyclic stability between VOPO_4_ 2H_2_O and PPy‐VOPO_4_ was shown in Figure 1i. Obviously, PPy‐VOPO_4_ had better capacity retention. As shown in Figure 1j, the mechanisms can be inferred through the study of structural characterization.

### 2.2 NASICON‐Type Phosphates

NASICON‐type phosphates (M_3_V_2_(PO_4_)_3_ (M = Li, Na, K)) with sodium superionic conductor structure have a highly covalent 3D host framework with rich clearance space, allowing efficient diffusion of ions.^[^ ^94^, ^95^, ^96^, ^97^, ^98^ ^]^ In recent years, the NASICON‐type phosphate structure has been used as cathode materials to store monovalent metal ions, such as Li^+^, Na^+^, and K^+^.^[^ ^99^, ^100^, ^101^ ^]^ Relevant studies show that the radius of Na^+^ (0.98 Å) is larger than that of Zn^2+^ (0.74 Å), indicating that Zn^2+^ has great potential in the framework of NASICON‐type phosphates.^[^ ^102^ ^]^ In addition, the NASICON‐type phosphate structure has a higher energy density and redox potential than the homologous vanadium oxides because of the strong inducible effect of PO_4_^3−^polyanion and the strong P—O bond.^[^ ^87^ ^]^

#### 2.2.1 Li_3_V_2_(PO_4_)_3_

**Figure 2:**
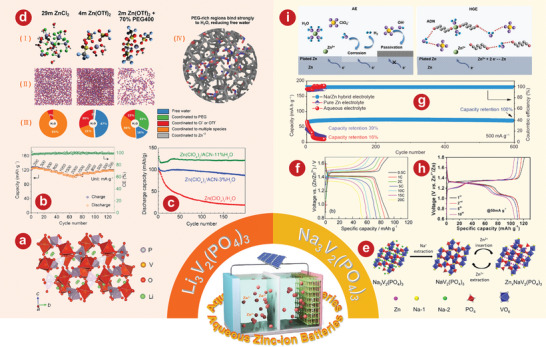
a) Schematic illustration of the Li_3_V_2_(PO_4_)_3_ crystal structure. b) Rate performance of the Li_3_V_2_(PO_4_)_3_ cathode in 1 mol kg^−1^ Zn + 15 mol kg^−1^ Li electrolyte. c) Cycling stability of the Li_3_V_2_(PO_4_)_3_ electrode at the 200th cycle in Zn cells with different electrolytes at 2C. d) Electrolyte structure in 29 mol kg^−1^ ZnCl_2_, 4 mol kg^−1^ Zn(OTf)_2_, and 70PEG electrolytes. (I) Typical Zn^2+^ solvation structures. (II) Simulation snapshots showing only the water molecules. (III) Distribution of water populations. (IV) Water molecules absorbed into the PEG‐rich region of the 70PEG electrolyte. e) Schematic representation for phase transition of Na_3_V_2_(PO4)_3_ cathode during cycling. f) Charge‐discharge curves of the system at different C rates of the Zn/Na_3_V_2_(PO4)_3_ battery. g) The comparison of long‐term cycle performance at a current of 500 mA g^−1^ in different electrolytes. h) Charge/discharge curves of Na_3_V_2_(PO4)_3_@reduced graphene oxide composites at 50 mA g^−1^. i) Schematic illustration of Zn^2+^ solvation structure and corresponding interfacial reactions in AE and HGE. a, b) Reproduced with permission.^[^ ^108^ ^]^ Copyright 2021, The Royal Society of Chemistry. c) Reproduced with permission.^[^ ^78^ ^]^ Copyright 2021, The Royal Society of Chemistry. d) Reproduced with permission.^[^ ^109^ ^]^ Copyright 2022, American Chemical Society. e) Reproduced with permission.^[^ ^102^ ^]^ Copyright 2016, Elsevier. f) Reproduced with permission.^[^ ^112^ ^]^ Copyright 2016, Elsevier. g) Reproduced with permission.^[^ ^113^ ^]^ Copyright 2021, Springer. h) Reproduced with permission.^[^ ^114^ ^]^ Copyright 2019, Wiley‐VCH. i) Reproduced with permission.^[^ ^115^ ^]^ Copyright 2021, Wiley‐VCH.

Li_3_V_2_(PO_4_)_3_ has two kinds of framework structure distinguished by the connection of “lantern” element [V_2_(PO_4_)_3_], namely rhomboid phase and monoclinic phase.^[^ ^103^, ^104^, ^105^ ^]^ Structural differences result in their various electrochemical performances. However, the three mobile Li^+^ in the monoclinic phase make electrochemical performance of monoclinic phase better than that of the rhomboid phase, which leads to greater research value.^[^ ^106^ ^]^ In 2016, Zhao et al.^[^ ^107^ ^]^ demonstrated the feasibility of Li_3_V_2_(PO_4_)_3_ for AZIBs, which aroused great interest among researchers. Li and colleagues^[^ ^108^ ^]^ demonstrated that the capacity decay and voltage drop problems of the Li_3_V_2_(PO_4_)_3_ cathode were significantly solved when using a concentrated AE based on zinc and lithium salts. **Figure** **2**a illustrates the crystal structure of Li_3_V_2_(PO_4_)_3_. The rate performance of Li_3_V_2_(PO_4_)_3_ indicates that a high capacity of 100.5 mA h g^−1^ is achieved at 2000 mA g^−1^ (Figure 2b). Li et al.^[^ ^78^ ^]^ took advantage of the optimal solvent combination of water and acetonitrile in the electrolyte, which can effectively prevent the dissolution or decomposition of Li_3_V_2_(PO_4_)_3_ into vanadium oxide without sacrificing the disinsertion and insertion kinetics of Zn^2+^. To enhance the conductivity, rGO was added to Li_3_V_2_(PO_4_)_3_. In Zn(ClO_4_)_2_/acetonitrile ≈11% H_2_O electrolytes, its performance is further improved. The initial capacity of the electrode reaches 125 mA h g^−1^ and remains at 121 mA h g^−1^ after 200 cycles. In addition, Li and coworkers^[^ ^109^ ^]^ focused on the inhibition of harmful H^+^ intercalation by adjusting the solvation structure with Li_3_V_2_(PO_4_)_3_ as the model cathode. Electrolyte structure in 29 mol kg^−1^ ZnCl_2_, 4 mol kg^−1^ Zn(OTf)_2_, and 70PEG electrolytes is shown in Figure 2d. The novel PEG hybrid electrolyte not only has a good inhibition of H^+^ intercalation, but also has high reversible plating/stripping performance, with a CE value of 99.7% after 150 cycles.

#### 2.2.2 Na_3_V_2_(PO_4_)_3_

Different from Li_3_V_2_(PO_4_)_3_, the basic frame structure of Na_3_V_2_(PO_4_)_3_ with rhomboid phase is VO_6_ octahedron and PO_4_ tetrahedron, which share angular connection. There are two kinds of sodium ions in the crystal structure, in which each PO_4_ tetrahedron is connected by two VO_6_ octahedrons and sodium 1, while sodium 2 is located only between the two VO_6_ octahedrons.^[^ ^110^ ^]^ By comparing the length of the sodium‐oxygen bond, it was found that the occupancy rate of sodium 2 was smaller than that of sodium 1, which made the corresponding sodium ion easier to be extracted in the electrochemical process.^[^ ^111^ ^]^ Huang's group.^[^ ^102^ ^]^ first developed Na_3_V_2_(PO_4_)_3_ with NASICON structure as the cathode material of AZIBs. Phase transition diagram of Na_3_V_2_(PO_4_)_3_ cathode cycle is shown in Figure 2e. Na_3_V_2_(PO_4_)_3_ has great potential as cathode materials of AZIBs, which show excellent rates and cyclic performances. In addition, Huang's group.^[^ ^112^ ^]^ enhanced the electrochemical properties of Na_3_V_2_(PO_4_)_3_ by co‐incorporating carbon and reduced graphene oxide (rGO). This aqueous hybrid battery, which uses carbon‐rGO‐Na_3_V_2_(PO_4_)_3_ composite as cathode, has a capacity of 92 mAh g^−1^ and a high and flat operating voltage of 1.42 V at a current density of 50 mA g^−1^ (Figure 2f). Li et al.^[^ ^113^ ^]^ systematically studied the capacity degradation mechanism of Na_3_V_2_(PO_4_)_3_ and proposed a new organic double salt electrolyte that achieved good cyclic stability of 600 cycles at 500 mA g^−1^ without a capacity loss (Figure 2g). Hu and his colleagues^[^ ^114^ ^]^ demonstrated that Na_3_V_2_(PO_4_)_3_@rGO microspheres have a simultaneous Zn^2+^/Na^+^ (de)intercalation behavior in a single‐component 2.0 m Zn(CF_3_SO_3_)_2_ electrolyte. As shown in Figure 2h, the average discharge platform of Na_3_V_2_(PO_4_)_3_@rGO microspheres at 50 mA g^−1^ is 1.23 V. Lin et al.^[^ ^115^ ^]^ developed a dual‐function AZIB hydration gel electrolyte with NASICON‐type strontium‐doped Na_3_V_2_(PO_4_)_3_ as the cathode, which can effectively reduce the para‐reaction of water at the cathode. Solvation structure and interfacial reaction diagram of Zn^2+^ in AE and hydrated gel electrolyte (HGE) is shown in Figure 2i. Using the NASICON‐type strontium‐doped Na_3_V_2_(PO_4_)_3_ as the cathode, the AZIBs can achieve more than 8000 cycles at 10 C, and still maintain the high capacity of 90 mAh g^−1^.

#### 2.2.3 K_3_V_2_(PO_4_)_3_

With the successful application of homologous Li_3_V_2_(PO_4_)_3_ and Na_3_V_2_(PO_4_)_3_ cathodes, K_3_V_2_(PO_4_)_3_ has attracted extensive attention.^[^ ^116^, ^117^ ^]^ However, at present, relevant applications are concentrated in LIBs and sodium‐ion batteries, and there is no application in AZIBs with excellent electrochemical performance. K_3_V_2_(PO_4_)_3_ is expected to be widely used in AZIBs in the future.

### 2.3 NASICON‐Type Phosphate Analogs

**Figure 3:**
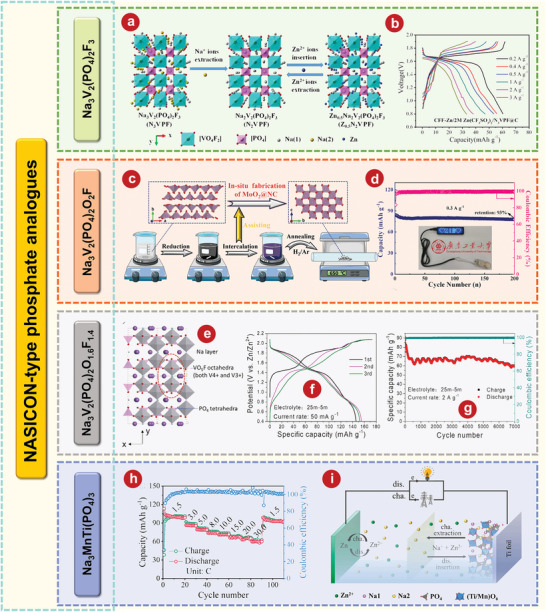
a) Schematic illustration of Zinc storage mechanism. b) Charge/discharge profiles of carbon film functionalizing‐Zn//Na_3_V_2_(PO_4_)_2_F_3_@C battery at various current densities from 0.2 to 3 A g^−1^. c) Illustration of synthesis process of MoO_2_@NC. d) Cycling performance of MoO_2_@NC||Zn‐Na_3_V_2_(PO_4_)_2_O_2_F full cell at 0.3 A g^−1^. e) Schematic of the crystal structure of Na_3_V_2_(PO_4_)_2_F_3_. f) The initial galvanostatic charge/discharge profiles of the Na_3_V_2_(PO_4_)_2_O_1.6_F_1.4_ electrode in the 25 mol kg^−1^ + 5 mol kg^−1^ electrolyte at a current rate of 50 mA g^−1^. g) The cycling performance of Na_3_V_2_(PO_4_)_2_O_1.6_F_1.4_ electrode in the 25 mol kg^−1^ + 5 mol kg^−1^ electrolyte at a current rate of 2 A g^−1^. h) Rate capability of the Zn//Na_3_MnTi(PO_4_)_3_ battery from 1.5 to 30.0 C. i) Schematic illustration of aqueous rechargeable hybrid Zn//Na_3_MnTi(PO_4_)_3_ battery during charge and discharge processes. a, b) Reproduced with permission.^[^ ^124^ ^]^ Copyright 2018, Elsevier. c, d) Reproduced with permission.^[^ ^126^ ^]^ Copyright 2021, Wiley‐VCH. e–g) Reproduced with permission.^[^ ^128^ ^]^ Copyright 2020, Wiley‐VCH. h, i) Reproduced with permission.^[^ ^133^ ^]^ Copyright 2021, Elsevier.

Basic studies on Li_3_V_2_(PO_4_)_3_ and Na_3_V_2_(PO_4_)_3_ have shown that NASICON‐type materials have suitable diffusion channels and guest ion attachment sites and have the storage capacity of Zn^2+^. Na_3_V_2_(PO_4_)_2_F_3_, as an outstanding representative of NASICON‐type phosphate analogs, has the advantages of high energy density, interstitial spaces, and good structural stability.^[^ ^118^, ^119^ ^]^ As the F—V bond is more ionic than the O—V bond, the working potential of Na_3_V_2_(PO_4_)_2_F_3_ is reported to be as high as ≈3.9 V and the energy density as high as ≈500 Wh kg^−1^.^[^ ^120^ ^]^ Na_3_V_2_(PO_4_)_2_F_3_ with *Amam* space group belongs to orthorhombic crystal system, which is composed of [V_2_O_8_F_3_] bi‐octahedron and [PO_4_] tetrahedron units. The bi‐octahedron units are connected by F atoms, while the [PO_4_] units are connected by oxygen atoms.^[^ ^121^, ^122^ ^]^ The charge and discharge process is usually accompanied by the redox of transition metal ions in the crystal structure, so the diffusion path of electrons in Na_3_V_2_(PO_4_)_2_F_3_ depends on the interconnection between the [V_2_O_8_F_3_] bi‐octahedron.^[^ ^123^ ^]^ Li et al.^[^ ^124^ ^]^ developed an AZIB based on a novel intercalated Na_3_V_2_(PO_4_)_2_F_3_ cathode, a carbon film functionalized Zn anode, as well as a 2 m Zn(CF_3_SO_3_)_2_ electrolyte. The zinc storage mechanism is illustrated in **Figure** **3**a. When the discharge rate increases sharply from 0.2 to 3 A g^−1^, the AZIB shows a good discharge platform and a small voltage drop (Figure 3b). Min Je Pai and coworkers^[^ ^125^ ^]^ focused on charge storage mechanisms of Non‐AZIBs and AZIBs with Na_3_V_2_(PO_4_)_2_F_3_ as cathode material. The electrochemical cycling and ex situ analyses of the Na_3_V_2_(PO_4_)_2_F_3_/C cathode reveal a completely contrasting electrochemical behavior between Non‐AZIBs and AZIBs due to the difference in the guest ion for the faradaic reactions during the cycle.

Na_3_V_2_(PO_4_)_2_F_3_ has been widely studied in materials with the molecular formula Na_3_V_2_(PO_4_)_2_F_3‐2y_O_2y_ (0≤y≤1). However, replacing part of F^−^ with O^2−^ reduces the induction effect caused by F^−^, resulting in higher ionic conductivity and lower polarization of Na_3_V_2_(PO_4_)_2_O_2_F composed of V^4+^. Wang and colleagues^[^ ^126^ ^]^ developed an AZIBs consisting of a novel nitrogen‐doped carbon inserted layered MoO_2_ material (MoO_2_@NC) as the intercalated anode and Na_3_V_2_(PO_4_)_2_O_2_F as the cathode. The preparation of MoO_2_@NC is shown in Figure 3c. The capacity of MoO_2_@NC||Zn‐ Na_3_V_2_(PO_4_)_2_O_2_F full cell remained at 78 mAh g^−1^ with a high‐capacity retention of 93% even after 200 cycles.

In addition, Na_3_V_2_(PO_4_)_2_O_1.6_F_1.4_ (V^3.8+^) provides an additional 0.4 electrons per formula unit compared to V^4+^/V^5+^ single‐electron transfer in Na_3_V_2_(PO_4_)_2_O_2_F.^[^ ^127^ ^]^ Therefore, this hybrid valency vanadium compound may exhibit a higher reversible capacity as cathode material of AZIBs. Ni et al.^[^ ^128^ ^]^ developed a neutral water‐in‐bisalts electrolyte of 25 mol kg^−1^ ZnCl_2_ + 5 mol kg^−1^ NH_4_Cl to enhance the electrochemical performance of Na_3_V_2_(PO_4_)_2_O_1.6_F_1.4_ coated with rGO (5 wt.%) as a noval AZIBs cathode. The crystal structure of Na_3_V_2_(PO_4_)_2_O_1.6_F_1.4_ is shown in Figure 3e. The PO_4_ tetrahedron and the VO_5_F/VO_4_F_2_ octahedron (including V^4+^ and V^3+^) share oxygen atoms to form an open framework. In addition, VO_5_F and VO_4_F_2_ octahedrons are bridged by F atoms. At 50 mA g^−1^, the reversible capacity is 155 mAh g^−1^ (Figure 3f), the average operating potential is 1.46 V, as well as the stable circulation can achieve 7000 cycles at 2 A g^−1^ (Figure 3g).

Na_3_MnTi(PO_4_)_3_, as a typical analog of sodium superconductor NASICON‐typed Na_3_V_2_(PO_4_)_3_, has been regarded as a promising cathode material. Although Na_3_MnTi(PO_4_)_3_ doesn't belong to vanadium‐based compounds, compared with vanadium, transition metals manganese and titanium have the advantages of low price, low toxicity, and rich resources.^[^ ^129^, ^130^, ^131^, ^132^ ^]^ The new environmentally friendly water‐based rechargeable hybrid sodium/zinc battery developed by Zhou et al.^[^ ^133^ ^]^ uses zinc as anode, Na_3_MnTi(PO_4_)_3_ as cathode, as well as 0.5 m CH_3_COONa and Zn (CH_3_COO)_2_ as mixed electrolytes. After 50 cycles at 1.5 C, the discharge capacity remains at 95.0 mAh g^−1^, the capacity retention rate is 84.6%, and the CE is 103.5% (Figure 3h). The schematic diagram (Figure 3i) of the electrochemical reaction between Zn//Na_3_MnTi(PO_4_)_3_ and every single electrode is shown below:

Cathode reaction:

(1) $$\begin{array}{ccc} {\mathrm{Na}}_{3}\mathrm{MnTi}\;{\left({\mathrm{PO}}_{4}\right)}_{3} & = & {\mathrm{Na}}_{x}\;\mathrm{MnTi}{\left({\mathrm{PO}}_{4}\right)}_{3}+\left(3-x\right){\mathrm{Na}}^{+}+\left(3-x\right)e^{-}\\ & & \left(\mathrm{first}\;\mathrm{charge}\;\mathrm{process}\right) \end{array}$$

(2) $$\begin{array}{ccc} & & {\mathrm{Na}}_{x}\mathrm{MnTi}{\left({\mathrm{PO}}_{4}\right)}_{3}+y{\mathrm{Na}}^{+}+z{\mathrm{Zn}}^{2+}+\left(y+2z\right)\;e^{-}\\ & & \;={\mathrm{Zn}}_{z}\;{\mathrm{Na}}_{\left(x+y\right)}\mathrm{MnTi}{\left({\mathrm{PO}}_{4}\right)}_{3}\\ & & \;\left(\mathrm{The}\;\mathrm{rest}\;\mathrm{discharge}/\mathrm{charge}\;\mathrm{processes}\right) \end{array}$$

(3) $$\begin{array}{ccc} & & \mathrm{Anodic}\;\mathrm{reaction}:\;{\mathrm{Zn}}^{2+}+2e^{-}\\ & & \;↔\mathrm{Zn}\;\left(\mathrm{overall}\;\mathrm{charge}/\mathrm{discharge}\;\mathrm{processes}\right) \end{array}$$

## 3 Vanadium Oxides

Vanadium oxides have been widely used due to their various oxidation states and large open crystal structure, which is favorable for metal ion (de)intercalation.^[^ ^137^, ^138^, ^139^ ^]^ Vanadium oxides have various oxidation states, different composition forms, and diverse coordination polyhedron, which provide many paths for Zn^2+^ (de)intercalation.^[^ ^140^ ^]^ In recent years, vanadium oxides have attracted much attention in AZIBs due to their high specific capacity, wide availability, and low cost.^[^ ^141^ ^]^ In addition, some electrochemical performance of vanadium oxides as cathode materials are shown in **Table** **2**.

**Table 2:** Electrochemical performance of vanadium oxides as cathodes in AZIBs

Materials	Electrolyte	Specific capacity [mAh g^−1^] (current density [A g^−1^])	Capacity retention (cycles numbers)	Voltage range [V]	Ref.
VO_2_	1 m ZnSO_4_	325.6 (0.05)	86% (5000)	0.2–1.2	[145]
VO_2_	1 m ZnSO_4_	353 (1)	75.5% (945)	0.2–1.3	[216]
Nsutite‐type VO_2_	3 m Zn(CF_3_SO_3_)_2_	314.4 (1)	84% (5000)	0.2–1.4	[217]
VO_2_(A)	3 m Zn(CF_3_SO_3_)_2_	400 (0.1)	29.3% (600)	0.2–1.4	[218]
VO_2_(A)@PPy	3 m Zn(CF_3_SO_3_)_2_	440 (0.1)	47.7% (860)	0.2–1.4	[218]
VO_2_(B)	3 m Zn(CF_3_SO_3_)_2_	357 (0.25 C)	91.2% (300)	0.3–1.5	[60]
VO_2_(B)/rGO	3 m Zn(CF_3_SO_3_)_2_	456 (0.1)	90% (1000)	0.2–1.4	[219]
VO_2_(D)	3 m ZnSO_4_	442(0.1)	30.5% (30 000)	0.2–1.5	[150]
O_d_‐HVO@PPy	2 m ZnSO_4_	337 (0.2)	77.2% (1000)	0.2–1.6	[220]
p‐V_2_O_3_@C	3 m Zn(CF_3_SO_3_)_2_	350 (0.1)	90% (4000)	0.3–1.5	[156]
V_2_O_3_	2 m Zn(CF_3_SO_3_)_2_	625 (0.1)	100% (10 000)	0.2–1.6	[157]
N@C/V_2_O_3_	3 m ZnSO_4_	451 (0.1)	92.95% (152)	0.3–1.6	[221]
V_2_O_5_	1 m Zn(ClO_4_)_2_	335 (0.05)	85% (5000)	0.4–1.6	[164]
V_2_O_5_	3 m Zn(CF_3_SO_3_)_2_	460 (0.5)	91.1% (4000)	0.2–1.6	[165]
V_2_O_5_	3 m ZnSO_4_	224 (0.1)	100% (400)	0.4–1.4	[166]
V_2_O_5_	2 m ZnSO_4_	341 (1)	84.3% (500)	0.2–1.6	[167]
V_2_O_5_	3 m Zn(CF_3_SO_3_)_2_	319 (0.02)	81% (500)	0.5–1.5	[168]
V_2_O_5_	21 m LiTFSI + 1 m Zn(CF_3_SO_3_)_2_	238 (0.05)	80% (2000)	0.2–1.6	[222]
O_d_‐V_2_O_5_	3 m Zn(CF_3_SO_3_)_2_	406 (0.1)	90% (1000)	0.1–1.6	[223]
O_d_‐V_2_O_5_	2 m Zn(CF_3_SO_3_)_2_	397.5 (0.2)	94.7% (5000)	0.4–1.5	[224]
V_2_O_5_@C	2.5 m Zn(CF_3_SO_3_)_2_	361.9 (0.5)	71% (2000)	0.2–1.5	[225]
V_2_O_5_/GO	3 m Zn(CF_3_SO_3_)_2_	525 (0.1)	90.8% (10 000)	0.3–1.6	[226]
PC/V_2_O_5_ *n*H_2_O	3 m Zn(CF_3_SO_3_)_2_	409.5 (0.5)	97.1% (1000)	0.3–1.6	[227]
V_3_O_7_ H_2_O	1 m ZnSO_4_	375 (1 C)	80% (200)	0.4–1.1	[201]
nanogrid‐ V_3_O_7_ H_2_O	3 m Zn(CF_3_SO_3_)_2_	481.3 (0.1)	85.4% (1000)	0.05–1.7	[228]
h‐VOW	2 m ZnSO_4_	455 (0.1)	85% (1200)	0.4–1.6	[229]
V_3_O_7_ H_2_O/Mxene	3 m Zn(CF_3_SO_3_)_2_	365.3 (0.2)	84% (5600)	0.2–1.6	[230]
V_5_O_12_ 6H_2_O	3 m Zn(CF_3_SO_3_)_2_	388 (0.2)	94% (1000)	0.2–1.6	[203]
V_5_O_12_ 6H_2_O‐LGO	3 m Zn(ClO_4_)_2_	467 (0.1)	96.6% (3500)	0.2–1.6	[231]
Z‐V_5_O_12_ 6H_2_O	2 m ZnSO_4_	328 (0.05)	80.4% (1500)	0.2–1.6	[232]
V_6_O_13_	1 m Zn(CF_3_SO_3_)_2_	≈360 (0.2)	92% (2000)	0.2–1.5	[179]
V_6_O_13_	3 m Zn(CF_3_SO_3_)_2_	450 (0.1)	80% (3000)	0.3–1.4	[180]
V_6_O_13_/CC	3 m ZnSO_4_	520 (0.5)	85.3% (1000)	0.2–1.4	[233]
V_6_O_13_@gCC	3 m ZnSO_4_	290 (0.375)	99% (1000)	0.2–1.4	[70]
O_d_‐V_6_O_13_@C	3 m Zn(TFSI)_2_	401 (0.2)	86% (2000)	0.2–1.5	[234]
DNGS	ZnSO_4_ + Na_2_SO_4_ + H_3_BO_3_ mixed solution	403.5 (0.2)	94% (2000)	0.2–1.5	[235]
VCF	3 m Zn(CF_3_SO_3_)_2_	371 (0.2)	91% (5000)	0.3–1.5	[236]
CO_2_‐V_6_O_13_	3 m Zn(CF_3_SO_3_)_2_	471 (0.1)	80% (4000)	0.3–1.5	[237]
V_6_O_13_ *n*H_2_O	3 m Zn(CF_3_SO_3_)_2_	386 (0.3)	87% (1000)	0.2–1.4	[205]
V_10_O_24_ 12H_2_O	2 m ZnSO_4_	327 (0.1)	57.5% (3000)	0.5–1.5	[238]
dendritic V_10_O_24_ 12H_2_O	3 m Zn(CF_3_SO_3_)_2_	164.5 (0.2)	80.1% (3000)	0.7–1.7	[206]
Al‐doped V_10_O_24_ 12H_2_O	3 m Zn(CF_3_SO_3_)_2_	415 (0.2)	98% (3000)	0.3–1.6	[208]
V_10_O_24_ 12H_2_O@C	3 m Zn(CF_3_SO_3_)_2_	290.5 (0.5)	94.1% (10 000)	0.3–1.5	[209]

### 3.1 VO_2_

**Figure 4:**
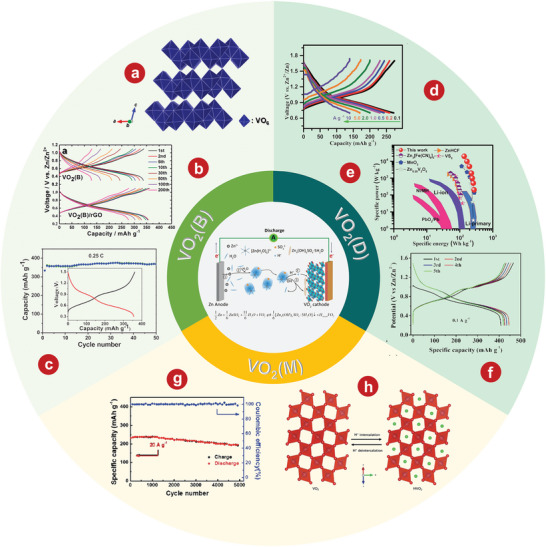
a) The crystal structure of VO_2_(B). b) Continuous cycles of bare VO_2_ and the VO_2_/rGO composite. c) Discharge–charge profiles obtained at different current densities from 0.1 to 10 A g^−1^. e) Comparison of the Ragone plots of the VO_2_‐based ZIB and reported energy storage systems. f) The first five galvanostatic charging/discharging curves at 0.1 A g^−1^. g) Corresponding discharge/charge profiles at various current densities. h) Schematic illustration of the proton insertion and deinsertion into VO_2_(M). a, b) Reproduced with permission.^[^ ^144^ ^]^ Copyright 2018, American Chemical Society. c) Reproduced with permission.^[^ ^60^ ^]^ Copyright 2018, WILEY‐VCH. d, e) Reproduced with permission.^[^ ^147^ ^]^ Copyright 2018, The Royal Society of Chemistry. f) Reproduced with permission.^[^ ^150^ ^]^ Copyright 2019, The Royal Society of Chemistry. g, h) Reproduced with permission.^[^ ^153^ ^]^ Copyright 2020, The Royal Society of Chemistry.

VO_2_ is a kind of metal oxide with phase transformation. The structure changes before and after phase transformation lead to the reversible transformation of infrared light from transmission to reflection.^[^ ^142^ ^]^ In addition, VO_2_ has d^1^ electronic system, and there are many different crystal types, including thermodynamically stable rutile VO_2_(R), monoclinic VO_2_(M) and metastable tetragonal VO_2_(A), monoclinic VO_2_(B), VO_2_(C), and VO_2_(D).^[^ ^143^ ^]^ Although the chemical formula is the same, their crystal structure and electronic structure are completely different and complicated, and the application scenarios are different. Metastable monoclinic VO_2_(B) has been widely used as a cathode material for AZIBs due to its open framework.^[^ ^144^, ^145^, ^146^ ^]^ In 2018, Park et al.^[^ ^144^ ^]^ first proposed using VO_2_(B) as a cathode material of AZIBs and verified its feasibility through first‐principles calculation. The crystal structure of VO_2_(B) is shown in **Figure** **4**a. The team successfully synthesized VO_2_(B) by low‐temperature solvothermal method, which was then combined with rGO to form VO_2_(B)/rGO.^[^ ^144^ ^]^ Electrochemical tests were carried out on the VO_2_(B) and VO_2_(B)/rGO composite materials, and it was obvious that the electrochemical performance of the composite materials was significantly improved by rGO (Figure 4b). By in situ XRD and various electrochemical measurements, Ding and colleagues^[^ ^60^ ^]^ demonstrated the pseudo‐capacitance behavior and ultra‐fast kinetics of a unique tunnel of zinc ions embedded VO_2_(B) nanofibers in an aqueous Zn(CF_3_SO_3_)_2_ electrolyte. The as‐prepared VO_2_(B) nanofiber cathode has a highly stable reversible capacity of 357 mAh g^−1^ at 0.25 C (Figure 4c).

Metastable VO_2_(D) has also been synthesized as a cathode material for AZIBs. Wei et al.^[^ ^147^ ^]^ first studied the zinc storage performance of metastable VO_2_(D) as a cathode material of ZIBs. Their team demonstrated an interesting electrochemically induced phase transition from monoclinal VO_2_ to bilayer V_2_O_5_ *n*H_2_O, with significantly increased interlayer spacing and reduced structural order during the initial (de)intercalation of Zn^2+^, with good structural stability in subsequent cycles. The corresponding discharge/charge profiles show the VO_2_(D) cathode has superior rate performance, among which the best capacity is 274mAh g^−1^ at 0.1A g^−1^ (Figure 4d). Furthermore, by measuring and comparing the power and energy density of state‐of‐the‐art power supplies (Figure 4e), it can be seen that the VO_2_‐based AZIBs system provides excellent electrochemical performance.^[^ ^46^, ^59^, ^148^, ^149^ ^]^ Chen and colleagues^[^ ^150^ ^]^ developed VO_2_(D) hollow nanospheres as ZIBs cathode materials, which have a high reversible discharge capacity of 408 mAh g^−1^ at 0.1 A g^−1^ in 3 m ZnSO_4_ electrolyte (Figure 4f), and long cyclic endurance stability stable can reach up to 30 000 cycles with the capacity attenuation rate of 0.0023% each cycle.

VO_2_(M) is composed of twisted [VO_6_] octahedrons, in which [VO_6_] octahedrons are staggered and connected into a network by sharing O atoms, forming a tunnel about 0.318 nm long.^[^ ^151^ ^]^ Importantly, VO_2_(M) phase can be obtained by a simple heat treatment of VO_2_(B).^[^ ^152^ ^]^ Compared with VO_2_(B) and VO_2_(D), VO_2_(M) has a denser tunnel and higher space utilization, which facilitates ion migration. In addition, it is found that VO_2_(M) has better thermal stability. Zhang et al.^[^ ^153^ ^]^ first prepared VO_2_(M) integrated with carbon nanotubes (CNTs) as AZIBs cathode. As shown in Figure 4g, the as‐prepared binder‐free cathode delivers excellent stability with 84.5% retention after 5000 cycles at 20 A g^−1^. The electrochemical reaction of the Zn/VO_2_(M) battery (Figure 4h) in the aqueous ZnSO_4_ electrolyte solution is as follows:

(4) $$\mathrm{Anode}:\;\mathrm{Zn}↔{\mathrm{Zn}}^{2+}+2e^{-}$$

(5) $$\mathrm{Cathode}:\;H^{+}+e^{-}+{\mathrm{VO}}_{2}↔{\mathrm{HVO}}_{2}$$

### 3.2 V_2_O_3_

**Figure 5:**
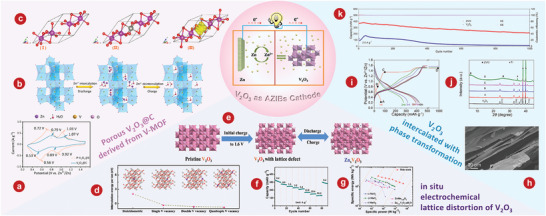
a) Peak potential separations in the CV curves of P‐V_2_O_3_@C and V_2_O_3_@C. b) Schematic illustrations of Zn^2+^ storage mechanism of V_2_O_3_ cathode during the processes of discharge and charge. c) Structure of V_2_O_3_ without (I) and with (II) Zn ions being inserted. (III) Differential charge state density between V_2_O_3_ with and without Zn ions being inserted. Red balls denote O atoms, purple balls denote V atoms, and gray balls denote Zn ions. d) DFT calculated intercalation energies for zinc ions in stoichiometric V_2_O_3_ and V_2_O_3_ with different vacancies. The inset images show the corresponding models of V_48_O_72_ (defect‐free), V_47_□_1_O_72_ (one‐vacancy), V_46_□_2_O_72_ (two‐vacancy), and V_44_□_4_O_72_ (quadruple‐vacancy). e) Schematic illustration of the initial charging process for V2O3 and the subsequent Zn^2+^ storage in V_2_O_3_ with lattice defects. f) Rate performance of V_2_O_3_ at various rates from 0.4 to 51.2 A g^−1^. g) Ragone plots of V_2_O_3_ compared with reported cathodes. h) The scanning electron microscopy (SEM) image of V_2_O_3_ nanosheets. i) The typical charge/discharge profiles of V_2_O_3_. j) The XRD patterns of V_2_O_3_ electrode during the initial cycle. k) The cyclability performance of Zn_x_V_2_O_5_‐*n*H_2_O electrode at 2.0 A g^−1^. a–c) Reproduced with permission.^[^ ^156^ ^]^ Copyright 2019, American Chemical Society. e–g) Reproduced with permission.^[^ ^159^ ^]^ Copyright 2021, Wiley‐VCH. h–k) Reproduced with permission.^[^ ^161^ ^]^ Copyright 2021, Elsevier.

V_2_O_3_ is considered as a high‐capacity electrochemical energy storage material because it shares an edge with the adjacent octahedron through two common VO_6_ octahedra to form a 3D structure‐like tunnel, which is conducive to the intercalation of cation.^[^ ^154^, ^155^ ^]^ Vanadium 3d electrons can transfer along the V‐V chain and generally exhibit higher electronic conductivity than most transition metal oxides, which is conducive to the development of V_2_O_3_ as AZIBs cathode with excellent electrochemical performance.^[^ ^64^ ^]^ Ding et al.^[^ ^156^ ^]^ prepared a porous V_2_O_3_@C hybrid nanostructure (P‐V_2_O_3_@C) with high conductivity by pyrolyzing V‐MOF precursor and further illustrated its application as a intercalated cathode for AZIBs. The corresponding peak potential separation (ΔV) to P‐V_2_O_3_@C at 0.2 mV s^−1^ is significantly smaller than V_2_O_3_@C, indicating that the P‐V_2_O_3_@C cathode is more stable than V_2_O_3_@C cathode (**Figure** **5**a). The electrochemical reactions in new Zn// V_2_O_3_ battery system are shown in Figure 5b and described by the following equation:

(6) $$\mathrm{Cathode}:\;V_{2}O_{3}+x{\mathrm{Zn}}^{2+}+nH_{2}O+2xe^{-}={\mathrm{Zn}}_{x}\;V_{2}O_{3}\cdot nH_{2}O$$

(7) $$\mathrm{Anode}:\;{\mathrm{Zn}}^{2+}+2e^{-}=\mathrm{Zn}$$

The intercalation behavior of Zn^2+^ in V_2_O_3_ is verified by first‐principles calculations (Figure 5c). As the charge around the zinc ion increases, so does the number of electrons between the zinc atom and the oxygen atom.

The storage of Zn ions by V_2_O_3_ is achieved through the complete phase transformation with the product of V_2_O_5_.^[^ ^157^, ^158^ ^]^ However, the exact electrochemical behavior of V_2_O_3_ in a weak acid solution containing Zn^2+^ is not clear. Ding and colleagues^[^ ^159^ ^]^ studied a novel in situ electrochemical conversion reaction of V_2_O_3_, and the resulting product can be used as a cathode for ultra‐fast Zn^2+^ (de)intercalation. Operando XRD and Operando Raman spectroscopy confirmed the unique lattice conversion reaction of V_2_O_3_ during initial charging. As shown in Figure 5d, the corresponding single‐vacancy, double‐vacancy, and four‐vacancy systems consist of V_47□1_O_72_, V_46□2_O_72_, and V_44□4_O_72_, where □ represents cationic vacancies. The density functional theory (DFT) calculations show that the intercalation energy of Zn^2+^ decreases from −0.28 to −0.72 eV when the number of empty spaces increases from 1 to 4, which is lower than stoichiometry V_2_O_3_ (2.06 eV). This indicates that vanadium vacancies in V_2_O_3_ facilitate the intercalation of multivalent Zn^2+^. As shown in Figure 5e, the reversible process directly indicates that V_2_O_3_ has superior ability of (de)insertion. The unique in situ electrochemical lattice conversion reaction allows the V_2_O_3_ cathode to achieve a high reversible capacity of 328 mAh g^−1^ at 0.8 A g^−1^ (Figure 5f), which is higher than some reported cathodes (Figure 5g).^[^ ^46^, ^59^, ^160^ ^]^ In addition, Hu et al.^[^ ^161^ ^]^ prepared a metallic V_2_O_3_ material with intercalated phase transition as cathode for application in AZIBs. The prepared V_2_O_3_ is composed of 2D nanosheets with large sizes (Figure 5h). The significant change in the cyclic voltammetry (CV) curve during the cycle indicates the phase transition during the cycle (Figure 5i). In order to explore the phase evolution mechanism of V_2_O_3_, ex situ XRD tests are performed at selected states and different cycling stages (Figure 5j). After 1000 cycles, the Zn_x_V_2_O_5_ *n*H_2_O electrode still has a high reversible specific capacity of 218 mAh g^−1^ and the coulombic efficiency (CE) is close to 100% (Figure 5k).

### 3.3 V_2_O_5_

**Figure 6:**
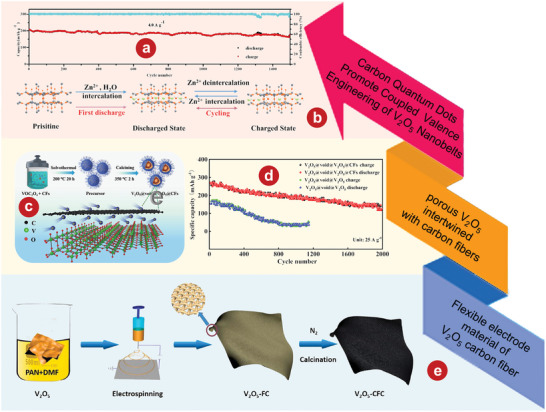
a) Cycling performance and CE of V_2_O_5_/CQDs at the current density of 4 A g^−1^. b) Schematic illustration of Zn^2+^ and water co‐intercalation into V_2_O_5_/CQDs electrode during the initial discharge process and reversible Zn^2+^. c) Illustration of synthetic procedure of V_2_O_5_@void@V_2_O_5_@CFs hybrid. d) Cycling performance comparison of V_2_O_5_@void@V_2_O_5_ and V_2_O_5_@void@V_2_O_5_@CFs electrode at 25 A g^−1^. e) Mechanism for the development of flexible V_2_O_5_‐CFC cathode material. a, b) Reproduced with permission.^[^ ^173^ ^]^ Copyright 2021, Wiley‐VCH. c, d) Reproduced with permission.^[^ ^174^ ^]^ Copyright 2022, Elsevier. e) Reproduced with permission.^[^ ^175^ ^]^ Copyright 2022, Elsevier.

V_2_O_5_ is a typical layered vanadium compound, in which V and O atoms form [VO_5_] square pyramid, and then through co‐edges or co‐corners form a layered structure.^[^ ^162^ ^]^ The adjacent layers are also connected by van der Waals force and H bonds between the layers.^[^ ^163^ ^]^ In addition, the layer spacing is about 0.58 nm, much larger than the radius of Zn^2+^ (0.76 Å), which is conducive to the diffusion of Zn^2+^ between V_2_O_5_ layers.^[^ ^164^ ^]^ V_2_O_5_ occurs two‐electron redox reaction and can provide a high theoretical zinc storage capacity of 589 mAh g^−1^ in the process of (dis)charge.^[^ ^165^ ^]^ However, the large polarization and volume variation caused by the insertion of Zn^2+^ as a multivalent carrier into the cathode host remains a major obstacle to the development of AZIBs with high performance.^[^ ^148^ ^]^ In addition, V_2_O_5_ has the disadvantage of low electrical conductivity, which hinders its large‐scale development.^[^ ^166^ ^]^ Therefore, it is necessary to find ways to enhance electronic conductivity. So far, researchers have reported a variety of V_2_O_5_ materials.^[^ ^167^, ^168^, ^169^, ^170^ ^]^ Recently, many researchers have enhanced the electronic conductivity of V_2_O_5_ by combining it with carbonaceous functional materials (e.g., carbon nanotubes, carbon nanofibers, and carbon quantum dots (CQDs)).^[^ ^171^, ^172^ ^]^ Zhang et al.^[^ ^173^ ^]^ synthesized V_2_O_5_ nanobelts induced by CQDs by a simple one‐step hydrothermal method. V_2_O_5_/CQDs composite as the cathode of ZIBs shows good stable cycle performance which maintains 85% capacity at 4 A g^−1^ over 1500 cycles. (**Figure** **6**a). Zhang et al.^[^ ^173^ ^]^ also studied its electrochemical kinetics and zinc ion storage mechanism (Figure 6b). The chemical reactions of cathode are as follows:

(8) $$\begin{array}{ccc} & & \mathrm{In}\;\mathrm{first}\;\mathrm{discharge}\;\mathrm{process}:\\ & & \;V_{2}O_{5}\cdot nH_{2}O/\mathrm{CQDs}+x{\mathrm{Zn}}^{2+}+2xe^{-}+\left(m-n\right)H_{2}O\\ & & \;={\mathrm{Zn}}_{x}V_{2}O_{5}\cdot mH_{2}O/\mathrm{CQDs} \end{array}$$

(9) $$\begin{array}{ccc} & & \mathrm{In}\;\mathrm{subsequent}\;\mathrm{cycles}:\\ & & \;{\mathrm{Zn}}_{x}V_{2}O_{5}\cdot mH_{2}O/\mathrm{CQDs}={\mathrm{Zn}}_{y}V_{2}O_{5}\cdot mH_{2}O/\mathrm{CQDs}\\ & & \;+\left(x-y\right){\mathrm{Zn}}^{2+}+2\left(x-y\right)e^{-} \end{array}$$

In order to give full play to the electrochemical properties of V_2_O_5_, Xiong et al.^[^ ^174^ ^]^ first used a simple combination of hydrothermal and calcination method to construct in situ a sphere‐ball porous V_2_O_5_ (V_2_O_5_@‐void@V_2_O_5_@CFs) by wrapping carbon fibers (CFs) (Figure 6c). V_2_O_5_@‐void @V_2_O_5_@CFs electrode to realize the high capacity of 149 mAh g^−1^ at 25 A g^−1^ after 2000 cycles (Figure 6d). The excellent energy storage performance of V_2_O_5_@‐void@V_2_O_5_@CFs electrode is attributed to its unique architecture. The CFs in the composite act as strong shells and conductive bridges connecting the independent V_2_O_5_ units, preventing the isolation of adjacent spheres, and providing fast interconnect paths for electrons and ions in electrochemical dynamics. In addition, Xu et al.^[^ ^175^ ^]^ developed a novel vanadium‐based AZIB by combining V_2_O_5_ with carbon fiber cloth (V_2_O_5_‐CFC) by electrospinning (Figure 6e), which had excellent flexibility and was designed with no binder and no collection device. The composite fiber structure can avoid the coacervation of V_2_O_5_ nanosheets and reduce the volume effect in the (dis)charging process, which makes V_2_O_5_‐CFC deliver excellent electrochemical properties.

### 3.4 V_6_O_13_

**Figure 7:**
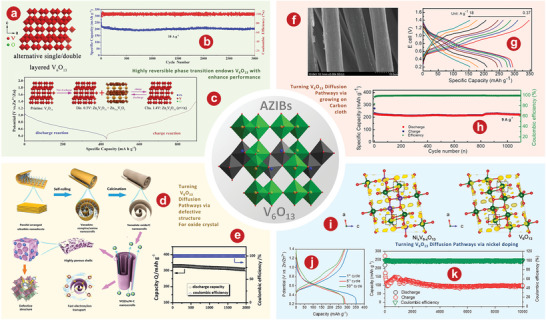
a) The crystal structures of V_6_O_13_. b) The cyclic performance at 10 A g^−1^ of V_6_O_13_ product. c) Schematic illustration of highly reversible phase transition during discharge‐charge process of V_6_O_13_. d) Schematic illustration of the synthesis and structure of the (V_6_O_13‐*δ*_)/C nanoscrolls. e) High‐rate long‐term cycling properties of the (V_6_O_13‐*δ*_)/C sample at 10 A g^−1^. f) SEM image of V_6_O_13_@gCC. g) Discharge/charge profile of V_6_O_13_@gCC cathode at different current rates. h) Cycling performance at a current density of 9 A g^−1^. i) The differential charge density maps for Ni_x_V_6‐x_O_13_ atomic layer slab and V_6_O_13_ atomic layer slab with the iso‐surface level of 0.03 e Å^−3^. In the differential density map, yellow region indicates electro accumulation. Red O, green V, and pink Ni atoms. j) Charge/discharge profiles of Zn//Ni_0.24_V_5.76_O_13_ batteries at 1.0 A g^−1^. k) Cycling performance of Zn//Ni_0.24_V_5.76_O_13_ batteries at 8.0 A g^−1^. a–c) Reproduced with permission.^[^ ^180^ ^]^ Copyright 2019, Wiley‐VCH. d, e) Reproduced with permission.^[^ ^181^ ^]^ Copyright 2020, Wiley‐VCH. f–h) Reproduced with permission.^[^ ^70^ ^]^ Copyright 2020, Elsevier. i–k) Reproduced with permission.^[^ ^183^ ^]^ Copyright 2022, Elsevier.

With perovskite‐like framework structure, monoclinic V_6_O_13_ is composed of twisted VO_6_ octahedrons arranged in a zigzag shape with a corporate edge or a corporate angle to form single and double layers.^[^ ^176^, ^177^ ^]^ The V(1) sites of the monolayer and the V(3) sites of the bilayer are occupied by V^4+^, and the V(2) sites of the bilayer have the V^5+^ characteristic.^[^ ^141^, ^178^ ^]^ V_6_O_13_ material with a theoretical capacity of 417 mAh g^−1^ at 900 Wh kg^−1^ has been studied as cathode of LIBs due to its high conductivity at room temperature.^[^ ^177^ ^]^ In addition, V_6_O_13_ has significant potential as a high‐performance cathode material of AZIBs due to its special 3D open framework structure that can be used for Zn^2+^ (de)intercalation.^[^ ^179^ ^]^ Shan et al.^[^ ^180^ ^]^ proved that V_6_O_13_ had better Zn^2+^ storage performance as a cathode for AZIBs by comparing it with VO_2_ and V_2_O_5_. The crystal structures of V_6_O_13_ (**Figure** **7**a) provide additional active sites for Zn^2+^ storage. Figure 7b delivers that V_6_O_13_ can show excellent long‐term cycling of 206 mA h^−1^ after 3000 cycles at 10 A g^−1^. The schematic diagram of the energy storage mechanism of Zn/V_6_O_13_ aqueous battery is shown in Figure 7c.

Recently, researchers also modified the properties of V_6_O_13_ through various schemes. Lin and colleagues^[^ ^181^ ^]^ prepared defective vanadate oxide (V_6_O_13‐*δ*_)/C (DVOC) nanoscrolls used as cathode materials for AZIBs due to their large spacing (Figure 7d). DVOC has good long‐term cycle stability and stable charge and discharge performance. After 2000 cycles, at A current density of 10 A g^−1^, the CE of the DVOC is close to 100%, and 93.4% of the initial capacity is retained (Figure 7e). In addition, Tamilselvan et al.^[^ ^70^ ^]^ coaxially grew interconnected V_6_O_13_ nanobelts on carbon cloth fibers (V_6_O_13_@gCC) through a facile hydrothermal process. SEM image of V_6_O_13_@gCC is shown in Figure 7f. As shown in Figure 7g, the current density of V_6_O_13_@gCC electrode increased from 0.375 to 18 A g^−1^, following the discharge capacity decrease from 290 mAh g^−1^ to 154 mAh g^−1^. The V_6_O_13_@gCC electrode obtained an initial specific capacity of 227 mAh g^−1^ at 9 A g^−1^ and retained nearly 99% after 1000 cycles (Figure 7h).

Metallic ion doping is also proven to be effective to enhance the electrochemical performance o V_6_O_13_.^[^ ^182^ ^]^ Our group.^[^ ^183^ ^]^ synthesized nickel‐ion doped V_6_O_13_ (Ni_x_V_6‐x_O_13_) layers with abundant reaction sites, high spacing, and high conductivity, and also verified their feasibility as cathode for AZIBs. Figure 7i shows that carriers can be more efficiently transferred to the minimum conduction band of Ni_x_V_6‐x_O_13_ layers compared with V_6_O_13_, which is conducive to the transport of Zn^2+^.^[^ ^184^, ^185^ ^]^ Ni_0.24_V_5.76_O_13_ electrode shows the best electrochemical performance with the discharge capacitance of 302.6 mAh g^−1^ at 1 A g^−1^ (Figure 7j). In addition, the Ni_0.24_V_5.76_O_13_ cathode can reach a capacity of 96.5 mA h g^−1^ with a CE of 99.01% after 10 000 cycles at 8.0 A g^−1^ (Figure 7k).

### 3.5 The Intercalation of H_2_O

Vanadium oxides are one of the most promising cathodes for AZIBs due to their diversity in composition and crystal structure. However, they still have some problems, for instance, the slow electrochemical diffusion kinetics and limited reversibility seriously hinder their wide applications. Structural H_2_O molecules which are in the layers of vanadium oxides have been studied because hydrated vanadium oxides can reversibly absorb more guest ions than dehydrated vanadium oxides, due to the presence of structural H_2_O molecules between the layers, thus expanding the ion intercalation space.^[^ ^186^ ^]^ Furthermore, the structural H_2_O molecules provide charge shielding to reduce electrostatic electric interactions between intercalated Zn^2+^ and the host materials, thereby allowing rapid Zn^2+^ diffusion.^[^ ^187^ ^]^

#### 3.5.1 V_2_O_5_ *n*H_2_O

**Figure 8:**
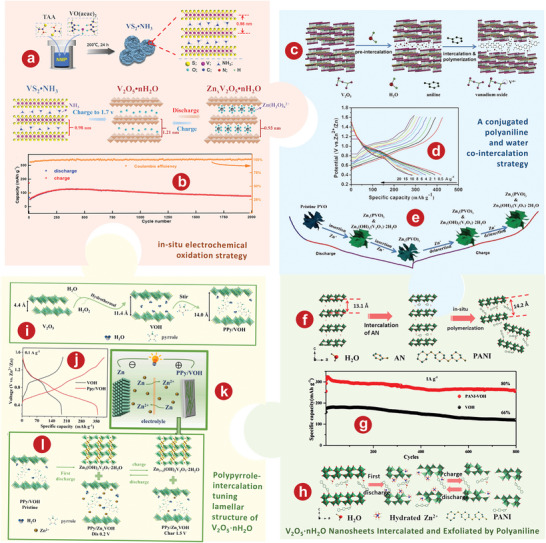
a) Schematic illustration of the synthesis of interlayer‐expanded flower‐like VS_2_ NH_3_ hollow spheres, as well as the electrochemical oxidation of VS_2_ NH_3_ process and the subsequent Zn^2+^ storage mechanism. b) Long‐term stability at 3.0 A g^−1^. c) Schematic diagram of the preparation processes of the PVO architectures. d) Discharge–charge curves at current densities ranging from 0.5 to 20 A g^−1^. e) Schematic reversible storage mechanism of zinc species in the PVO cathode. f) Schematic illustration of the fabrication process for PANI–VOH. g) Cycling performance of PANI–VOH and V_2_O_5_ *n*H_2_O at 1 A g^−1^. h) Schematic illustration of the (dis)charging reaction mechanism. i) The diagram of synthesis of PPy/VOH composite. j) Specific capacity between V_2_O_5_ *n*H_2_O and PPy/VOH at 0.1 A g^−1^. k) The schematic configuring of metallic zinc//PPy/VOH AZIBs. l) Schematic diagram of the zinc (de)intercalation mechanism in the PPy/VOH cathode. a, b) Reproduced with permission.^[^ ^192^ ^]^ Copyright 2022, Elsevier. c–e) Reproduced with permission.^[^ ^194^ ^]^ Copyright 2019, The Royal Society of Chemistry. f–h) Reproduced with permission.^[^ ^195^ ^]^ Copyright 2020, American Chemical Society. i–l) Reproduced with permission.^[^ ^196^ ^]^ Copyright 2022, Elsevier.

Different from the above V_2_O_5_ with a typical layered structure, V_2_O_5_ *n*H_2_O is composed of two [VO_6_] octahedral layers, showing a special double‐layer structure. The water molecules are usually in the middle of the two layers and act as props, providing greater spacing between the inner layers.^[^ ^61^, ^188^ ^]^ In addition, the intercalation of water molecules can also reduce the effective charge of Zn^2+^, which makes the process of Zn^2+^ deintercalation easier to carry out, thus showing better electrochemical performance.^[^ ^189^, ^190^ ^]^ Huang et al.^[^ ^191^ ^]^ prepared a freestanding V_2_O_5_ *n*H_2_O/CNTs film and applied it as AZIBs cathode in aqueous/organic hybrid electrolytes. In the hybrid electrolytes, the AZIB based on this cathode has excellent performance with an energy density of 102 Wh kg^−1^ at high power of 1500 W kg^−1^. Yang et al.^[^ ^192^ ^]^ oxidized interlayer‐expanded VS_2_ NH_3_ hollow spheres to prepare V_2_O_5_ *n*H_2_O with decreased nanometer size and ordered porous structure through in situ electrochemical oxidation strategies, which provides abundantly accessible sites and promotes the Zn^2+^ diffusion process (**Figure** **8**a). This V_2_O_5_ *n*H_2_O cathode derived from VS_2_ NH_3_ shows an excellent long cyclic stability of 110% capacity retention after 2000 cycles at 3 A g^−1^ (Figure 8b).

#### 3.5.2 The Intercalation of Organic Molecules in V_2_O_5_ *n*H_2_O

It's found that the intercalation of conductive organic polymer (e.g., polyaniline, polypyrrole) into V_2_O_5_ *n*H_2_O can enlarge the mesoporous, and improve the electrical conductivity of the nanocomposites, thus enhancing the performance of AZIBs.^[^ ^193^ ^]^ Zeng et al.^[^ ^194^ ^]^ employed a conjugated polymer and water co‐intercalation strategy to greatly improve the kinetics of Zn^2+^ diffusion in rose‐like V_2_O_5_ architectures (PVO) (Figure 8c). The cathode based on PVO shows a further enhanced rate performance of 288.9 mA h g^−1^ at 20 A g^−1^ (Figure 8d). Figure 8e shows the reversible storage and formation mechanism of Zn^2+^ in PVO cathode. Wang et al.^[^ ^195^ ^]^ enhanced electrochemical dynamics and stability of V_2_O_5_ *n*H_2_O, which was realized by pre‐intercalation of aniline monomer and in situ polymerization in the oxide interlayers (PANI‐VOH), as shown in Figure 8f. Compared to 255 mAh g^−1^ in the first cycle, the reversible capacity of PANI‐VOH increases to 323 mAh g^−1^ after 6 cycles, possibly because of the improved wetting by electrolyte or material utilization (Figure 8g). The electrochemical mechanism of PANI‐VOH was finally obtained by Zeng et al.^[^ ^194^ ^]^ through analyzing the data shown in Figure 8h. In addition to PANI, the intercalation of PPy can greatly expand the layer spacing of the layered structure, effectively reduce the internal resistance of the main material, accelerate the ion transport speed, and improve the specific volume and structural properties of the V_2_O_5_ *n*H_2_O. As shown in Figure 8i, Feng et al.^[^ ^196^ ^]^ intercalated the conducting PPy into the V_2_O_5_ *n*H_2_O layers to modulate the structure of the layered V_2_O_5_ *n*H_2_O (PPy/VOH). PPy/VOH has a specific capacity of 383 mAh g^−1^ at 0.1 A g^−1^, however, under the same conditions, the specific capacity of V_2_O_5_ *n*H_2_O is only 168 mAh g^−1^ (Figure 8j). The PPy/VOH cathode is assembled into AZIB, as shown in Figure 8k. On the basis of the experimental results of Feng et al.,^[^ ^196^ ^]^ the electrochemical mechanism of Zn//PPy/VOH battery is shown in Figure 8l.

#### 3.5.3 V_3_O_7_ H_2_O

**Figure 9:**
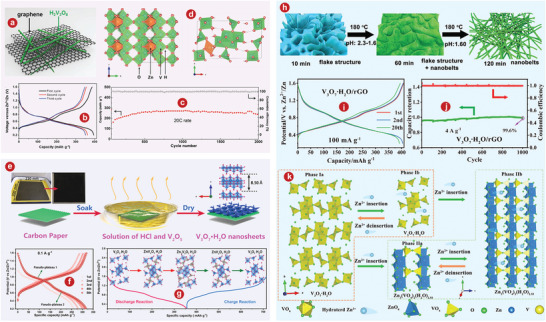
a) Schematic illustration of the structure of the H_2_V_3_O_8_ nanowires/graphene composite. b) Galvanostatic charge‐discharge profiles of the H_2_V_3_O_8_/graphene cathode at 0.1 A g^−1^. c) Long cycling stability at 20 C rate (6 A g^−1^). d) Potential intercalated Zn sites in the H_2_V_3_O_8_ crystal viewed along the [100] direction (left) and along the [001] direction (right). e) Schematic of the one‐step synthesis process for the binder‐free electrode material. f) Galvanostatic charge‐discharge profiles at 0.1 A g^−1^. g) Schematic of the stepwise uptake/extraction process of Zn^2+^ in different discharge/charge depths. h) Diagram illustrating the formation process of V_3_O_7_ H_2_O nanobelts. i) V_3_O_7_ H_2_O/rGO at a current density of 0.1 A g^−1^. j) Long‐term cycling performance of the V_3_O_7_ H_2_O/rGO at a current density of 4 A g^−1^. k) Illustration of the phase transition during Zn^2+^ (de)intercalation. The conversion in the pink and green dash box governs the initial cycles and long‐term cycling, respectively. a–d) Reproduced with permission.^[^ ^200^ ^]^ Copyright 2018, WILEY‐VCH. e–g) Reproduced with permission.^[^ ^201^ ^]^ Copyright 2021, Elsevier. h–k) Reproduced with permission.^[^ ^202^ ^]^ Copyright 2021, WILEY‐VCH.

For V_3_O_7_⋅H_2_O (H_2_V_3_O_8_) crystal structure, VO_6_ octahedrons are connected to each other by corners and edges, and link with other VO_5_ square pyramids to form 2D V_3_O_8_ layers. The bc‐plane⋅H_2_O molecules bonded with V atoms in VO_5_ polyhedrons are on both sides of V_3_O_8_ layers.^[^ ^197^, ^198^ ^]^ The two adjacent layers of V_3_O_8_ are connected by H bonds. Due to the vibration of the H bonds, V_3_O_8_ gets buffer layer space.^[^ ^199^ ^]^ In the process of (de)intercalation of Zn^2+^, cell distortion is relatively easy to occur without damaging the crystal structure. There are particular H bonds in V_3_O_7_ H_2_O that can accommodate the volume change during Zn^2+^ (de)intercalation. Pang et al.^[^ ^200^ ^]^ developed a composite of H_2_V_3_O_8_ nanowires coated with graphene sheets (**Figure** **9**a) as a cathode material for AZIBs. H_2_V_3_O_8_ nanowires/graphene composite delivers a remarkable Zn^2+^ storage performance with a high capacity of 394 mAh g^−1^ at 0.1 A g^−1^ (Figure 9b) and excellent retained capacity of 87% after 2000 cycles (Figure 9c) due to the synergistic effect between the structural characteristic of H_2_V_3_O_8_ nanowires and the high conductivity of graphene networks. Pang et al.^[^ ^200^ ^]^ also showed by DFT calculations that zinc is stable at the vacancy center and slightly distorts neighboring vanadium atoms (Figure 9d). Chen and colleagues^[^ ^201^ ^]^ systematically investigated the Zn^2+^ intercalation process in V_3_O_7_ H_2_O and first discovered two‐step Zn^2+^ intercalation mechanism in V_3_O_7_ H_2_O. The pathway for V_3_O_7_ H_2_O synthesis is shown in Figure 9e. Figure 9f delivers two pairs of pseudo‐platforms at 0.95/0.98 and 0.88/0.65 V, indicating that V_3_O_7_ H_2_O will undergo a two‐step process of Zn^2+^ (de)intercalation during (dis)charge. Based on the two‐step Zn^2+^ intercalation mechanism, the structural evolution of a cathode material under different (dis)charge depths is shown in Figure 9g.

(10) $$V_{3}O_{7}\cdot H_{2}O+{\mathrm{Zn}}^{2+}+2\;e^{-}={\mathrm{ZnV}}_{3}O_{7}\cdot H_{2}O\;\left(\mathrm{First}\;\mathrm{step}\right)$$

(11) $$\begin{array}{c} {\mathrm{ZnV}}_{3}O_{7}\cdot H_{2}O+{\mathrm{Zn}}^{2+}+2\;e^{-}={\mathrm{Zn}}_{2}\;V_{3}O_{7}\cdot H_{2}O\;\left(\mathrm{Second}\;\mathrm{step}\right) \end{array}$$

Cao et al.^[^ ^202^ ^]^ developed a microwave‐assisted method to one‐step prepare V_3_O_7_∙H_2_O nanobelts/rGO composite through controlling pH with acids (Figure 9h). The V_3_O_7_∙H_2_O nanobelts/rGO composite shows excellent performance with a high initial capacity of 404.7 mAh g^−1^ at 100 mA g^−1^(Figure 9i) and a retained capacity of 99.6% after 1000 cycles at 4 A g^−1^(Figure 9j). Cao et al.^[^ ^202^ ^]^ also demonstrated that the hypothesis of the Zn^2+^ (de)intercalation process in V_3_O_7_∙H_2_O cathode (Figure 9k) was consistent with the experimental results.

#### 3.5.4 V_5_O_12_ 6H_2_O

**Figure 10:**
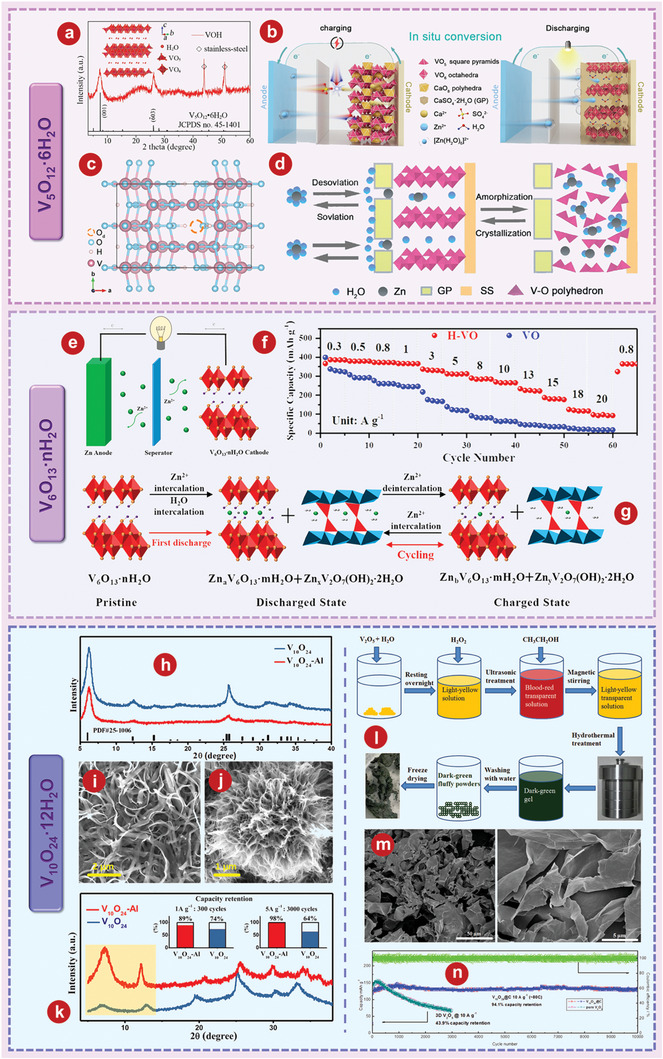
a) XRD pattern of the as‐prepared VOH cathode grown on stainless‐steel mesh. The inset schematically represents the layered structure of VOH. b) Schematic illustration of the crystal structure evolution from CVO to GP‐HVO_d_. c) Crystal structure of HVO_d_. d) Schematic diagram of zinc storage mechanism for GP‐HVO_d_. e) Schematic Illustration of an AZIB Based on V_6_O_13_ *n*H_2_O cathode and metallic Zn foil anode. f) Rate capabilities of V_6_O_13_ *n*H_2_O and V_6_O_13_ electrodes. g) Schematic illustration of Zn^2+^ and water co‐intercalation into V_6_O_13_ *n*H_2_O electrode during the initial discharge process and reversible Zn^2+^ (de)intercalation in the subsequent process. h) XRD patterns of V_10_O_24_ 12H_2_O and aluminum‐doped V_10_O_24_ 12H_2_O. SEM images of i) pure V_10_O_24_ 12H_2_O and j) Al‐doped V_10_O_24_ 12H_2_O. k) XRD patterns of V_10_O_24_ 12H_2_O and aluminum‐doped V_10_O_24_ 12H_2_O electrodes obtained at the fully charged state after long‐term cycles for 21 days (collected at 100 mA g^−1^ after 140 cycles). The inserted bar graph provides the comparison of capacity retention at different currents. l) Schematic illustration of fabricating 3D structures of V_10_O_24_ 12H_2_O nanosheets coated with carbon (V_10_O_24_@C). m) SEM images of V_10_O_24_@C. n) The batteries were tested in 3 m aqueous Zn(CF_3_SO_3_)_2_ electrolyte. a) Reproduced with permission.^[^ ^203^ ^]^ Copyright 2019, WILEY‐VCH. b–d) Reproduced with permission.^[^ ^204^ ^]^ Copyright 2022, WILEY‐VCH. e–g) Reproduced with permission.^[^ ^205^ ^]^ Copyright 2019, American Chemical Society. h–k) Reproduced with permission.^[^ ^208^ ^]^ Copyright 2019, American Chemical Society. l–n) Reproduced with permission.^[^ ^209^ ^]^ Copyright 2021, American Chemical Society.

Navajoite V*_5_* O_12_ 6H_2_O, consisting of bilayers (VO_6_ octahedron and VO_5_ square pyramid) stacked with stably bound water molecules, is one of the representatives of the layered hydrated vanadium oxide.^[^ ^59^ ^]^ Due to the characteristics of its structure, V*_5_* O_12_ 6H_2_O can provide a larger interlayer distance of about 1.18 nm, thus providing open channels for Zn^2+^ (de)intercalation. Zhang et al.^[^ ^203^ ^]^ developed a high‐performance cathode by uniformly placing the V_5_O_12_ 6H_2_O nanoribbon cathode on a stainless‐steel substrate using a simple electrodeposition technique. **Figure** **10**a shows the XRD pattern of the prepared V_5_O_12_ 6H_2_O sample. The discharge capacity of V_5_O_12_ 6H_2_O is 354.8 mAh g^−1^ (0.5 A g^−1^), the initial CE is up to 99.5%, the energy density is up to 194 Wh kg^−1^ (2100 W kg^−1^), and the capacity retention rate is up to 94% after 1000 cycles. Huang and coworkers^[^ ^204^ ^]^ made CaV_4_O_9_ cathode reconstruct as the oxygen‐deficient V_5_O_12‐x_ 6H_2_O coated by gypsum layers (GP‐HVO_d_) through the initial electrochemically charging (Figure 10b). The crystal structure of V_5_O_12‐x_ 6H_2_O (HVO_d_) is shown in. Figure 10c. Based on its structure, Huang and colleagues^[^ ^204^ ^]^ used DFT calculations to reveal the role of O_d_ in optimizing electronic properties. The overall Zn^2+^ storage mechanism of GP‐HVO_d_ was shown in Figure 10d. GP‐HVO_d_ exhibits good performance, with a high capacity of 402.5 mAh g^−1^ and excellent cycle stability of 99.7% capacity retention after 200 cycles at 0.2 A g^−1^.

#### 3.5.5 V_6_O_13_ *n*H_2_O

The intercalation of water in the interlayer expansion V_6_O_13_ *n*H_2_O is due to the strong intercalation of water molecules and lattice oxygen ions between monolayer and bilayer, forming hydroxyl radicals on each side. The expanded interlayer spacing due to the intercalation of water molecules provides good rate performance and cyclic stability for Zn^2+^ storage. In addition, the mixed valence of V^4+^ and V^5+^ within V_6_O_13_ and high conductivity also provide excellent conditions for Zn ion storage. Lai et al.^[^ ^205^ ^]^ prepared a highly reversible AZIB (Figure 10e) by employing V_6_O_13_ *n*H_2_O hollow micro‐flowers composed of ultra‐thin nanosheets as a cathode material. By increasing the current density from 0.3 to 10 A g^−1^, the V_6_O_13_ *n*H_2_O cathode shows excellent capacities of 386 and 270 mAh g^−1^, respectively, with only a 30% capacity loss, which is much better than V_6_O_13_ cathode. The storage mechanism of Zn^2+^ is shown in figure 10g. We can clearly see the reaction of the first (dis)charge of V_6_O_13_ *n*H_2_O cathode and then the steady (dis)charge.

#### 3.5.6 V_10_O_24_ 12H_2_O

V_10_O_24_ 12H_2_O can be understood as a kind of oxygen‐deficient V_2_O_5‐x_ *n*H_2_O compound and also a type of typical hybrid valence hydrated vanadium oxide where the molar ratio of the V^5+^/V^4+^ is 4.^[^ ^206^ ^]^ However, the current synthesis process of V_10_O_24_ 12H_2_O is relatively complex, time‐consuming, and less studied.^[^ ^207^ ^]^ Li et al.^[^ ^208^ ^]^ prepared an aluminum‐doped V_10_O_24_ 12H_2_O as a cathode material for AZIBs. As shown in Figure 10h, there is no obvious peak position shift in aluminum‐doped materials, which may‐ be due to the low doping degree. SEM images of V_10_O_24_ 12H_2_O and aluminum‐doped V_10_O_24_ 12H_2_O are shown in Figure 10i,j. Compared with the V_10_O_24_ 12H_2_O, the aluminum‐doped V_10_O_24_ 12H_2_O consists of denser and finer bands mixed together to produce a unified urchin‐like state. After 21 days of work and recharging to 1.6 V, the structure of aluminum‐doped V_10_O_24_ 12H_2_O is not completely destroyed (high‐intensity (002) peak), while the layer structure of V_10_O_24_ 12H_2_O is almost destroyed (broader and attenuated (002) and (004) peaks), shown in Figure 10k. Wu et al.^[^ ^209^ ^]^ synthesized V_10_O_24_ 12H_2_O nanosheets coated with carbon (V_10_O_24_@C) used as cathode materials for AZIBs (Figure 10l). It's observed that V_10_O_24_@C is a porous 3D structure consisting of a large number of intercalated curved nanosheets, similar to the reported 3D V_2_O_5_ network and 3D graphene networks (Figure 10m).^[^ ^210^, ^211^, ^212^, ^213^, ^214^, ^215^ ^]^ As shown in Figure 10n, the V_10_O_24_@C cathode delivers superior performance with a capacity retention of 94.1% after 10 000 cycles at a 10 A g^−1^.

## 4 Vanadates

Vanadates have abundant chemical valence vanadium and V—O polyhedron and are easy to deform. Generally, many vanadates are prepared by intercalating vanadate oxides with different kinds of cations. The intercalation of cation can increase vanadium oxide's internal spacing, thus effectively easing the capacity loss of vanadium oxides. In addition, the intercalation of cation has been shown to have a “pillar effect”, enhancing the layered structure and inhibiting “lattice respiration”, thereby enhancing the cycling stability.^[^ ^239^, ^240^ ^]^ So far, researchers have designed many different kinds of metal ions (including monovalent alkali metal cations Li+, Na^+^, K^+^, multivalent alkali metal cations Ca^2+^, Mg^2+^, transition metal cations Cu^2+^, Ag^+^, nonmetal cations NH_3_^+^, etc.) to intercalate between layers of vanadium oxides to use as cathode materials for AZIBs. In addition, as the important branch of vanadates, hydrated vanadates exhibit unique properties due to the intercalation of water molecules.

### 4.1 Monovalent Alkali Metal Cations

#### 4.1.1 The Intercalation of Li^+^

##### Li_x_V_3_O_8_

**Figure 11:**
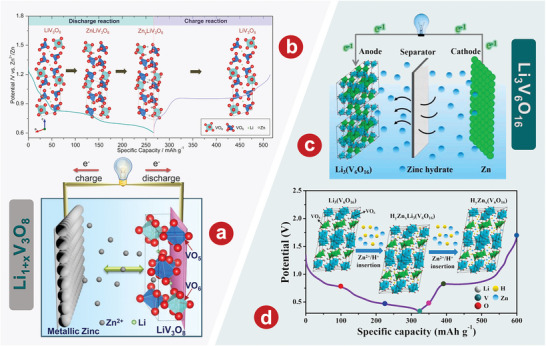
a) Schematic diagram of Zn–LiV_3_O_8_ battery. b) Schematic of the Zn‐intercalation mechanism in the present LiV_3_O_8_ cathode. c) Schematic diagram of Zn‐Li_3_V_6_O_16_ battery. d) Schematic diagram of the storage mechanism of Zn^2+^ in the Li_3_V_6_O_16_//Zn battery. (a, b) Reproduced with permission.^[^ ^241^ ^]^ Copyright 2017, American Chemical Society. c, d) Reproduced with permission.^[^ ^242^ ^]^ Copyright 2022, Elsevier.

Li_x_V_3_O_8_ consists of two edge‐shared VO_6_ and VO_5_ units via corner‐sharing, and the V_3_O_8_^−^ layers are connected by Li^+^ at the interstitial octahedral and tetrahedral sites. The stable layered structure of Li_1+x_V_3_O_8_, with high ion diffusion rate, can provide many vacancies for Zn^2+^ to occupy. In addition, the possibility of metal ion intercalation and the extensive charge balance characteristics of the vanadium redox couple (V^5+^/V^4+^/V^3+^) make the layered Li_1+x_V_3_O_8_ as a candidate cathode material of AZIBs. Alfaruqi and colleagues^[^ ^241^ ^]^ prepared layered‐type LiV_3_O_8_ as a promising cathode material for AZIBs with high capacity. The schematic diagram of the assembled AZIB based on layered‐type LiV_3_O_8_ cathode is shown in **Figure** **11**a. At 133 mA g^−1^, the LiV_3_O_8_ cathode has a high specific capacity of 172 mAh g^−1^ after 65 cycles, as well as the CE is ≈100%. The electrochemical insertion of Zn^2+^ in LiV_3_O_8_ is mainly described by a storage mechanism that shows that zinc transitions in LiV_3_O_8_ in a single‐phase step by step and transitions to Zn_y_LiV_3_O_8_ phase (Figure 11b).

##### Li_3_V_6_O_16_

**Figure 12:**
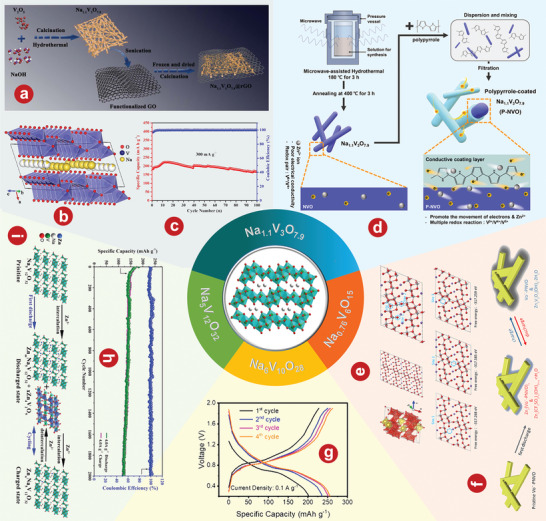
a) Illustration of synthesis procedure of pilotaxitic Na_1.1_V_3_O_7.9_ nanoribbons/rGO. b) The structural framework of Na_1.1_V_3_O_7.9_. c) The cycling performance of Na_1.1_V_3_O_7.9_@rGO for AZIBs at 300 mA g^−1^. d) Schematic illustration of the synthesis of Na_1.1_V_3_O_7.9_ and Na_1.1_V_3_O_7.9_/PPy cathode for AZIBs. e) The crystal structure of NVO, the possible sites for oxygen vacancies in Na_0.76_V_6_O_15_ nanobelts (left), and corresponding calculated free energy. The smallest free energy on Site 2 suggests O_b_ site is the location of oxygen vacancies in Vo¨‐PNVO (right). f) Illustration of the energy storage mechanism of Vo¨‐PNVO. g) Galvanostatic charge‐discharge plots of the first four cycles at a current density of 0.1 A g^−1^ of Na_6_V_10_O_28_. h) Long‐term cycling performance at 4 A g^−1^ of Na_5_V_12_O_32_. i) Schematic illustration of Zn^2+^ (de)intercalation process of Na_5_V_12_O_32_ during cycling. a–c) Reproduced with permission.^[^ ^246^ ^]^ Copyright 2018, Elsevier. d) Reproduced with permission.^[^ ^247^ ^]^ Copyright 2022, Elsevier. e, f) Reproduced with permission.^[^ ^248^ ^]^ Copyright 2021, Elsevier. g) Reproduced with permission.^[^ ^250^ ^]^ Copyright 2022, Elsevier. h, i) Reproduced with permission.^[^ ^254^ ^]^ Copyright 2018, WILEY‐VCH.

Interestingly, the Li_3_V_6_O_16_ and Li_x_V_3_O_8_ are homogeneous and multi‐image variants with the same hierarchical structure and similar performance. Ran et al.^[^ ^242^ ^]^ prepared the Li_3_V_6_O_16_ as cathode material of AZIBs with high storage capacity via a one‐step molten salt method. The schematic diagram of the AZIB based on Li_3_V_6_O_16_ cathode is shown in **Figure** **12**c. The Li_3_V_6_O_16_ shows superior electrochemical performance with 350 mAh g^−1^ at 0.1 A g^−1^. As shown in Figure 12d, Ran et al.^[^ ^242^ ^]^ also proved that the electrochemical mechanism of the Li_3_V_6_O_16_ cathode is Zn^2+^ and H^+^ co‐intercalation.

#### 4.1.2 The Intercalation of Na^+^

##### Na_1.1_V_3_O_7.9_

Na_1.1_V_3_O_7.9_ shows a layered structure that consists of alternating VO_6_ octahedron and VO_4_ tetrahedron, connected by O atoms with shared corners. The sodium ions occupy two separate positions. One is a fully occupied octahedral site and the other is a partially occupied tetrahedral site. In this layered structure, sodium or zinc ions have an open and stable interstitial channel during de(intercalation).^[^ ^243^, ^244^, ^245^ ^]^ Cai and coworkers^[^ ^246^ ^]^ first synthesized the ignition Na_1.1_V_3_O_7.9_ nanoribbons/graphene composites, by a simple hydrothermal method, followed by freeze‐drying (Figure 12a). The schematic diagram of the structural framework of Na_1.1_V_3_O_7.9_ is shown in Figure 12b. When used as a cathode material for AZIBs, the composite can reach a maximum value of 223 mA h g^−1^ after 14 cycles at 300 mA g^−1^ (Figure 12c) and has good cycle stability, indicating that Na_1.1_V_3_O_7.9_ is a good candidate material for the cathode of AZIBs. Islam et al.^[^ ^247^ ^]^ used PPy coating to enhance the electrochemical performance of Na_1.1_V_3_O_7.9_. Na_1.1_V_3_O_7.9_ coated with PPy (P‐NVO) was synthesized by microwave‐assisted hydrothermal method followed by calcined, and finally mixed with PPy in dry ethanol (Figure 12d). The highly conductive polypyridine surface coating is of great significance to improve the conductivity of Zn^2+^ and the kinetics of Zn^2+^ diffusion, which can enable the Na_1.1_V_3_O_7.9_ cathode to perform V^3+^/V^4+^/V^5+^ multiple redox reactions in AZIBs. Even at high current densities of 6000 mA g^−1^, the P‐NVO cathode shows unprecedented cycle stability over 1100 cycles without capacity loss.

##### The Intercalation of Organic Molecules in Na_0.76_V_6_O_15_

Na_0.76_V_6_O_15_ have a 3D rigid tunnel structure, can effectively alleviate the collapse of the structure, and the Zn^2+^ is reversible de(intercalation). The tunneled Na_0.76_V_6_O_15_ consists of VO_6_ octahedron, which is connected to each other by V—O_b_ bonds and to the layer by V—O_c_ bonds. Based on crystal symmetry, there are three sites in tunnel Na_0.76_V_6_O_15_: O_v_ (Site 1), O_b_ (Site 2), and O_c_ (Site 3), as shown in Figure 12e. Bi et al.^[^ ^248^ ^]^ investigated the application of poly(3,4‐ethylenedioxythiophene) (PEDOT) coatings in AZIBs by in situ polymerization to introduce oxygen vacancies in Na_0.76_V_6_O_15_ nanoribbons (Vo¨‐PNVO). The rapid reversible diffusion and intercalation of Zn^2+^ are achieved by introducing “oxygen vacancies”, which enlarge the interplanar space and weakened the electrostatic interaction. At 50 mA g^−1^, Vo¨‐PNVO cathode exhibits an improved specific capacity of 355 mA h g^−1^. According to the ex situ XRD, XPS, and SEM results, the energy storage mechanism of Vo¨‐PNVO is shown in Figure 12f.

##### Na_6_V_10_O_28_

PVOs‐typed Na_6_V_10_O_28_ is composed of 1 V_10_O_28_^6−^, 2 Na(H_2_O)_4_^+^, 2 Na_2_ (H_2_O)_3_^2+^ and 4 additional water molecules, respectively. The V_10_O_28_^6−^ is composed of 10 VO_6_ polyhedron, among which 6 VO_6_ octahedrons are arranged in a 2 × 3 rectangular array by shared edges and the remaining 4 VO_6_ octahedrons are distributed on the upper and lower sides through shared sloping edges.^[^ ^249^ ^]^ In accordance with the above structure, discrete components of polyanion, and extended the absence of the crystal structure, making the storage of Zn^2+^ easier to (de)intercalate. In addition, the storage mechanism for this process is (de)intercalate of Zn^2+^ between V_10_O_28_^6−^ rather than entry into the crystal structure. Zhou et al.^[^ ^250^ ^]^ first applied PVOs‐typed Na_6_V_10_O_28_ as a cathode material for AZIB and verified its feasibility. The high stability of the V_10_O_28_^6−^ cluster allows the material to support reversible intercalation of Zn^2+^. Thus, the Na_6_V_10_O_28_ cathode provides a high capacity of 279.5 mAh g^−1^ (Figure 12g) and excellent cycle performance.

##### Na_5_V_12_O_32_

Among the sodium vanadates, Na_5_V_12_O_32_ has many oxidation states, high specific capacity, good structural stability, low cost, and safety, which is considered as a promising cathode material.^[^ ^251^ ^]^ The crystal structure of Na_5_V_12_O_32_ is a layered structure composed of V_3_O_8_ polyhedral layers, and sodium ions are mainly located in the octahedral position between the layers. The sodium ions located in the octahedral position act as pillar cations to stabilize the structure, making this structure very favorable.^[^ ^252^, ^253^ ^]^ Guo et al.^[^ ^254^ ^]^ purposefully selected and constructed three sodium vanadate nanoribbons with typical NaV_3_O_8_ layered structures (Na_5_V_12_O_32_ HNaV_6_O_16_ 4H_2_O and Na_0.76_V_6_O_15_), and applied them in AZIBs. Na_5_V_12_O_32_ has a higher capacity than Na_0.76_V_6_O_15_, with a long‐term cycle performance of up to 2000 cycles at 4.0A g^−1^ despite capacity reduction (Figure 12h). Based on ex situ transmission electron microscopy (TEM)images and ex situ X‐ray photoelectron spectroscopy (XPS), Guo et al.^[^ ^254^ ^]^ demonstrated the Zn^2+^ de(intercalation) process during Na_5_V_12_O_32_ cycling (Figure 12i).

#### 4.1.3 The Co‐Intercalation of Na^+^ and H_2_O

##### Na_x_V_2_O_5_ *n*H_2_O

**Figure 13:**
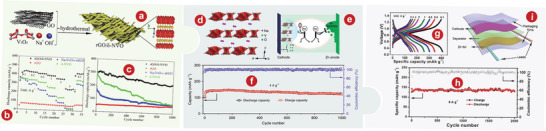
a) The synthesis procedure and crystallographic structure of *δ*‐type Na_x_V_2_O_5_ *n*H_2_O. b) Rate performances of four samples at current densities from 0.1 to 5 A g^−1^. c) Cycling performances at 2 A g^−1^. d) Crystal structure of NaV_3_O_8_ 1.5H_2_O nanobelts, Na^+^ exists in the form of hydrated ion. e) Schematic diagram: Na2SO4 additive suppresses the dissolution of NaV_3_O_8_ 1.5H_2_O nanobelts and the formation of zinc dendrites. f) Long‐term cycle life of Zn/NaV_3_O_8_ 1.5H_2_O batteries in 1 m ZnSO_4_ electrolyte with 1 m Na_2_SO_4_ additive at 4 A g^−1^. g) Charge/discharge curves of rGO/NVO‐70% composite film‐based AZIBs at different current densities. h) Long cycle life of rGO/NaV_3_O_8_ 1.5H_2_O –70% composite film‐based AZIBs at 5 A g^−1^. i) Schematic diagram of flexible soft‐packaged AZIBs. a–c) Reproduced with permission.^[^ ^256^ ^]^ Copyright 2019, Elsevier. d–f) Reproduced with permission.^[^ ^62^ ^]^ Copyright 2018, Nature. g, h) Reproduced with permission.^[^ ^259^ ^]^ Copyright 2019, Springer.

In crystal structure of *δ*‐type Na_x_V_2_O_5_ *n*H_2_O, interlayer H_2_O molecules and Na^+^ can act as pillars to stabilize the V_2_O_5_ layer and shield electrostatic interactions between cations intercalation during (de)charging. However, *δ*‐type Na_x_V_2_O_5_ *n*H_2_O has low conductivity compared with other vanadium‐based compounds, which is not conducive to electrochemical energy storage. Graphene is proving to be an excellent functional material as the scaffold to solve the problem of low conductivity of vanadium‐based cathode materials.^[^ ^255^ ^]^ Zhou et al.^[^ ^256^ ^]^ first prepared a *δ*‐type Na_x_V_2_O_5_ *n*H_2_O hybrid with rGO (**Figure** **13**a), which showed better electrochemical performance than Na_2_V_6_O_16_ *n*H_2_O under the same conditions. The prepared *δ*‐type Na_x_V_2_O_5_ *n*H_2_O hybrid with rGO has a good reversible capacity of 433.5 mAh g^−1^ at 0.1 A g^−1^, an excellent rate capability of 244.1 mAh g^−1^ at 5 A g^−1^ (Figure 13b). In addition, the *δ*‐type Na_x_V_2_O_5_ *n*H_2_O hybrid with rGO also shows superior cycling stability of 70.5% more than 1000 cycles (Figure 13c).

##### NaV_3_O_8_ 1.5H_2_O

In the crystal structure of NaV_3_O_8_ 1.5H_2_O (Figure 13d), hydrated Na^+^ are positioned between layers V_3_O_8_ as pillars to stabilize the layered structure composed of a VO_5_ tetragonal bipyramid and a VO_6_ octahedron. Except for Na^+^, which is easy to intercalate, the interlayer distance of NaV_3_O_8_ (7.08 Å) is large enough to intercalate Zn^2+^ (0.74 Å), and H^+^ can stably exist between layers of V_3_O_8_.^[^ ^257^, ^258^ ^]^ Wang et al.^[^ ^62^ ^]^ developed a highly reversible zinc vanadate/sodium vanadate system with NaV_3_O_8_ 1.5H_2_O nanoribbon as the positive electrode and zinc sulfate aqueous solution of sodium sulfate additive as the electrolyte. Na_2_SO_4_ additive inhibited the dissolution of NaV_3_O_8_ 1.5H_2_O nanoribbons and the formation of zinc dendrites (Figure 13e). The reversible capacity of the zinc/sodium vanadate hydrate cell is 380 mAh g^−1^, and the capacity retention rate is up to 82% after 1000 cycles (Figure 13f). On the basis of the above research, Wan et al.^[^ ^259^ ^]^ prepared an independent rGo/NaV_3_O_8_ 1.5H_2_O nanocomposite film by vacuum filtration method to solve the problem of low conductivity of NaV_3_O_8_ 1.5H_2_O. The rGo/NaV_3_O_8_ 1.5H_2_O composite films have a unique interconnected multilayer structure and many pores, so they have high electronic conductivity and abundant ion transport channels, showing a high capacity of 410 mAh g^−1^ at 0.1A g^−1^ (Figure 13g) and superior cycling stability with 94% after 2000 cycles (Figure 13h). In addition, Wan et al.^[^ ^259^ ^]^ also made based on flexible soft rGo/NaV_3_O_8_ 1.5H_2_O composite film packaging AZIBs to prove the concept (Figure 13i).

#### 4.1.4 The Intercalation of K^+^

##### K_x_V_2_O_5_

**Figure 14:**
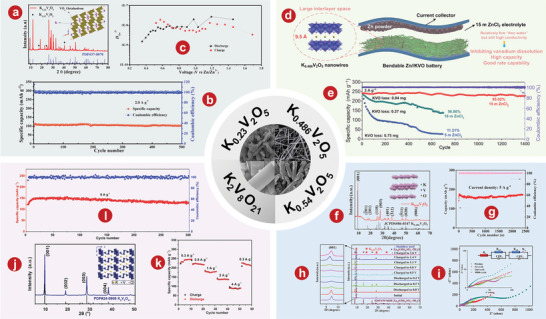
a) XRD pattern and the crystal structure of K_0.23_V_2_O_5_. b) Cyclic performance of the K_0.23_V_2_O_5_ electrode measured at 2.0 A g^−1^. c) Diffusion coefficients of Zn^2+^ in the K_0.23_V_2_O_5_ during charge/discharge process. d) Schematic illustration of bendable Zn// K_0.486_V_2_O_5_ battery with regulating electrolyte concentration. e) Cycle stability comparison in 5, 10, and 15 m ZnCl_2_ electrolytes at 3 A g^−1^. f) XRD pattern and the crystal structure of K_0.54_V_2_O_5_. g) Long cycle performance of K_0.54_V_2_O_5_ at 5.0 A g^−1^. h) XRD patterns of K_0.54_V_2_O_5_ during the initial discharge and charge cycle. i) EIS spectrum of the K_0.54_V_2_O_5_ electrode before and after cycling. j) XRD pattern and the crystal structure of K_2_V_8_O_21_. k) Rate capability of the Zn//K_2_V_8_O_21_ battery. l) Long‐term cycling performance at 6 A g^−1^ of the Zn//K_2_V_8_O_21_ battery. a–c) Reproduced with permission.^[^ ^266^ ^]^ Copyright 2019, Elsevier. d, e) Reproduced with permission.^[^ ^267^ ^]^ Copyright 2021, Springer. f–i) Reproduced with permission.^[^ ^268^ ^]^ Copyright 2022, American Chemical Society. j–l) Reproduced with permission.^[^ ^269^ ^]^ Copyright 2018, Elsevier

In the structure of K_x_V_2_O_5_, potassium ions are intercalated into the gap between the VO_6_ octahedron,^[^ ^260^, ^261^ ^]^ which increases the d spacing during Zn^2+^ (de)intercalation and reduces the charge polarization effect, thus enhancing the structural stability and electrochemical performance.^[^ ^262^ ^]^ In addition, the atomic radius of K^+^ is larger than that of Li^+^, Na^+^, and Zn^2+^, which can become a stronger “pillar” between vanadium and oxygen layers,^[^ ^263^ ^]^ thus enhancing the structural stability of the material.^[^ ^264^, ^265^ ^]^ Zhang et al.^[^ ^266^ ^]^ synthesized K_0.23_V_2_O_5_ with tunnel structure by hydrothermal method, which was first used as a cathode material for AZIBs. The XRD pattern and the crystal structure of K_0.23_V_2_O_5_ are shown in **Figure** **14**a. The K_0.23_V_2_O_5_ cathode has excellent structural stability and high‐capacity retention of 92.8% after 500 cycles at 2.0 A g^−1^ (Figure 14b). Furthermore, as shown in Figure 14c, the ion diffusion rate is up to 1.88× 10^−9^–2.6×10^−8^ cm^2^ S^−1^, which is much higher than most other cathode materials of AZIBs. Li et al.^[^ ^267^ ^]^ further developed a AZIB with high performance consisting of a layered K_0.486_V_2_O_5_ nanowire cathode with large interlayer spacing and a Zn powder anode (Figure 14d). When the optimum concentration of ZnCl_2_ electrolyte is 15 m, the cycle stability of K_0.486_V_2_O_5_ is the best, and the capacity retention rate is 95.02% after 1400 cycles (Figure 14e). The work of Li et al.^[^ ^267^ ^]^ illustrates the feasibility of using moderately concentrated electrolytes to solve the stability problem of aqueous‐soluble electrode materials. In addition, Wu et al.^[^ ^268^ ^]^ prepared a nanorod‐shaped K_0.54_V_2_O_5_, which is a promising cathode material for AZIBs. The crystal structure information of the K_0.54_V_2_O_5_ is shown in the XRD pattern in Figure 14f. The K_0.54_V_2_O_5_ cathode delivers superior electrochemical performance with a capacity retention rate of 97% (176 mA h g^−1^) after 2400 cycles at 5 A g^−1^, as shown in Figure 14g. In addition, as shown in Figure 14h, the discharge diffraction peak of the 50th sample is similar to that of the 100th sample, which also proves its excellent performance and cyclic stability. As shown in Figure 14i, the curve radius changed a little after the second cycle and the 100th cycle, which indicates that this material structure tends to be stable during the process of Zn^2+^ (de)intercalation.

##### K_2_V_8_O_21_

The tunnel structure of K_2_V_8_O_21_ consists of a vanadate framework consisting of a VO_6_ octahedron and a VO_5_ pyramid forming [V_8_O_21_]^2−^ units along the b‐axis, while K^+^ fill the tunnel as “pillars” to stabilize the structure. Tang et al.^[^ ^269^ ^]^ successfully synthesized K_2_V_8_O_21_ nanobelts, K_0.25_V_2_O_5_ nanobelts, KV_3_O_8_ nanobelts, and K_2_V_6_O_16_ 1.57H_2_O nanobelts, and applied them to AZIBs cathode for the first time. Figure 14j shows the XRD pattern of K_2_V_8_O_21_. Of the four potassium vanadates, K_2_V_8_O_21_ cathode showed the best zinc storage performance due to the stable tunnel structure, with a capacity of 247 mAh g^−1^ at 0.3 Ag^−1^ (Figure 14k), and superior capacity retention of 83% after 300 cycles even at a high current density of 6 A g^−1^ (Figure 14l).

##### K_10_[V^IV^_16_V^V^_18_O_82_]

**Figure 15:**
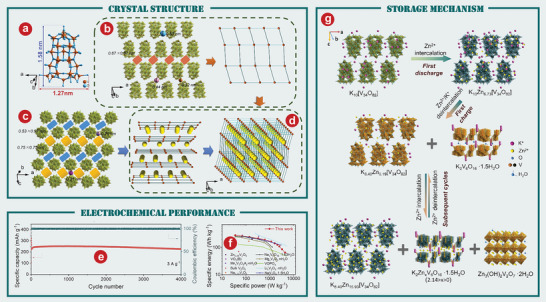
a) The molecular structure of the [V^IV^_16_V^V^_18_O_82_]^10−^ polyoxoanion in K_10_[V^IV^_16_V^V^_18_O_82_] 20H_2_O. Vanadium: orange, oxygen: blue. b) View of the staggered packing of [V^IV^_16_V^V^_18_O_82_]^10−^ along the (0 1 –1) face and its topological view in K_10_[V^IV^_16_V^V^_18_O_82_] 20H_2_O. c) The (0 1 1) face of [V^IV^_16_V^V^_18_O_82_]^10−^ arrangement and their Zn^2+^ migration channels highlighted by the rectangles (blue and yellow) and the balls (purple and pink). d) Topologic images of hierarchical interconnected channels based on (0 1 –1) face stacked along b axis. The continuous yellow balls highlight the migration pathways. e) Long‐cycle performance of the battery equipped with K_10_[V^IV^_16_V^V^_18_O_82_] cathode at 3 A g^−1^. f) Ragone plots: comparison of energy and power densities of the Zn/K_10_[V^IV^_16_V^V^_18_O_82_] battery with ZIBs based on other reported cathodes. g) Schematic illustration of Zn^2+^ intercalation/deintercalation process of the K_10_[V^IV^_16_V^V^_18_O_82_] cathode during cycling. Reproduced with permission.^[^ ^270^ ^]^ Copyright 2020, Elsevier.

The nanosized ellipsoid‐shaped [V^IV^_16_V^V^_18_O_82_]^10−^ polyoxoanion is constructed from a central V_4_O_8_ cube and 30 VO_5_ square pyramids through 26 µ_2_‐and 4 µ_3_‐oxygen atoms. In addition, the 3D packing aligment of [V^IV^_16_V^V^_18_O_82_]^10−^ polyoxoanions and K^+^ ions result in available multidimensional interconnected Zn^2+^ (de)intercalation channels. Yang et al.^[^ ^270^ ^]^ first prepared the hybrid valence K_10_[V^IV^_16_V^V^_18_O_82_] cluster with nanoscale as cathode material of AZIBs for Zn^2+^ energy storage. The K_10_[V^IV^_16_V^V^_18_O_82_] was obtained by drying K_10_[V^IV^_16_V^V^_18_O_82_] 20H_2_O in a nitrogen atmosphere at 200 °C for three hours. The molecular structure of the [V^IV^_16_V^V^_18_O_82_]^10−^ polyoxoanion in K_10_[V^IV^_16_V^V^_18_O_82_] 20H_2_O is shown in **Figure** **15**a. Figure 15b–d gives a deeper description of the structure of K_10_[V^IV^_16_V^V^_18_O_82_] 20H_2_O. The K_10_[V^IV^_16_V^V^_18_O_82_] cluster constructed hierarchical interconnected migration channels in different spatial dimensions and improved the Zn^2+^ transport capacity. The prepared Zn//K_10_[V^IV^_16_V^V^_18_O_82_] battery has good cycle stability with a capacity retention rate of 93% over 4000 cycles at 3 A g^−1^ (Figure 15e), and good energy of 285 Wh kg^−1^ and power density of 4.5 kW kg^−1^ (Figure 15f). According to the previous research,^[^ ^269^ ^]^ Yang et al.^[^ ^270^ ^]^ came up with a logical storage mechanism for the K_10_[V^IV^_16_V^V^_18_O_82_] cathode during cycling, as shown in Figure 15g.

#### 4.1.5 The Co‐Intercalation of K^+^ and H_2_O

##### KV_3_O_8_ 0.75H_2_O

**Figure 16:**
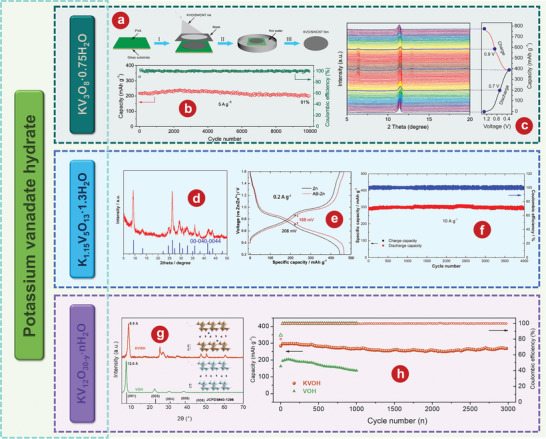
a) Schematic diagram of KV_3_O_8_ 0.75H_2_O/SWCNTs films. b) Long‐term cycle life at a 5 A g^−1^. c) In situ XRD patterns of KV_3_O_8_ 0.75H_2_O/SWCNTs electrode during (dis)charge process. d) XRD patterns of K_1.15_V_5_O_13_ 1.3H_2_O. e) Typical charge‐discharge profiles for the Zn//K_1.15_V_5_O_13_ 1.3H_2_O and AB‐Zn//K_1.15_V_5_O_13_ 1.3H_2_O in 3 m Zn(OTF_)2_ AE at 0.2 A g^−1^. f) Cycling performance of K_1.15_V_5_O_13_ 1.3H_2_O at 10 A g^−1^. g) XRD patterns of samples (inset are possible schematic frameworks). h) Long‐term cycle life of samples tested at 5 A g^−1^. a–c) Reproduced with permission.^[^ ^271^ ^]^ Copyright 2020, American Chemical Society. d–f) Reproduced with permission.^[^ ^272^ ^]^ Copyright 2021, American Chemical Society. g, h) Reproduced with permission.^[^ ^274^ ^]^ Copyright 2020, Elsevier.

Due to less structural water, KV_3_O_8_ 0.75H_2_O exhibits better structural stability than NaV_3_O_8_ 1.5H_2_O in aqueous solution.^[^ ^62^ ^]^ The KV_3_O_8_ 0.75H_2_O cathode uses V_3_O_8_ as the skeleton layer, which has a high capacity due to the redox effect of V^3+^/V^5+^ pairs and large layer spacing. Wan et al.^[^ ^271^ ^]^ prepared KV_3_O_8_ 0.75H_2_O and further integrated it into SWCNTs (SW = single wall) network by a spray printing strategy to achieve independent KV_3_O_8_ 0.75H_2_O/SWCNTs composite films (**Figure** **16**a). The KV_3_O_8_ 0.75H_2_O cathode delivers a high capacity of 379 mAh g^−1^, superior rate capability, as well as a high capacity of 91% to maintain stable cycling performance after 10 000 cycles at 5 A g^−1^ (Figure 16b). As shown in Figure 16c, Wan et al.^[^ ^271^ ^]^ also studied the structural evolution of KV_3_O_8_ 0.75H_2_O during (dis)charging by using in situ XRD.

##### K_1.15_V_5_O_13_ 1.3H_2_O

On the basis of the work proposed by Wan et al. to composite KV_3_O_8_ 0.75H_2_O with SWCNTs, Qiu and colleagues^[^ ^272^ ^]^ reported potassium vanadate nanoribbons as a promising cathode for AZIBs. By XRD pattern (Figure 16d) and energy dispersive spectrometer (EDS) of sample, the molecular formula of the sample is K_1.15_V_5_O_13_ 1.3H_2_O. The K_1.15_V_5_O_13_ 1.3H_2_O cathode along with acetylene black enhanced zinc foil (AB‐Zn) has a high discharge capacity of 461 mAh g^−1^ at 0.2 A g^−1^ (Figure 16e) and a capacity retention rate of 96.2% in 4000 cycles at 10 A g^−1^ (Figure 16f), which is expected to provide clues in the pursuit of energy storage devices with superior performance.

##### KV_12_O_30‐y_ *n*H_2_O

In the structure of KV_12_O_30‐y_ *n*H_2_O, there are more low‐price V^4+^ and more oxygen vacancies in the structure after the addition of K^+^. This phenomenon can also be observed in the previously published insert of hydrated Mn^2+^ into vanadate.^[^ ^273^ ^]^ Tian et al.^[^ ^274^ ^]^ prepared the structurally unique KV_12_O_30‐y_ *n*H_2_O by intercalating K^+^ into V_2_O_5_ *n*H_2_O via a structural engineering method. As shown in Figure 16g, V_2_O_5_ *n*H_2_O exhibits a typical bilayer structure similar to V_2_O_5_ 1.6H_2_O. For KV_12_O_30‐y_ *n*H_2_O, it exhibits a unique XRD pattern, which is different from the K^+^ integration structure reported in the literature.^[^ ^269^, ^275^ ^]^ KV_12_O_30‐y_ *n*H_2_O cathode delivers superior long‐term cycle life over 3000 cycles with 92% capacity retention at 5 A g^−1^ (Figure 16h), high energy density of 308 Wh kg^−1^, and power density of 7502 W kg^−1^, and enhanced energy efficiency.

### 4.2 Multivalent Alkali Metal Cations

#### 4.2.1 The Co‐Intercalation of Mg^2+^ and H_2_O

##### Mg_0.19_V_2_O_5_ 0.99H_2_O

**Figure 17:**
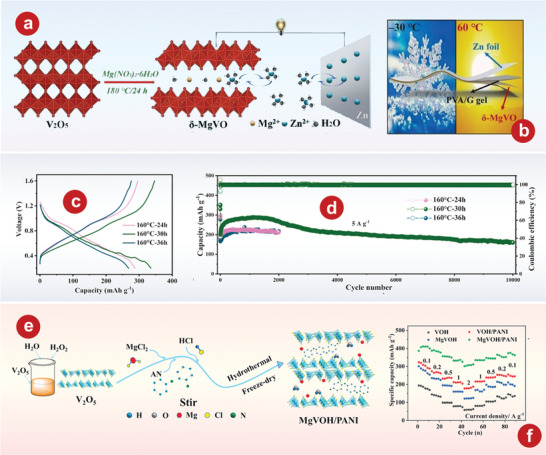
a) Schematic diagram showing the formation process of Mg_0.19_V_2_O_5_ 0.99H_2_O and the working principal of Zn//Mg_0.19_V_2_O_5_ 0.99H_2_O battery. b) Schematic diagram of the thin‐film PVA/G Zn//Mg_0.19_V_2_O_5_ 0.99H_2_O battery. c) Cycling performance at 0.1 A g^−1^. d) Long cycling performance at 5 A g^−1^. e) Schematic diagram of the synthesis of ternary material MgVOH/PANI. f) Rate capability of samples. a, b): Reproduced with permission.^[^ ^284^ ^]^ Copyright 2020, The Royal Society of Chemistry. c, d) Reproduced with permission.^[^ ^289^ ^]^ Copyright 2022, Elsevier. e, f) Reproduced with permission.^[^ ^290^ ^]^ Copyright 2022, The Royal Society of Chemistry.

The H_2_O molecules and Mg^2+^ between the layers can act as pillars to stabilize the V_2_O_5_ layer during the (dis)charging process.^[^ ^276^, ^277^, ^278^ ^]^ In addition, interlayer H_2_O molecules can expand the layer spacing and weaken strong electrostatic interactions, thus providing the benefit of the “lubrication effect” revealed by Mai and Liang et al.^[^ ^61^, ^279^ ^]^ Currently, the mass loading of active substances used in almost all scientific reports is usually much lower than commercial levels.^[^ ^280^, ^281^, ^282^, ^283^ ^]^ To solve this problem, Zhou and colleagues^[^ ^284^ ^]^ prepared a commercial‐level Mg_0.19_V_2_O_5_ 0.99H_2_O cathode with a mass load of 10 mg cm^−2^, which has a large interlayer spacing of 13.4 Å, and applied it to AZIBs (**Figure** **17**a). In addition, the Mg_0.19_V_2_O_5_ 0.99H_2_O cathode is assembled in combination with the PVA/glycerol gel electrolyte to form a quasi‐solid battery (Figure 17b), which shows high ionic conductivity over a wide temperature range, such as 10.7 mS cm^−1^ at −30 °C, and good compatibility with the zinc foil anode. Because of this, quasi solid‐state battery shows excellent performance at −30 to 60 °C.

##### Mg_0.2_V_2_O_5_ *n*H_2_O

MXenes (M_n+1_X_n_T_x_, n = 1, 2, 3), where M is transition metal (e.g., Ti, Nb, V, Mo, etc.), X is N and/or C, T_x_ is surface group (e.g., ‐F, ‐OH, = O, etc.), as 2D layered inorganic compounds with good conductivity, excellent hydrophilicity, nested structure, high specific surface area, and abundant active sites, have become hot materials in the field of electrochemical storage.^[^ ^285^, ^286^, ^287^, ^288^ ^]^ In addition, metal vanadate can be prepared by derivation of MXenes, followed by intercalation of metal ions. Guan et al.^[^ ^289^ ^]^ designed and prepared V_2_O_5_ *n*H_2_O nanoribbons with Mg^2+^ pre‐intercalation (Mg_0.2_V_2_O_5_ *n*H_2_O) derived from conductive V_4_C_3_ MXenes as the cathode of AZIBs, exhibiting a high reversible capacity of 346 mAh g^−1^ at 0.1 A g^−1^ (Figure 17c) and a capacity retention rate of 83.7% after 10 000 cycles at 5 A g^−1^ (Figure 17d).

#### 4.2.2 The Co‐Intercalation of Mg^2+^ and Polyaniline in Hydrated V_2_O_5_

##### Mg_0.1_V_2_O_5_ *n*H_2_O/PANI

Feng et al.^[^ ^290^ ^]^ used the “co‐intercalation mechanism” to simultaneously insert Mg^2+^ and PANI into the hydrated V_2_O_5_ layer by a one‐step hydrothermal method (Figure 17e). Mg^2+^ and PANI can expand the hydrated V_2_O_5_ layer spacing to 14.2 Å like pillars, which greatly reduces the coulomb interaction between Zn^2+^ and V_2_O_5_, thus speeding up the diffusion rate of Zn^2+^ and enhancing the storage performance of Zn^2+^.^[^ ^88^, ^291^ ^]^ In addition, PANI can also store Zn^2+^ as a guest, and Mg^2+^ can improve the conductivity and stability of the hydrated V_2_O_5_. The specific capacity of Mg_0.1_V_2_O_5_ *n*H_2_O/PANI can reach 412 mAh g^−1^ at 0.1 A g^−1^ (Figure 17f), and the capacity retention rate can reach 98% after 1000 cycles.

#### 4.2.3 The Co‐Intercalation of Ca^2+^ and H_2_O

##### Ca_0.04_V_2_O_5_ 1.74H_2_O

Ca_0.04_V_2_O_5_ 1.74H_2_O has a similar structure to hydrated vanadium pentoxide. The lattice spacing of (001) plane for Ca_0.04_V_2_O_5_ 1.74H_2_O is 12.48 Å, which is larger than the V_2_O_5_ *n*H_2_O and the previously reported hydrated vanadium pentoxide (11.5 Å),^[^ ^292^ ^]^ indicating that the insertion of a small amount of Ca^2+^ plays a key role in widening the lattice spacing.^[^ ^293^ ^]^ Du et al.^[^ ^294^ ^]^ prepared a small amount of Ca^2+^ pre‐intercalated V_2_O_5_ by hydrothermal method. The stable chemical bond energy between Ca and O atoms in VO_x_ is greater than that of Zn—O,^[^ ^295^ ^]^ which provides a fixed effect for the robust Ca_0.04_V_2_O_5_ 1.74H_2_O structure, enabling reversible Zn^2+^ intercalation and fast ion transport. The Ca_0.04_V_2_O_5_ 1.74H_2_O cathode has a high specific capacity of 400 mAh g^−1^ at 0.05A g^−1^ and a capacity retention rate of 100% at 10 A g^−1^ after 3000 cycles.

##### CaV_6_O_16_ 3H_2_O

CaV_6_O_16_ 3H_2_O is a typical vanadium‐bronze mineral, in which Ca^2+^ is in the interlayer space, coordinated by oxygens from the two vanadium slabs, facing each other, and by oxygens belonging to bound water molecules. Thus, the Ca^2+^ may act as pillaring agents to stabilize the structure of vanadium oxide, just as alkali metal ions do. Compared with the alkali vanadium bronze with molecular formulas M_x_V_2_O_5_, MV_3_O_8_, MV_6_O_15_, and M_2_V_6_O_16_ (M = Li, Na, or K), CaV_6_O_16_ 3H_2_O has a larger interlayer distance, which is conducive to shuttle ion intercalation. Liu and co‐workers^[^ ^296^ ^]^ prepared CaV_6_O_16_ 3H_2_O through a highly efficient and fast microwave reaction, and used it as a cathode material for AZIBs. Liu and co‐workers^[^ ^296^ ^]^ also demonstrated the reversibility of the process of Zn^2+^ (de)intercalation and the high structural stability of CaV_6_O_16_ 3H_2_O by ex situ XRD measurement.

#### 4.2.4 The Co‐Intercalation of Al^3+^ and H_2_O

##### H_11_Al_2_V_6_O_23.2_

It is possible to find V‐based cathode materials with zero‐strain properties, crystal plane “soft bond” ion channels with large interlayer spacing, and stable structures to improve easy Zn^2+^ (de)intercalation. Wei et al.^[^ ^47^ ^]^ prepared H_11_Al_2_V_6_O_23.2_ microspheres with large interlayer distances as cathode materials for AZIBs. The lattice structure remains well maintained even after 1000 cycles, and H_11_Al_2_V_6_O_23.2_ cathode has excellent reversibility, maintaining 88.6% capacity after 7000 cycles.

### 4.3 Transition Metal Cations

#### 4.3.1 The Intercalation of Ag^+^

##### *β*‐AgVO_3_

**Figure 18:**
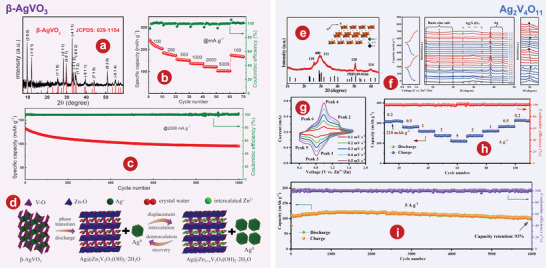
a) XRD pattern of *β*‐AgVO_3_. b) Rate performance of *β*‐AgVO_3_. c) The long‐term cycle stability at 2000 mA g^−1^ of the electrode. d) Schematic illustration of Zn^2+^ energy storage mechanism of the *β*‐AgVO_3_. e) XRD patterns and crystal structures of Ag_2_V_4_O_11_. f) The ex situ XRD patterns of different voltage states collected at 50 mA g^−1^. g) CV curves of Ag_2_V_4_O_11_ at different scan rates. h) Rate performance of Ag_2_V_4_O_11_. i) Long‐term cycling performance of Ag_2_V_4_O_11_ at 5 A g^−1^. a–d) Reproduced with permission.^[^ ^299^ ^]^ Copyright 2019, Elsevier. e–i) Reproduced with permission.^[^ ^303^ ^]^ Copyright 2019, Wiley‐VCH.

The monoclinic channel‐structured *β*‐AgVO_3_ consists of an infinite number of [V_4_O_12_]_n_ double chains of edge‐shared VO_6_ octahedron, where the chains are zigzag in shape and double.^[^ ^297^, ^298^ ^]^ In addition, the [V_4_O_12_]_n_ double chains are composed of AgO_6_ octahedrons and tightly connected by Ag_2_O_5_ and Ag_3_O_5_ square pyramids to construct the robust open 3D network required for AZIBs. Liu et al.^[^ ^299^ ^]^ prepared an *β*‐AgVO_3_ with excellent performance for the cathode of AZIBs for the first time and demonstrated the basic storage mechanism of Zn^2+^ in detail. As can be seen from the XRD pattern in **Figure** **18**a, all the diffraction peaks belong to *β*‐AgVO_3_ monoclinic phase with channel structure, indicating high purity of the product. The in situ generation of Ag^0^ and residual Ag^+^ and structural water in the frame provide high electronic and ionic conductivity, which enhances the (de)intercalation kinetics of Zn^2+^ in the layered phase. The *β*‐AgVO_3_ cathode can provide an excellent rate performance of 103 mAh g^−1^ at 5 A g^−1^ (Figure 18b) and superior cycle stability of 95 mAh g^−1^ at 2 A g^−1^ after 1000 cycles (Figure 18c). Liu et al.^[^ ^299^ ^]^ also proved the energy storage mechanism of *β*‐AgVO_3_, as shown in Figure 18d.

##### Ag_2_V_4_O_11_

The crystal structure of Ag_2_V_4_O_11_ consists of [V_4_O_16_] units made of VO_6_ distorted octahedrons sharing their apexes, which build infinite [V_4_O_12_]_n_ quadruple strings. These quadruple strings are linked by corner‐shared oxygen to provide continuous [V_4_O_11_]_n_ layers separated by Ag atoms.^[^ ^300^, ^301^, ^302^ ^]^ Li and colleagues^[^ ^303^ ^]^ prepared a layered Ag_2_V_4_O_11_ via a facile hydrothermal method and used it as a novel cathode material for AZIBs. The XRD pattern can be labeled monoclinic Ag_2_V_4_O_11_ (Figure 18e). Ex situ XRD patterns of first two cycles within the working potential window of 0.4–1.7 V are shown in Figure 18f to study the electrochemical mechanism. For electrochemical performance of the Ag_2_V_4_O_11_ cathode, as shown in Figure 18g, when the scanning rate is from 0.1 to 0.5 mV s^−1^, the CVs shape doesn't change significantly, showing that its stability is good. In addition, the Ag_2_V_4_O_11_ cathode also delivers a specific capacity of 213 mAh g^−1^ (Figure 18h) and superior cycling performance with a capacity retention rate of 93% at 5 A g^−1^ after 6000 cycles (Figure 18i).

#### 4.3.2 The Intercalation of Zn^2+^

##### ZnV_2_O_4_

**Figure 19:**
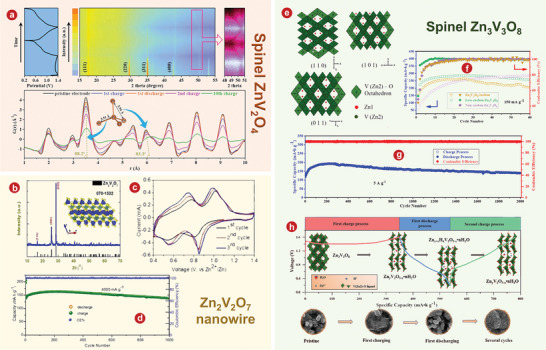
a) In situ XRD measurement of the ZnV_2_O_4_ electrode during the first two cycles (top) and ex situ PDF analysis of the ZnV_2_O_4_ electrode from 0 to 10 Å (bottom). b) Powder XRD pattern of *α*‐Zn_2_V_2_O_7_ prepared by a hydrothermal route. c) CV profiles of *α*‐Zn_2_V_2_O_7_ for AZIBs. d) Corresponding cyclability lifespan pattern for a prolonged 1000 cycles. e) The crystal structure diagrams of Zn_3_V_3_O_8_ in three different directions. f) Cycling performance of Zn_3_V_3_O_8_/carbon, less‐carbon Zn_3_V_3_O_8_ and non‐carbon Zn_3_V_3_O_8_ at a current density of 150 mA g^−1^ with saturated Zn(CF_3_SO_3_)_2_ as the electrolyte. g) Long‐term cycling performance of less‐carbon Zn_3_V_3_O_8_ at 5 A g^−1^ after electrochemical activation of several cycles. h) Schematic illustration of the significant (top) phase and (bottom) morphology evolution in AZIBs. a) Reproduced with permission.^[^ ^306^ ^]^ Copyright 2020, Elsevier. b–d) Reproduced with permission.^[^ ^310^ ^]^ Copyright 2018, The Royal Society of Chemistry. e–h) Reproduced with permission.^[^ ^314^ ^]^ Copyright 2021, Elsevier.

The crystal structure of ZnV_2_O_4_ belongs to the FCC‐type crystal structure with Fd3m̅ symmetric groups. The ZnO_4_ tetrahedron and VO_6_ octahedron form the crystal structure of ZnV_2_O_4_. Zn atoms are located at the (8a) tetrahedral position, while V atoms are located at the 16d tetrahedral position, forming a network of tetrahedral structures with shared angles. In addition, the O atoms are located at 32e.^[^ ^304^, ^305^ ^]^ Liu et al.^[^ ^306^ ^]^ studied a typical spinel ZnV_2_O_4_ as an AZIB cathode and observed an electroactivation reaction during the initial electrochemical cycle. The electroactivation reaction, which enhances surface electrochemical reactions through adaptive adjustment of lattice structures, is analyzed by in situ XRD, ex situ atomic pair distribution function (**Figure** **19**a), and various electrochemical measurements. The ZnV_2_O_4_ cathode delivers a high reversible capacity of 312 mAh g^−1^ and superior cycling performance with a capacity retention rate of 206 mAh g^−1^ after 1000 cycles at 10 C after electroactivation.

##### Zn_2_V_2_O_7_

Zn_2_V_2_O_7_ delivers polymorphism crystallizing in *α*‐ (*C2/c* space group) and *β*‐ (*C2/m* space group) forms.^[^ ^307^, ^308^ ^]^ Among them, *α*‐Zn_2_V_2_O_7_ is a promising yellow phosphor and an important component of transition‐metal vanadates with layered crystal structure.^[^ ^309^ ^]^ Sambandam and colleagues^[^ ^310^ ^]^ developed an AZIBs using 1D Zn_2_V_2_O_7_ nanowires, prepared by a simple one‐step hydrothermal method, as the potential (de)intercalation host. As shown in Figure 19b, the prepared powders are crystallographically characterized by powder XRD. The electrochemical process of multi‐step Zn^2+^ (de)intercalation caused by the reduction/oxidation of vanadium in the underlying *α*‐Zn_2_V_2_O_7_ is also explained by CV curves (Figure 19c).^[^ ^311^, ^312^, ^313^ ^]^ In addition, the *α*‐Zn_2_V_2_O_7_ cathode shows a good cycling performance with a capacity retention rate of 85% after 1000 cycles at an ultra‐high current drain of 4 A g^−1^ (Figure 19d).

##### Zn_3_V_3_O_8_

According to the structural parameters obtained by Rietveld refinement, Figure 19e is the crystal structure diagram of Zn_3_V_3_O_8_. Apparently, this structure has a 3D framework made of V(Zn_2_)O_6_ octahedron, and Zn1 ions are distributed in the tunnels along the directions [110], [101], and [011]. Therefore, spinel Zn_3_V_3_O_8_ is conducive to the transport of Zn^2+^ along the tunnels, but the improper deintercalation of Zn^2+^ during the charging process, especially the deintercalation of Zn^2+^ from the octahedral position, may lead to the collapse of spinel Zn_3_V_3_O_8_ structure. Spinel Zn_3_V_3_O_8_, as the first vanadium‐based compounds, was used as a high‐capacity cathode for AZIBs by Wu et al.^[^ ^314^ ^]^ All the three samples exhibit electroactivation in the incipient cycles, which is a common phenomenon related to the phase transition of cathode materials in AZIBs. Both less‐carbon and non‐carbon Zn_3_V_3_O_8_ show superior cycling performance, which delivers a maximal discharge capacity of 285 mAh g^−1^ (Figure 19f). In addition, the less‐carbon Zn_3_V_3_O_8_ delivers a capacity retention rate of 72.6% after 2000 cycles at 5 A g^−1^ (Figure 19g). Based on the above analysis and discussion of various representations, Wu et al.^[^ ^314^ ^]^ also show the structure and morphology evolution of the Zn_3_V_3_O_8_ cathode in Figure 19h.

#### 4.3.3 The Co‐Intercalation of Zn^2+^ and H_2_O

##### Zn_x_V_2_O_5_ *n*H_2_O

**Figure 20:**
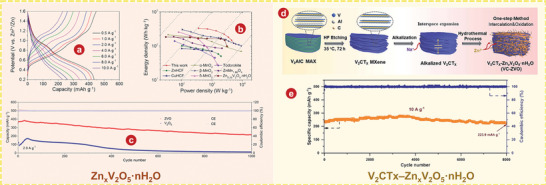
a) The charging/discharging curves of the Zn_x_V_2_O_5_ *n*H_2_O electrode. b) The Ragone plots of the Zn_x_V_2_O_5_ *n*H_2_O electrode. c) The cyclability performance at 2.0 A g^−1^ of the Zn_x_V_2_O_5_ *n*H_2_O electrode. d) The synthesis schematic of the V_2_CT_x_‐Zn_x_V_2_O_5_ *n*H_2_O heterostructure. e) Long‐term cycling performance of the V_2_CT_x_‐Zn_x_V_2_O_5_ *n*H_2_O electrode at 10 A g^−1^. a–c) Reproduced with permission.^[^ ^161^ ^]^ Copyright 2021, Elsevier. d, e) Reproduced with permission.^[^ ^317^ ^]^ Copyright 2021, The Royal Society of Chemistry.

The pre‐intercalated Zn^2+^ and H_2_O can also be used as “pillars” to stabilize the cathode and provide high Zn^2+^ storage, improving considerable battery performance. Hu and colleagues^[^ ^161^ ^]^ developed a method for the synthesis of Zn_x_V_2_O_5_ *n*H_2_O from vanadium trioxide metal by electrochemical intercalation phase transition in aqueous solution. The Zn_x_V_2_O_5_ *n*H_2_O nanosheets cathode delivers a high reversible capacity of 435 mAh g^−1^ at 0.5 A g^−1^ (**Figure** **20**a), high energy and power densities of 331 Wh kg^−1^ at 361 W kg^−1^ (Figure 20b), as well as superior cycle stability (Figure 20c). The addition of highly conductive substrates, such as carbon‐based materials,^[^ ^315^ ^]^ conductive polymers,^[^ ^316^ ^]^ and MXenes,^[^ ^230^ ^]^ can indeed enhance the Zn^2+^ storage capacity of vanadium‐based cathodes at higher rates. However, such preparation of such complexes is complicated and uncertain, and cannot maintain structural stability during cycling. In order to solve these problems, Zhu and colleagues^[^ ^317^ ^]^ used highly conductive V_2_CT_x_ MXene to fabricate Zn_x_V_2_O_5_ *n*H_2_O nanoribbon (V_2_CT_x_‐Zn_x_V_2_O_5_ *n*H_2_O) with uniform size by the simultaneous action of ion intercalation and oxidation (Figure 20d), and used it as cathode material for AZIBs. Due to the pre‐intercalation of Zn^2+^ and the ubiquitous interfaces between Zn_x_V_2_O_5_ *n*H_2_O and the conductive network including the remaining V_2_CT_x_ and carbon, the charge redistribution in the active/conductive heterostructure leads to the weakening of electrostatic interactions, fast Zn^2+^ (de)intercalation, and structural stability, which makes the V_2_CT_x_‐Zn_x_V_2_O_5_ *n*H_2_O cathode show an excellent cycling performance with a capacity retention of 96.4% more than 8000 cycles at 10 A g^−1^ (Figure 20e).

#### 4.3.4 The Intercalation of Other Transition Metal Cations

##### CrVO_3_

**Figure 21:**
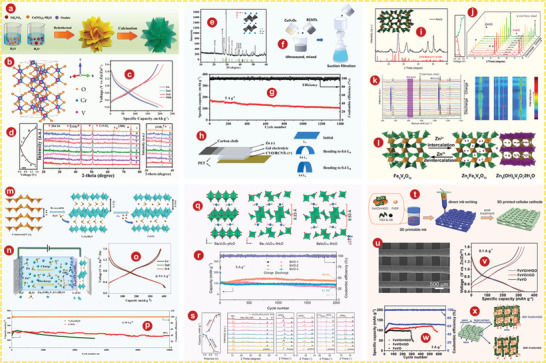
a) Schematic illustration of the fabrication urchin‐like porous CrVO_3_. b) Crystal structure of pr‐CVO‐1. c) Charge‐discharge profiles of pr‐CVO‐1 at 0.5 A g^−1^. d) The first charge–discharge of pr‐CVO‐1, XRD patterns of the pr‐CVO‐1 cathodes at various GCD cycles, and magnified XRD patterns. e) XRD pattern of CuV_2_O_6_‐18. f) Schematic illustration of vacuum filtration. g) The long‐term cycling performance of CuV_2_O_6_‐18/RCNTs at the current densities of 2 A g^−1^. h) Schematic diagram of flexible gel Zn//CuV_2_O_6_‐18/RCNTs battery (reaction time 18 h). i) XRD pattern of Fe_2_V_4_O_13_. j) Charge/discharge profiles for the initial cycle at 0.2 A g^−1^ and corresponding ex situ XRD patterns of Fe_2_V_4_O_13_ cathode during discharge‐charge process. k) In situ Raman curves of Fe_2_V_4_O_13_ cathode during discharge‐charge process. l) Schematic diagram of the (de)intercalation process of Zn^2+^ in Fe_2_V_4_O_13_ during cycling. m) Schematic diagram of fabrication process for Cs^+^ intercalated V_2_O_5_ *n*H_2_O. n) Schematic diagram of Zn//Cs_0.53_V_2_O_5_ 0.58H_2_O battery. o) The discharge/charge profiles at 0.1 A g^−1^ of the first three cycles of Cs_0.53_V_2_O_5_ 0.58H_2_O cathode. p) The long cycle performance of two cells at 20 A g^−1^. q) The crystal structure of Ba_x_V_2_O_5_ *n*H_2_O, Ba_1.2_V_6_O_16_ 3H_2_O, and BaV_6_O_16_ 3H_2_O. r) Cycling performance of three barium vanadates at 5Ag^−1^. s) Charge/discharge curve at the selected states of the second cycle at 200 mA g^−1^ for the Ba_1.2_V_6_O_16_ 3H_2_O cathode. Sampling points for XRD and FTIR characterization were marked with the corresponding‐colored dots and ex situ XRD patterns and the corresponding magnified XRD patterns of different peaks for the Ba_1.2_V_6_O_16_ 3H_2_O cathode. t) Schematic of direct ink writing‐based fabrication of cellular Fe_5_V_15_O_39_(OH)_9_ 9H_2_O/rHGO cathodes for AZIBs. u) Top‐viewed of cellular Fe_5_V_15_O_39_(OH)_9_ 9H_2_O/rHGO inks. v) GCD profiles of 3D Printed Fe_5_V_15_O_39_(OH)_9_ 9H_2_O/rHGO cathodes at different current densities. w) Long‐term cycling performance at a current density of 2 A g^−1^. x) Schematic of ionic transport for 3D printed Fe_5_V_15_O_39_(OH)_9_ 9H_2_O/rHGO and Fe_5_V_15_O_39_(OH)_9_ 9H_2_O/rGO electrodes. a–d) Reproduced with permission.^[^ ^318^ ^]^ Copyright 2021, Elsevier. e–h) Reproduced with permission.^[^ ^319^ ^]^ Copyright 2022, Elsevier. i–l) Reproduced with permission.^[^ ^320^ ^]^ Copyright 2022, Elsevier. m–p) Reproduced with permission.^[^ ^321^ ^]^ Copyright 2022, Elsevier. q–s) Reproduced with permission.^[^ ^76^ ^]^ Copyright 2020, American Chemical Society. t–x) Reproduced with permission.^[^ ^326^ ^]^ Copyright 2021, Wiley‐VCH.

CrVO_3_ crystals have an open‐channel structure and play a key role in the process of Zn^2+^ (de)intercalation. Bai et al.^[^ ^318^ ^]^ prepared a novel CrVO_3_ with an urchin‐like porous structure via a simple hydrothermal followed by calcination (**Figure** **21**a). The pr‐CVO‐1, pr‐CVO‐2, and pr‐CVO‐2 were synthesized according to different amounts of Cr(NO_3_)_3_ 9H_2_O added. The crystal structure of pr‐CVO‐1 is shown in Figure 21b. The pr‐CVO‐1 shows the best electrochemical performance with a first discharge capacity of 188.8 at 0.5 A g^−1^ (Figure 21c). At the same time, the formation mechanism and storage mechanism of Zn^2+^ were discussed by ex situ method (Figure 21d). The results show that porous CrVO_3_ is a promising cathode material for AZIBs, which provides a valuable design idea for significantly improving the electrochemical energy storage performance of porous vanadates.

##### CuV_2_O_6_

The crystal structure of CuV_2_O_6_ consists of a double‐layered, serrated VO_6_ octahedral structure with split edges along the b axis. Song and colleagues^[^ ^319^ ^]^ prepared CuV_2_O_6_ nanobelts by hydrothermal method and free CuV_2_O_6_/reductively acidified CNTs (CuV_2_O_6_/RCNTs) composite films without binder by vacuum filtration method. The aqueous Zn//CuV_2_O_6_/RCNTs batteries have a good reversible capacity of 353 mA g^−1^ at 0.1 A g^−1^, and a high reversible capacity of 174.7 mA g^−1^ and a capacity retention of 61.5% after 1400 cycles of 5 A g^−1^ (Figure 21g). Song et al.^[^ ^319^ ^]^ also assemble flexible gel Zn//CuV_2_O_6_‐18/RCNTs battery, as shown in Figure 21h.

##### Fe_2_V_4_O_13_

The tunnel structure of monoclinic Fe_2_V_4_O_13_ consists of VO_4_ tetrahedron and FeO_6_ octahedron, which makes possible and favorable conditions for reversible (de)intercalation of Zn^2+^. Yang et al.^[^ ^320^ ^]^ synthesized a Fe_2_V_4_O_13_ with open structure as a cathode material for AZIBs. The structure of the prepared Fe_2_V_4_O_13_ sample is confirmed by XRD characterization, and the diffraction peak has good directivity with the monoclinic Fe_2_V_4_O_13_ phase (Figure 21i). Interestingly in this work, Yang et al.^[^ ^320^ ^]^ demonstrated that two Zn^2+^ storage mechanisms could be observed simultaneously with Fe_2_V_4_O_13_ cathode through a combination of in situ and ex situ techniques (Figure 21j,k), namely, the classical (de)intercalated storage mechanism in the Fe_2_V_4_O_13_ tunnel structure and the reversible phase transition from ferric vanadate to zinc vanadate (Figure 21l).

#### 4.3.5 The Intercalation of Other Transition Metal Cations and H_2_O

##### Cs_0.53_V_2_O_5_ 0.58H_2_O

The pre‐intercalated Cs^+^ with large ionic radii preserves the appropriate interlayer distance for the diffusion of Zn^2+^, while the Cs^+^ as the “pillar”, the strong Cs—O bond in the interlayer structure effectively maintains the stability of the structure, thus improving the rate capacity and cycling performance. Qi et al.^[^ ^321^ ^]^ inserted Cs^+^ into V_2_O_5_ *n*H_2_O, resulting in enhanced layered structures that form strong CS—O bonds with native oxygen atoms to enhance interlayer interactions and avoid structural collapse (Figure 21m). The electrochemical performance of Cs_0.53_V_2_O_5_ 0.58H_2_O for storing Zn^2+^ was studied by assembling it with zinc foil anode and 3m Zn(CF_3_SO_3_)_2_ electrolyte (Figure 21n). The Cs_0.53_V_2_O_5_ 0.58H_2_O cathode delivers an improved specific capacity of 404.9 mAh^−1^ at 0.1 A g^−1^ (Figure 21o) and superior long‐term cycle stability with a capacity retention of 89% after 10 000 cycles at 20 A g^−1^ (Figure 21p).

##### Ba_1.2_V_6_O_16_ 3H_2_O

Both BaV_6_O_16_ 3H_2_O and Ba_1.2_V_6_O_16_ 3H_2_O (typical V_3_O_8_‐typed structure) are composed of V_3_O_8_ polyhedral layers, which are stabilized by Barium hydrate ions, while the Ba_x_V_2_O_5_ *n*H_2_O is a typical V_2_O_5_ structure due to the deviation of V—O band. The two layers of V_2_O_5_ are bound by V—O bonds between layers, and the distributed barium hydrate ions are supported between layers (Figure 21q). Wang and colleagues^[^ ^76^ ^]^ controlled the synthesis of three barium vanadate nanobelt cathodes by adjusting the amount of barium precursor. Thanks to the robust structure, the layered Ba_1.2_V_6_O_16_ 3H_2_O nanobelt can effectively inhibit cathodic dissolution due to the rapid zinc ion kinetics, showing better rate capability and long‐term cyclability than the other two (Figure 21r). In addition, these robust characteristics and water co‐intercalation phenomena were revealed by electrochemical mechanism studies characterized by ex situ XRD, FTIR (Figure 21s), and so on. Wang and colleagues^[^ ^76^ ^]^ provides a feasible strategy for exploring or designing cathodic materials with robust structures to enhance the electrochemical performance of AZIBs.

##### Fe_5_V_15_O_39_(OH)_9_ 9H_2_O

Recently, advanced 3D printing of cellular and hierarchical porous cathodes with high mass loading for AZIBs with excellent performance is explored,^[^ ^322^, ^323^ ^]^ which has unique manufacturing advantages of custom design, rapid prototyping, and structural optimization.^[^ ^324^, ^325^ ^]^ Ma et al.^[^ ^326^ ^]^ composed a nanocomposite ink composed of Fe_5_V_15_O_39_(OH)_9_ 9H_2_O nanosheet and reduced porous graphene oxide (rHGO) as the active material for the cell cathode, and extruded 3D printing inks with good rheological control properties onto various substrates to form independent nanocomposite cathodes (Figure 21t). The 3D printed‐Fe_5_V_15_O_39_(OH)_9_ 9H_2_O/*r*HGO and 3D printed‐Fe_5_V_15_O_39_(OH)_9_ 9H_2_O/rGO cathodes are composed of crisscrossing columns with a column diameter of about 390 µm from SEM image (Figure 21u). The 3D printed‐Fe_5_V_15_O_39_(OH)_9_ 9H_2_O/rHGO cathode with high mass loading over 10 mg cm^−2^ shows a high specific capacity of 344.8 mAh g^−1^ at 0.1 A g^−1^ (Figure 21v) and delivers superior cycling stability over 650 cycles at 2 A g^−1^ (Figure 21w). In addition, Figure 21x clearly illustrates that the 3D‐printed cellular structure can provide open channels as well as large contacts with the electrolyte, leading to 3D migration of ions throughout the electrode structure.

### 4.4 Ammonium Cations

#### 4.4.1 The Intercalation of NH_4_^+^

##### (NH_4_)_0.38_V_2_O_5_

**Figure 22:**
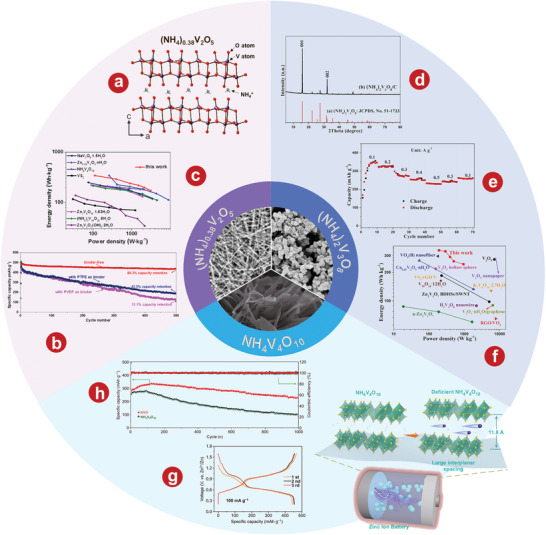
a) The atomic structure of layered (NH_4_)_0.38_V_2_O_5_ crystal. b) The cycle performance comparison between the (NH_4_)_0.38_V_2_O_5_/CNTs paper electrode and the control electrode prepared through slurrying with PVDF/PTFE as binder. c) Ragone plots of (NH_4_)_0.38_V_2_O_5_/CNTs paper electrode compared to other cathodes for AZIBs. d) XRD pattern of (NH_4_)_2_V_3_O_8_/C. e) Rate performance of Zn//(NH4)_2_V_3_O_8_/C battery. f) Comparison of Ragone plots of Zn//(NH_4_)_2_V_3_O_8_/C battery with those reported for vanadium‐based electrodes applied to AZIBs (the value is based on the mass of the cathode material). g) GCD curves of NH_4_V_4_O_10_ in first three cycles. h) Cycling stability with the corresponding coulombic efficiencies at 2 A g^−1^.a–c) Reproduced with permission.^[^ ^330^ ^]^ Copyright 2021, Elsevier. d–f) Reproduced with permission.^[^ ^339^ ^]^ Copyright 2020, Elsevier. g, h) Reproduced with permission.^[^ ^340^ ^]^ Copyright 2021, Springer.

The monoclinic (NH_4_)_0.38_V_2_O_5_ unit structure consists of distorted VO_6_ octahedrons with shared edges, forming a stable bilayer structure (**Figure** **22**a). The oxygen atoms in the octahedron have strong interactions with NH_4_^+^. NH_4_^+^ tends to act as “pillar” cations to stabilize the structure and prevent volume changes in the interlayer spacing of guest ions during (de)intercalation.^[^ ^327^ ^]^ In addition, compared with other vanadates such as sodium and potassium, ammonium cations exhibit relatively small molecular weight and density, and provide higher specific gravity and volumetric capacity.^[^ ^328^, ^329^ ^]^ Jiang et al.^[^ ^330^ ^]^ revealed the spontaneous knitting behavior of 6.7 nm thin, flexible (NH_4_)_0.38_V_2_O_5_ nanoribbons and the formation of binder‐free paper ZIBs cathodes via hydrothermal pathways. Conductive CNTs have also been successfully embedded in paper to improve electronic conductivity and generate rich grids inside the paper. Due to the advantages of the binder‐free design and porous structure, the (NH_4_)_0.38_V_2_O_5_/CNTs paper cathode has excellent long‐term cycling performance, with an initial specific capacity of 465 mAh g^−1^, which still maintains an initial specific capacity of 89.3% after 500 cycles at A rate of 0.1 A g^−1^ (Figure 22b). In addition, as shown in Figure 22c, the paper cathode has a specific energy of up to 343 Wh kg^−1^, which is significantly better than most powder cathodic ZIBs containing polymer binders.^[^ ^331^, ^332^, ^333^, ^334^, ^335^ ^]^

##### (NH_4_)_2_V_3_O_8_

(NH_4_)_2_V_3_O_8_ is a typical layered structure consisting of V_3_O_8_ layers and interstitial NH_4_^+^. The VO layer consists of VO_4_ tetrahedron (located in the plane of symmetry; The difference between the longest and shortest V—O bond is 0.08 Å) and VO_5_ square pyramid, with NH_4_^+^ in the interlayer. The VO_4_ tetrahedron and the VO_5_ pyramid are linked by O atoms to form thin sheets parallel to (0 0 1).^[^ ^336^ ^]^ These structural features can be used in SIBs and LIBs to store Na^+^ and Li^+^. NH_4_^+^ in (NH_4_)_2_V_3_O_8_ is located in the tetrahedral site between the V and O atomic layers and can be occupied by Na^+^ or Li^+^.^[^ ^337^, ^338^ ^]^ The ionic radius of Zn^2+^ (0.76 Å) is smaller than that of NH_4_^+^ (1.43Å), so it is allowed to reversibly (de)intercalate Zn^2+^ in (NH_4_)_2_V_3_O_8_ cathode and adapt to volume expansion. Jiang and colleagues^[^ ^339^ ^]^ reported for the first time that (NH_4_)_2_V_3_O_8_ nanoparticles were encapsulated into an amorphous carbon matrix as AZIBs cathode with high capacity. However, carbon is not observed in the XRD pattern (Figure 22d), this is because most of the carbon phase prepared by hydrothermal method is amorphous carbon, which cannot be detected by XRD. (NH_4_)_2_V_3_O_8_ did not crystallize well in HRTEM because it was covered by a layer of amorphous carbon. Zn//(NH_4_)_2_V_3_O_8_/C battery has significantly enhanced electrochemical performance, with a specific capacity of 356 mAh g^−1^ at 0.1 A g^−1^ (Figure 22e), high‐rate performance and cycle life of 135 mA h^−1^ after 2000 cycles at 1 A g^−1^, as well as the energy density of 334 Wh kg^−1^ at 294 W kg^−1^ (Figure 22f).

##### NH_4_V_4_O_10_

Monoclinic NH_4_V_4_O_10_ consists of a distorted VO_6_ octahedron. The vanadium octahedron shares an edge, forming a stable bilayer structure that includes V_4_O_10_ units stacked along the *α*‐axis.^[^ ^332^ ^]^ Zong et al.^[^ ^340^ ^]^ synthesized 2D NH_4_V_4_O_10_ nanosheets by heat‐treating NH_4_V_4_O_10_ nanosheets grown on CC at low temperature in air. The increased interlayer spacing of NH_4_V_4_O_10_ is conducive to the rapid migration of Zn^2+^ and high storage capacity, which ensures the high reversibility of the electrochemical reaction and the good stability of the layered structure. The NH_4_V_4_O_10_ nanosheets have a high specific capacity of 457 mAh g^−1^ at 0.1 A g^−1^ (Figure 22g) and superior cycle stability with a capacity retention of 81% after 1000 cycles at 2 A g^−1^ (Figure 22h). Huang et al.^[^ ^341^ ^]^ used the NH_4_V_4_O_10_ as an example to optimize engineering by selecting the electrolyte and adjusting the proportion of conductive carbon in the electrode. The NH_4_V_4_O_10_‐541 electrode (NH_4_V_4_O_10_ sample, acetylene black to poly(vinylidene difluoride) in a weight ratio of 50:40:10) can provide a high reversible capacity of 430.0 mAh g^−1^ at 0.1 A g^−1^, good speed capacity of 277.1 mAh g^−1^ at 10 A g^−1^, as well as superior cycle stability with a capacity retention of 72.2% over 3000 cycles at 10 A g^−1^. Sun and colleagues^[^ ^342^ ^]^ proposed a self‐template method for the synthesis of NH_4_V_4_O_10_ with decussate structure and intercalation mechanism by a simple one‐step hydrothermal method, which achieved a remarkable mass‐energy density of 332.25 Wh kg^−1^, excellent rate performance, and stable cycle stability. In order to improve the cycling stability and diffusion rate of vanadium‐based compounds, doping other electrochemically active substances (e.g., Ti) into the compounds is an effective method.^[^ ^343^, ^344^, ^345^, ^346^ ^]^ He et al.^[^ ^347^ ^]^ prepared Ti‐doped NH_4_V_4_O_10_ using a robust bilayer structure, which not only ensured rapid and reversible Zn^2+^ intercalation, but also reduced the accumulation of Zn^2+^. Compared with the pure NH_4_V_4_O_10_, the Ti‐doped NH_4_V_4_O_10_ has faster diffusion kinetics, higher electrochemical reversibility, and better structural stability. For example, at 2 A g^−1^, the capacity retention rate of Ti‐doped NH_4_V_4_O_10_ after 2000 cycles is 89.02%, which is much higher than that of NH_4_V_4_O_10_ (62.86%).

#### 4.4.2 The Co‐Intercalation of NH_4_^+^ and H_2_O

##### NH_4_V_3_O_8_ 0.5H_2_O

NH_4_V_3_O_8_ 0.5H_2_O is made up of 2D V_3_O_8_ layers, consisting of VO_6_ octahedrons and VO_5_ square pyramids by sharing corners, and are pinned together by the NH_4_^+^.^[^ ^332^ ^]^ In addition, the electrochemical performance of NH_4_V_3_O_8_ 0.5H_2_O is significantly improved by the intercalation of H_2_O molecules in the layered structure. Jiang et al.^[^ ^348^ ^]^ prepared NH_4_V_3_O_8_ 0.5H_2_O nanobelts by low‐temperature hydrothermal to prove that the intercalation of H_2_O molecules in the layer structure had a strong enhancement effect on the electrochemical performance of NH_4_V_3_O_8_. NH_4_V_3_O_8_ 0.5H_2_O nanobelts have an ultra‐high capacity of 423 mAh g^−1^ at 0.1 A g^−1^ and maintain long‐term stability of 50.1% after 1000 cycles at 1 A g^−1^.

##### The Intercalation of Polyaniline in NH_4_V_3_O_8_ 0.5H_2_O

**Figure 23:**
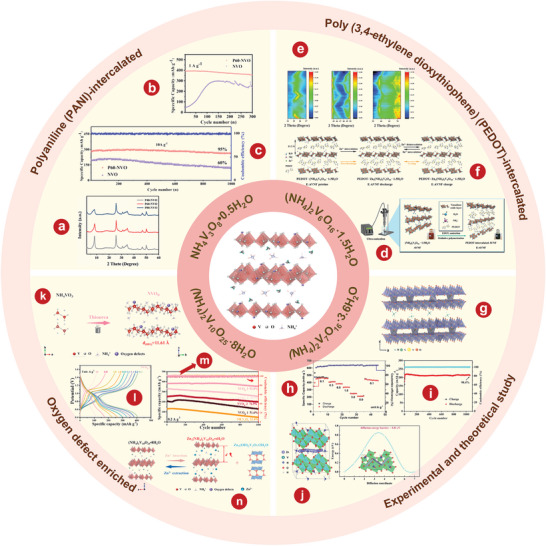
a) XRD patterns of P(x)‐NH_4_V_3_O_8_ 0.5H_2_O (x = 40,60,80) with different aniline concentration. Cycling performance of NH_4_V_3_O_8_ 0.5H_2_O and P60‐NH_4_V_3_O_8_ 0.5H_2_O electrodes at b) 1 and c) 10 A g^−1^. d) Schematic illustration of PEDOT and ammonium ion intercalation into the vanadate layer via sonication. e) In situ XRD contour plots of E‐AVNF within selected scanning angle (2*θ*) domains of c) 24°–27°, 44.5°–50°, and 55°–72°. f) Schematic illustrations of Zn^2+^ intercalation/de‐intercalation process into E‐AVNF during cycling. g) Crystal structure of (NH_4_)_2_V_7_O_16_ viewed along the *b*‐axis. h) Rate performance of the (NH_4_)_2_V_7_O_16_ 3.6H_2_O cathode. i) Long‐term stability of the (NH_4_)_2_V_7_O_16_ 3.6H_2_O cathode at 5 A g^−1^. j) The simulated diffusion pathway of Zn^2+^ in (NH_4_)_2_V_7_O_16_ (side view) and the corresponding diffusion energy barrier profiles (the inset is the diffusion pathway in top view). k) Schematic diagram of the preparation of oxygen defect enriched (NH_4_)_2_V_10_O_25_ 8H_2_O. l) GCD profiles of oxygen defect enriched (NH_4_)_2_V_10_O_25_ 8H_2_O. m) Cyclability of oxygen defect enriched (NH_4_)_2_V_10_O_25_ 8H_2_O and NH_4_V_3_O_8_ obtained at 0.2 A g^−1^. n) Schematic diagram of the Zn^2+^ insertion mechanism on (NH_4_)_2_V_10_O_25_ 8H_2_O cathode. a–c) Reproduced with permission.^[^ ^355^ ^]^ Copyright 2022, Elsevier. d–f) Reproduced with permission.^[^ ^360^ ^]^ Copyright 2021, Wiley‐VCH. g–j) Reproduced with permission.^[^ ^365^ ^]^ Copyright 2021, Elsevier. k–n) Reproduced with permission.^[^ ^367^ ^]^ Copyright 2021, Elsevier.

The structure of PANI contains reducing units, so PANI may undergo redox reactions with host substances in the reaction system.^[^ ^349^ ^]^ Therefore, PANI can be in situ intercalated into host materials to form hybrid materials during redox reactions.^[^ ^350^, ^351^ ^]^ In addition, the redox reaction of PANI with the host material in the reaction system may generate oxygen vacancies, which is conducive to the improvement of electron mobility.^[^ ^352^, ^353^, ^354^ ^]^ Li and colleagues^[^ ^355^ ^]^ designed an organic‐inorganic (ammonium vanadate) hybrid cathode with extended layer spacing by intercalating polyaniline into the interlayer of NH_4_V_3_O_8_ 0.5H_2_O. After polyaniline intercalation, as shown in **Figure** **23**a, the interlayer distance of NH_4_V_3_O_8_ 0.5H_2_O significantly increased from 7.9 to 10.8 Å, providing fast channel for the diffusion of Zn^2+^. The organic‐inorganic (ammonium vanadate) hybrid cathode has good electrochemical performance, with a high initial capacity of 397.5 mAh g^−1^ at 1 A g^−1^ (Figure 23b) and good cycle stability of 300 mAh g^−1^ at 10 A g^−1^ with a capacity retention rate of 95% after more than 1000 cycles (Figure 23c) due to the intercalation of PANI.

##### (NH_4_)_2_V_6_O_16_ 1.5H_2_O

In the structure of (NH_4_)_2_V_6_O_16_ 1.5H_2_O, VO_5_ square pyramid and VO_6_ octahedral chains form V_3_O_8_ layers along the z‐axis by sharing the corners, and hydrated NH_4_^+^ in interstitial sites act as “pillars” to stabilize the layered structure. Because the valence state (+5) of V in the (V_6_O_16_)^2−^ structure is higher than that of M(M = metal)V_2_O_5_ *n*H_2_O, (NH_4_)_2_V_6_O_16_ 1.5H_2_O is able to accommodate more Zn^2+^.^[^ ^21^ ^]^ Wang et al.^[^ ^356^ ^]^ presented a highly reversible AZIB system with (NH_4_)_2_V_6_O_16_ 1.5H_2_O nanobelts as cathode materials by one‐step hydrothermal method and ZnSO_4_ aqueous solution as electrolyte. The (NH_4_)_2_V_6_O_16_ 1.5H_2_O nanobelts cathode has an excellent reversible specific capacity of 479 mAh g^−1^ with an ideal energy density of 371.5 Wh kg^−1^ at 0.1 A g^−1^, and has satisfactory cycle stability, which is 152 mAh g^−1^ maintained more than 3000 cycles at 5 A g^−1^. Chen and coworkers^[^ ^357^ ^]^ fabricated the (NH_4_)_2_V_6_O_16_ 1.5H_2_O nanostructure by a simple microwave‐assisted hydrothermal reaction and studied the structure information and zinc storage properties of (NH_4_)_2_V_6_O_16_ 1.5H_2_O in detail. The Zn(H_2_O)_6_^2+^ captures during the initial discharge not only help stabilize the vanadium oxide layer, but also provide the sufficient interlayer distance for fast ionic dynamics during the previous (de)intercalation.

##### The Intercalation of Poly(3,4‐Ethylene Dioxythiophene) in (NH_4_)_2_V_6_O_16_ 1.5H_2_O

The electrical conductivity can be improved by intercalating PEDOT into the vanadate nanofiber lattice. In addition, the intercalated conductive polymer serves as a more solid pillar for the vanadate layered structure, achieving stable (dis)charging compared to the cationic and water molecules. By extending the distance between vanadate crystal planes through the intercalation of PEDOT, the melting rate of electrolyte cations can also be increased, thus making vanadate with excellent rate capability. In addition, the lubricating effect of the intercalated PEDOT prevents the trapping of electrolyte ions.^[^ ^358^, ^359^ ^]^ Kim et al.,^[^ ^360^ ^]^ for the first time, enhanced the rate capability, electrochemical reversibility, and cyclic stability of ammonium vanadate nanofiber (AVNF) as a AZIBs cathode material by using PEDOT to control the interlayer structure of AVNF crystals via a sample sonochemical method (Figure 23d). In situ XRD contour plots of PEDOT‐AVNF (E‐AVNF) within selected scanning angle (2*θ*) domains of 24°–27°, 44.5°–50°, and 55°–72° are shown in Figure 23e to further investigate the (dis)charging mechanism of the E‐AVNF electrodes. The Zn^2+^ storage mechanism of E‐AVNF is shown in Figure 23f. The results demonstrate that the control of the intermediate layer through the spacer ammonium vanadate of PEDOT results in rapid diffusion of Zn^2+^, reversible electrochemical reactions, and high performance of AZIBs.

##### (NH_4_)_2_V_7_O_16_ 3.6H_2_O

As a new type of ammonium vanadate, (NH_4_)_2_V_7_O_16_ 3.6H_2_O has been rarely studied. Despite the unprecedented stoichiometry and crystal structure, the layered structure is similar to the others. The structure consists of stacked layers of V_7_O_16_ aligned along the *c*‐axis and two NH_4_^+^ per formulation unit occupying the interlayer space. Each NH_4_^+^ is hydrogen bonded to four lattice oxygen atoms to form a stable structure with a large interlayer space, which enables the intercalation of various visiting ions. Unlike ethylene diamine vanadate, which was previously reported to be intercalated by neutral molecules, the interlayer space of (NH_4_)_2_V_7_O_16_ is occupied by NH_4_^+^.^[^ ^361^ ^]^ In addition, the average oxidation state of vanadium ions in the V_7_O_16_ layer is 4.29+ and the formal charge is 2‐, which is lower than that of other ammonium vanadates.^[^ ^362^, ^363^, ^364^ ^]^ Wang et al.^[^ ^365^ ^]^ successfully fabricated attractive (NH_4_)_2_V_7_O_16_ 3.6H_2_O nanoplates via a facile hydrothermal reaction and applied them in AZIBs. The crystal structure (NH_4_)_2_V_7_O_16_ is shown in Figure 23g. The unique structure of NH_4_^+^ intercalated in the V_7_O_16_ layer expands the layer spacing to 9.1 Å, and the capacity reaches 465.0 mAh g^−1^ at 0.1 A g^−1^ (Figure 23h), as well as the capacity retention rate is 98.4% at 5A g^−1^ (Figure 23i). In addition, the reversible (dis)charging process and kinetic behavior of (NH_4_)_2_V_7_O_16_ 3.6H_2_O electrode are investigated by DFT calculations (Figure 23j) and ex situ XRD.

##### (NH_4_)_2_V_10_O_25_ 8H_2_O

(NH_4_)_2_V_10_O_25_ 8H_2_O describes a characteristic layered structure consisting of the VO layer and interstitial NH_4_^+^ and H_2_O. NH_4_^+^ and H_2_O in (NH_4_)_2_V_10_O_25_ 8H_2_O are located at the tetrahedral sites between the layers of V and O atoms and among the VO layers, which are available for occupation by metal ions (e.g., Zn^2+^).^[^ ^365^, ^366^ ^]^ Cao et al.^[^ ^367^ ^]^ reasonably designed an advanced oxygen defect enriched (NH_4_)_2_V_10_O_25_ 8H_2_O nanosheet cathode (Figure 23k) with extended tunnel structure, excellent electrical conductivity, and structural stability, exhibiting rapid Zn^2+^ diffusion and superior performance. The AZIB with oxygen defect enriched (NH_4_)_2_V_10_O_25_ 8H_2_O nanosheets cathode has a very high capacity of 408 mA h g^−1^ at 0.1 A g^−1^ (Figure 23l), long‐time stability of 94.1% retention over 4000 cycles (Figure 23m) and superior energy density of 287 Wh kg^−1^. As shown in Figure 23n, the electrochemical mechanism of oxygen defect enriched (NH_4_)_2_V_10_O_25_ 8H_2_O cathode based on reversible Zn^2+^ intercalation is demonstrated by a variety of characterization techniques (e.g., ex situ XRD pattern). Bai and colleagues^[^ ^368^ ^]^ developed an advanced stainless steel (SS)‐supported oxygen‐rich vacancy (NH_4_)_2_V_10_O_25_ 8H_2_O cistern‐like nanobelts cathode with widened layer spacing and ultrafast reaction kinetic. The SS‐supported oxygen‐rich vacancy (NH_4_)_2_V_10_O_25_ 8H_2_O cistern‐like nanobelt cathode has a high capacity of 331.4 mAh g^−1^ at 0.3 A g^−1^, superior rate performance, and excellent long‐time stability of 78.3 mAh g^−1^ more than 7500 cycles at 4.8 A g^−1^.

### 4.5 Mixture of Different Kinds of Cations

#### 4.5.1 The Co‐Intercalation of Different Kinds of Cations

##### K_x_(NH_4_)_y_V_4_O_10_

**Figure 24:**
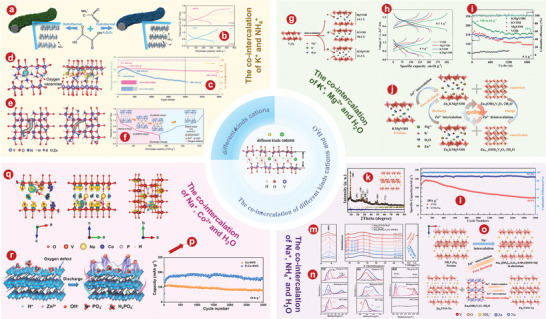
a) Schematic illustration of the preparation process and the crystal structure of two kinds of samples. b) voltage profiles at 0.1 A g^−1^ in the first three cycles. c) Cycling performance at 5 A g^−1^. d) Charge density difference analysis of (left) NH_4_V_4_O_10_ cathode and (right) K_x_(NH_4_)_y_V_4_O_10_ cathode. e) Possible migration pathways for Zn^2+^ in (left) NH_4_^+^‐intercalation sample cathode and (right) K^+^ and NH_4_^+^‐intercalation sample cathode. f) Schematic illustrations of Zn^2+^ storage mechanism of K^+^ and NH_4_^+^‐intercalation sample cathode. g) The diagram of the Mg_x_V_2_O_5_ *n*H_2_O, K_x_V_2_O_5_ *n*H_2_O, and K_0.09_Mg_0.03_V_2_O_5_ *n*H_2_O crystal structures. h) GCD graphics collected at 0.1 and 4 A g^−1^ of four materials. i) Cycling performance of samples tested at 4 A g^−1^. j) Schematic diagram of the zinc (de)intercalation mechanism in the K_0.09_Mg_0.03_V_2_O_5_ *n*H_2_O electrode. k) XRD pattern of Na_0.3_(NH_4_)_0.6_V_4_O_10_ 0.4H_2_O. l) Cycle ability of NH_4_V_4_O_10_ and Na_0.3_(NH_4_)_0.6_V_4_O_10_ 0.4H_2_O obtained at 10 A g^−1^. m) Ex situ XRD patterns for Na_0.3_(NH_4_)_0.6_V_4_O_10_ 0.4H_2_O under diverse charge/discharge states. n) Ex situ XPS spectra for (left) V *2p*, (middle) O *1 s* together with (right) Zn *2p* regions at various states. o) Schematic diagram about crystal structure change of Na_0.3_(NH_4_)_0.6_V_4_O_10_ 0.4H_2_O in cycling process. p) Cycling performance of Na_1.04_Co_0.54_V_8_O_20_ 1.1H_2_O and P(phosphating)‐Na_1.04_Co_0.54_V_8_O_20_ 1.1H_2_O at 10 A g^−1^. q) Electron density difference of P‐Na_1.04_Co_0.54_V_8_O_20_ 1.1H_2_O. r) The schematics of crystal structure evolution of P‐Na_1.04_Co_0.54_V_8_O_20_ 1.1H_2_O. a–f) Reproduced with permission.^[^ ^370^ ^]^ Copyright 2022, American Chemical Society. g–j) Reproduced with permission.^[^ ^372^ ^]^ Copyright 2021, Elsevier. k–o) Reproduced with permission.^[^ ^373^ ^]^ Copyright 2022, Elsevier. p–r) Reproduced with permission.^[^ ^375^ ^]^ Copyright 2021, Elsevier.

Compared with only NH_4_^+^ intercalation, the addition of monovalent basic alkaline cations can be intercalated into the interlayer space to strengthen ionic bonds and thus stabilize the layered structure.^[^ ^369^ ^]^ Zong et al.^[^ ^370^ ^]^ synthesized potassium ammonium vanadate as the cathode for AZIB by substituting part of the NH_4_^+^ between NH_4_V_4_O_10_ layers with K^+^. The schematic illustration of the preparation process and the crystal structure of NH_4_V_4_O_10_ and K_x_(NH_4_)_y_V_4_O_10_ is shown in **Figure** **24**a. The intercalation of K^+^ results in a subtle shrinkage of the ammonium vanadate lattice distance and an increase in oxygen vacancies. As expected, K_x_(NH_4_)_y_V_4_O_10_ has a better discharge capacity of 464 mAh g^−1^ than NH_4_V_4_O_10_ (391 mAh g^−1^) at 0.1 A g^−1^ (Figure 24b), and good cycle stability with a retention of 90% more than 3000 cycles at 5 A g^−1^(Figure 24c). As shown in Figure 24d,e, DFT calculation shows that K_x_(NH_4_)_y_V_4_O_10_ has modulated electronic structure and better diffusion path of Zn^2+^, and the migration barrier is lower than NH_4_V_4_O_10_. Based on electrochemical reaction kinetics analysis and ex situ characterizations, the possible charge storage mechanism is shown in Figure 24f.

#### 4.5.2 The Co‐Intercalation of Different Kinds of Cations and H_2_O

##### K_0.09_Mg_0.03_V_2_O_5_ *n*H_2_O

The intercalation of two ions (K^+^ and Mg^2+^) in hydrated vanadium oxides was investigated in terms of the contraction of the structure by K^+^ and the expansion of the structure by Mg^2+^.^[^ ^274^, ^275^, ^276^, ^371^ ^]^ Feng and colleagues^[^ ^372^ ^]^ intercalated both the monovalent metal K^+^ and the divalent alkaline metal Mg^2+^ into the V_2_O_5_ *n*H_2_O layers by a one‐step hydrothermal method. In addition, single K^+^ and single Mg^2+^ intercalations were also prepared, as shown in Figure 24g. Mg^2+^ can increase the spacing between V_2_O_5_ *n*H_2_O layers, expand ion transport channels, and improve the specific capacity of batteries. At the same time, K^+^ can make the connection between the V—O layers closer, so that the structure of the material is more stable. Because of the intercalation of these two ions, the KMgVOH cathode has an unprecedentedly high specific capacity of 423 mAh g^−1^ at 0.1 A g^−1^ (Figure 24h) and good cycle stability with a retention of 72% after 2000 cycles 4 A g^−1^ (Figure 24i). The storage mechanism of this process is shown in Figure 24j, which is proved by SEM, ex situ XRD, and XPS.

##### Na_0.3_(NH_4_)_0.6_V_4_O_10_ 0.4H_2_O

The Na^+^ pre‐intercalation strategy is promising to improve the cyclic life and electrochemical performance of NH_4_V_4_O_10_. Wang et al.^[^ ^373^ ^]^ prepared a non‐stoichiometric Na_0.3_(NH_4_)_0.6_V_4_O_10_ 0.4H_2_O nanorods as a cathode material for AZIBs. The combined effect of Na^+^ pre‐intercalated in NH_4_V_4_O_10_ and structural water enhances the diffusion kinetics, reduces the electrostatic repulsion of Zn^2+^ (de)intercalation, and keeps the layer structure stable. As shown in Figure 24k, XRD pattern is obtained to determine the crystal structure and phase purity of Na_0.3_(NH_4_)_0.6_V_4_O_10_ 0.4H_2_O. The pre‐intercalated Na^+^ replaces part of NH_4_^+^ located between VO layers and maintains the layer structure of NH_4_V_4_O_10_. The results show that the Na_0.3_(NH_4_)_0.6_V_4_O_10_ 0.4H_2_O cathode has an excellent specific capacity of 400.2 mAh g^−1^ at 0.1 A g^−1^, and as shown in Figure 24l, its capacity retention rate reaches 97.2% after 2000 cycles at 10 A g^−1^. In addition, the reversible intercalation mechanism of Zn^2+^ in Na_0.3_(NH_4_)_0.6_V_4_O_10_ 0.4H_2_O is investigated by means of ex situ XRD (Figure 24m) and XPS analysis (Figure 24n). On this basis, as shown in Figure 24o, the electrochemical intercalation behavior of Zn^2+^ in Na_0.3_(NH_4_)_0.6_V_4_O_10_ 0.4H_2_O during (dis)charging is graphically summarized.

##### P(Phosphating)‐Na_1.04_Co_0.54_V_8_O_20_ 1.1H_2_O

Phosphating process is also an important method to improve the electrochemical properties of vanadium‐based compounds, triggering the local strain of vanadium oxide layer structure, resulting in the local increase of lattice spacing.^[^ ^374^ ^]^ Du and coworkers^[^ ^375^ ^]^ addressed the challenges of simultaneously avoiding irreversible phase formation and stabilizing host structures during cycling by introducing oxygen vacancies and surface phosphate groups in Na^+^ and Co^2+^ co‐intercalated V_8_O_20_ nanobelts. The introduced oxygen defects and phosphate groups promote charge transfer and increase the electronic conductivity of the cathode. Therefore, the prepared cathode has a high capacity of 161.8 mA h g^−1^ at 10 A g^−1^, a long cycle life of 96.8% capacity retention after 3000 cycles (Figure 24p), and an excellent rate performance of 124.3 mA h g^−1^ at 20 A g^−1^. To clearly illustrate the modulation of the electronic structure, the electron density of P‐Na_1.04_Co_0.54_V_8_O_20_ 1.1H_2_O is shown in Figure 24q. Du et al.^[^ ^375^ ^]^ obtained the crystal structure evolution diagram based on a variety of characterization methods, as shown in Figure 24r and **Table** **3**.

**Table 3:** Electrochemical performance of vanadates as cathodes in AZIBs

Materials	Electrolyte	Specific capacity [mAh g^−1^] (current density [A g^−1^])	Capacity retention (cycles numbers)	Voltage range [V]	Ref.
LiV_3_O_8_	1 m ZnSO_4_	256 (0.016)	75.0% (65)	0.6–1.2	[241]
Li_x_V_2_O_5_ *n*H_2_O	2 m ZnSO_4_	407.6 (1)	76.3% (500)	0.4–1.4	[279]
Na_0.33_V_2_O_5_	3 m Zn(CF_3_SO_3_)_2_	367 (0.1)	93.0% (1000)	0.2–1.6	[262]
Na_0.56_V_2_O_5_	3 m ZnSO_4_+ 0.5 m Na_2_SO_4_	317 (0.1)	84.0% (1000)	0.4–1.5	[376]
Na_1.25_V_3_O_8_	3 m Zn(CF_3_SO_3_)_2_	390 (0.1)	88.2% (2000)	0.2–1.9	[377]
Na_2_V_6_O_16_ 3H_2_O	1 m ZnSO_4_	361 (0.1)	80.0% (1000)	0.4–1.4	[333]
Na_2_V_6_O_16_⋅2.14H_2_O	1 m ZnSO_4_⋅7H2O	466 (0.1)	90.0% (2000)	0.2–1.6	[378]
K_0.23_V_2_O_5_	2 m Zn(CF_3_SO_3_)_2_	284 (0.1)	92.8% (500)	0.1–1.7	[266]
K_0.25_V_2_O_5_	2 m ZnSO_4_	306 (0.1)	99.0% (500)	0.4–1.4	[379]
KV_2_O_5_	1 m ZnSO_4_	439 (0.05)	96.0% (1500)	0.4–1.4	[380]
Na_1.2_V_3_O_8_/K_2_V_6_O_16_ 1.5H_2_O	3 m ZnSO_4_	393 (0.1)	85.0% (800)	0.4–1.4	[381]
MgV_2_O_4_	2 m Zn(TFSI)_2_	272 (0.2)	60.6% (500)	0.2–1.4	[382]
Mg_x_V_2_O_5_ *n*H_2_O	3 m Zn(CF_3_SO_3_)_2_	353 (0.1)	97.0% (2000)	0.1–1.8	[276]
Ca_0.67_V_8_O_20_ 3.5H_2_O	3 m Zn(CF_3_SO_3_)_2_	466 (0.1)	74.0% (2000)	0.4–1.5	[383]
Ag_0.4_V_2_O_5_	3 m ZnSO_4_	237 (0.5)	93.0% (4000)	0.4–1.4	[384]
Co_0.247_V_2_O_5_ 0944H_2_O	3 m Zn(CF_3_SO_3_)_2_	432 (0.1)	90.3% (7500)	0.3–2.2	[385]
*δ*‐Ni_0.25_V_2_O_5_ *n*H_2_O	3 m ZnSO_4_	402 (0.2)	98.0% (1200)	0.3–1.7	[386]
CuV_2_O_6_	3 m Zn(CF_3_SO_3_)_2_	427 (0.1)	99.3% (3000)	0.2–1.6	[387]
CuV_2_O_6_	3 m Zn(CF_3_SO_3_)_2_	338 (0.1)	100.0% (1200)	0.2–1.4	[388]
Cu_3_(OH)_2_V_2_O_7_ 2H_2_O	3 m Zn(CF_3_SO_3_)_2_	336 (0.1)	80.0% (3000)	0.4–1.4	[389]
Cu_3_V_2_O_7_(OH)_2_ 2H_2_O	2.5 m Zn(CF_3_SO_3_)_2_	216 (0.1)	89.3% (500)	0.2–1.6	[390]
PEDOT‐NH_4_V_3_O_8_	3 m Zn(CF_3_SO_3_)_2_	357 (0.05)	94.1% (5000)	0.4–1.6	[391]
(NH_4_)_2_V_3_O_8_	3 m Zn(CF_3_SO_3_)_2_	356 (0.1)	50.7% (2000)	0.4–1.6	[339]
(NH_4_)_2_V_4_O_9_	3 m Zn(CF_3_SO_3_)_2_	376 (0.1)	87.6% (2000)	0.3–1.3	[392]
NH_4_V_4_O_10_	3 m Zn(CF_3_SO_3_)_2_	147 (0.2)	70.3% (5000)	0.8–1.7	[362]
(NH_4_)_2_V_6_O_16_	3 m Zn(CF_3_SO_3_)_2_	324 (0.1)	78.3% (2000)	0.3–1.7	[393]
(NH_4_)_2_V_6_O_16_ 1.5H_2_O	2 m ZnSO_4_	479 (0.1)	76.0% (3000)	0.2–1.6	[356]
(NH_4_)_2_V_6_O_16_ 1.5H_2_O	3 m Zn(CF_3_SO_3_)_2_	120 (0.1)	75.0% (10 000)	0.4–1.6	[357]
(NH_4_)_2_V_10_O_25_ 8H_2_O	3 m Zn(CF_3_SO_3_)_2_	417 (0.1)	63.6% (500)	0.3–1.3	[364]
(NH_4_)_2_V_10_O_25_ 8H_2_O	3 m Zn(CF_3_SO_3_)_2_	229 (0.1)	90.1% (5000)	0.7–1.7	[334]
NaCa_0.6_V_6_O_16_ 3H_2_O	3 m Zn(CF_3_SO_3_)_2_	347 (0.1)	94.0% (2000)	0.4–1.5	[394]

## 5 Other Vanadium‑Based Compounds

### 5.1 Vanadium Sulfides

**Figure 25:**
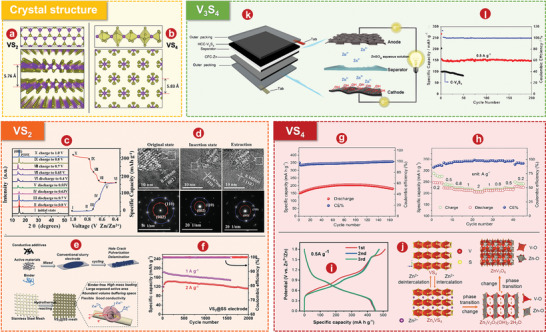
Schematic showing the geometries of a) VS_2_ and b) VS_4_. (a) Top‐view image (top) and side‐view image (bottom) showing 2D sheets of VS_2_. (b) Repeating unit of the 1D chain structure of VS_4_ (top) and side‐view image of monoclinic VS_4_ optimized using DFT. The purple balls are V atoms, and the yellow‐green balls are S atoms. c) Ex situ XRD patterns of VS_2_ collected at various states. d) HR‐TEM images and SAED patterns. e) Schematic illustration of the preparation processes for (top) the conventional slurry‐coated electrode, and (bottom) the binder‐free hierarchical VS_2_@SS electrode. f) Long‐term cycling performance of the VS_2_@SS electrode at 1 and 2 A g^−1^. g) Cycle performance of VS_4_@rGO at 1 A g^−1^. h) Rate performance of VS_4_@rGO. i) Discharge‐charge curves of VS_4_@rGO composite electrode at 0.5 A g^−1^. j) Schematic illustration showing the structural evolution of VS_4_ during discharging/charging processes. k) Schematic of the HCC‐V_3_S_4_//CFC–Zn flexible device construction and microscopic components. l) Comparison of the cycling stability of HCC‐V_3_S_4_//CFC–Zn and C‐V_3_S_4_//CFC–Zn at 0.5 A g^−1^. a, b) Reproduced with permission.^[^ ^398^ ^]^ Copyright 2013, American Chemical Society. c, d) Reproduced with permission.^[^ ^149^ ^]^ Copyright 2017, WILEY‐VCH. e, f) Reproduced with permission.^[^ ^401^ ^]^ Copyright 2019, The Royal Society of Chemistry. g, h) Reproduced with permission.^[^ ^407^ ^]^ Copyright 2018, The Royal Society of Chemistry. j) Reproduced with permission.^[^ ^408^ ^]^ Copyright 2021, The Royal Society of Chemistry. k, l) Reproduced with permission.^[^ ^411^ ^]^ Copyright 2019, American Chemical Society.

**Figure 26:**
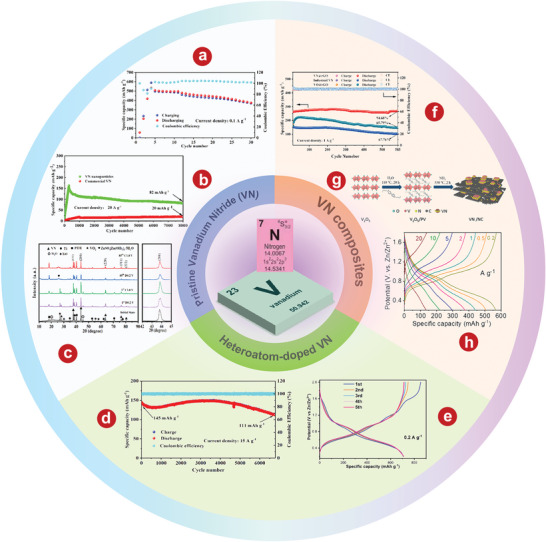
a) Cyclic curve of VN particles at a 0.1 A g^−1^. b) Cycle stability of VN particles and commercial VN under 20 A g^−1^. c) Ex situ XRD patterns of VN nanoparticles at a discharge voltage of 0.2 V and a charge voltage of 1.8 V for the first and 85th cycles. d) Cyclic stability of coral VN/C under 15 A g^−1^. e) Charge‐discharge profiles of O‐VN‐based AZIBs. f) Cycle performance of VN@rGO, industrial VN, and VO@rGO at 1 A g^−1^. g) Schematic of the synthetic procedure for the VN/NC hybrid nanosheets. h) Galvanostatic charge‐discharge plots of Zn//VN/NC cells at different current densities. a–c) Reproduced with permission.^[^ ^417^ ^]^ Copyright 2021, American Chemical Society. d) Reproduced with permission.^[^ ^418^ ^]^ Copyright 2021, American Chemical Society. e) Reproduced with permission.^[^ ^77^ ^]^ Copyright 2021, Elsevier. f) Reproduced with permission.^[^ ^420^ ^]^ Copyright 2021, Elsevier. g, h) Reproduced with permission.^[^ ^421^ ^]^ Copyright 2022, The Royal Society of Chemistry.

Vanadium sulfides mainly include VS_2_ and VS_4_, as well as VS, VS_6_, V_2_S_3_, V_2_S_5_, V_3_S phases. Among them, VS_2_ and VS_4_ are typical, which have attracted much attention in recent years.^[^ ^395^ ^]^ The VS_2_ crystal has a layered structure with an interlayer spacing of 5.76 Å and consists of a hexagonal‐filled metal vanadium layer sandwiched between two layers of sulfur atoms (**Figure** **25**a).^[^ ^396^ ^]^ While VS_4_ crystal is a quasi 1D chain compound consisting of V^4+^ coordinated with S_2_^2−^ dimers (**Figure** **26**b), and the linear structural units are stacked together by weak van der Waals interactions with an interchain distance of 5.83 Å.^[^ ^397^ ^]^ The oxidation states of vanadium in VS_2_ and VS_4_ are the same, but the oxidation states of sulfide are different (There is an S_2_^−^monomer in VS_2_ and an S_2_^2−^ dimers in VS_4_).^[^ ^398^ ^]^

#### 5.1.1 VS_2_

In the crystal structure of VS_2_, each V atom is arranged around six S atoms and is covalently linked to the S atoms. The widely spaced layers of VS_2_ allow easy (de)intercalation of Li^+^, Na^+^, Zn^2+^, or their solvation sheath in electrolyte.^[^ ^399^, ^400^ ^]^ The VS_2_ nanosheets were synthesized by He et al.^[^ ^149^ ^]^ though a simple hydrothermal reaction, and had a high capacity of 190.3 mA h g^−1^ at 0.05 A g^−1^, as well as stable cycling stability as cathode materials of AZIBs. Ex situ TEM, ex situ XRD, and selected area electron diffraction (SAED) pattern results (Figure 25c,d) show that the interlayer space of VS_2_ self‐adapting the Zn^2+^ intercalation expands along *c*‐axis only 1.73% and slightly shrinking on the *a‐* and *b‐*axes, which plays an important role in the realization of AZIBs with long life. Jiao and coworkers^[^ ^401^ ^]^ developed an independent, free‐binding cathode for AZIBs, consisting of hierarchical VS_2_ in the 1T phase grown directly on a SS mesh (Figure 25e). The design of the open‐structure electrode is beneficial to increase the contact area with the electrolyte, minimize the transmission path of zinc ions and electrons, reduce volume expansion, and achieve stable circulation. Therefore, the battery has a great zinc ion storage capacity of 198 mAh g^−1^ and long‐time cycle performance with a capacity retention rate of more than 80% at 2 A g^−1^ after 2000 cycles (Figure 25f).

#### 5.1.2 VS_4_

As an analog of VS_2_, the VS_4_ has a unique chain structure and a S_2_^2−^ group component, and is used for the research of energy storage materials.^[^ ^402^, ^403^, ^404^, ^405^, ^406^ ^]^ Qian et al.^[^ ^407^ ^]^ developed a patronite form of vanadium sulfide anchored on rGO prepared by a simple hydrothermal method and can be used as a cathode with high performance for AZIBs. As shown in Figure 25g, VS_4_@rGO cathode exhibits an excellent capacity of 180 mAh g^−1^ with a capacity retention of 93.3% after 165 cycles at 1 A g^−1^ thanks to VS_4_ unique crystal structure and rGO superior electrical conductivity. At the same time, when the current density increased from 0.2 to 2 A g^−1^, the capacity retention rate can reach 83.7% (Figure 25h). On the basis of this work, Chen et al.^[^ ^408^ ^]^ designed morphologically optimized VS_4_@rGO composites with ultra‐high specific capacity of 450 mA h g^−1^ at 0.5 A g^−1^ (Figure 25i) and high rate capacity of 313.8 mA h g^−1^ at 10 A g^−1^, when used as AZIBs cathode materials. In addition, as shown in Figure 25j, an irreversible phase transition of VS_4_ to Zn_3_(OH)_2_V_2_O_7_ 2H_2_O during charging and further from Zn_3_(OH)_2_V_2_O_7_ 2H_2_O to ZnV_3_O_8_ was found during long‐term cycling, which may be the main reason for the VS_4_@rGO capacity decline.

#### 5.1.3 V_3_S_4_

The crystal structure of V_3_S_4_ has ordered V vacancies, which constitute the superstructure of the NiAs‐type structure. This structure can also be described as VS_2_ monolayer building blocks alternating between additional V atoms. Thus, an increase in electronic/ionic conductivity is expected.^[^ ^409^, ^410^ ^]^ Liu and colleagues^[^ ^411^ ^]^ first proposed a new hydrophilic carbon substrate with acidic treated natural halloysite and CNTs as structural and interface modifiers, loading V_3_S_4_ as a composite cathode (HCC‐ V_3_S_4_) into a flexible AZIB (Figure 25k). This flexible AZIB has a high specific capacity of 148 mAh g^−1^ with a capacity retention of 95% after 200 cycles at 0.5 A g^−1^ (Figure 25l), an excellent rate performance, a high energy density of 155.7 W h kg^−1^, as well as a high power density of 5000 W kg^−1^.

### 5.2 Vanadium Nitrides

The capacity degradation and kinetics retardation of Zn^2+^ exist in the cycling process.^[^ ^316^, ^412^ ^]^ Recently, VNs with cubic structures have become new cathode materials for AZIB to solve these problems.^[^ ^413^, ^414^ ^]^ It has been reported that VN‐based materials undergo high‐potential inverse reactions during initial charging and exhibit high capacity from the second cycle onwards.^[^ ^415^, ^416^ ^]^ VNs generally come in three forms: VN, V_2_N, and V_3_N. Among them, VN is an isomer of VC and VO and belongs to the face‐centered cubic structure, which is most widely used in AZIBs due to its good conductivity and spatial structure.

#### 5.2.1 VN

Rong and coworkers^[^ ^417^ ^]^ synthesized highly stable VN particles by reduction and nitrification of V_2_O_5_ in an NH_3_ atmosphere. Thanks to their tiny particle size and porous stacking structure, after 10 cycles of activation at a voltage range of 0.2–1.7 V, VN particles have a specific capacity of 496 mAh g^−1^ at 1 A g^−1^ (Figure 26a). Even at 20 A g^−1^, the capacity of VN particles is 153 mA h g^−1^, and after activation remains 82 mA h g^−1^ after 8000 cycles (Figure 26b). In order to reveal the activation mechanism of VN particles, the XRD patterns of the 1st and 85th cycles are studied at a discharge voltage of 0.2 V and a charge voltage of 1.8 V (Figure 26c). Heteroatom doping can significantly improve the low conductivity of vanadium‐based compounds and increase the transport rate of Zn^2+^ in electrolytes. Su et al.^[^ ^418^ ^]^ prepared coral carbon‐doped VN by one‐step solvothermal method and ammonia nitration roasting. Thanks to the nanoscale size and porous stacked structure, the coral carbon‐doped VN cathode material has a specific capacity of 322 mAh g^−1^ at 0.5 A g^−1^. When assembling the coral carbon‐doped VN cathode in AZIB, this AZIB delivers a specific capacity of 111 mAh g^−1^ with 6780 cycles and 95% capacity retention at 15 A g^−1^ (Figure 26d). In addition, Chen et al.^[^ ^77^ ^]^ developed an oxygen‐doped VN (O‐VN) cathode and for the first time confirmed the highly reversible cation conversion reaction of O‐VN cathode in AZIBs. As shown in Figure 26e, the O‐doped VN cathode shows an ultra‐high discharge capacity of 705 mAh g^−1^ at 0.2 A g^−1^ due to cation conversion reactions and Zn^2+^ deintercalation. The electrical conductivity of VN can be significantly improved by combining VN with conductive carbon composites.^[^ ^419^ ^]^ Chen and colleagues^[^ ^420^ ^]^ modified VN for industrial use with the high conductivity of rGO, giving the VN@rGO electrode high‐rate capability and long period stability. The specific capacity of VN@rGO is 267.0 mA h g^−1^, and the specific capacity retention rate is 94.68% at 1 A g^−1^ after 585 cycles, better than that of VN and VO@rGO (Figure 26f). Chen et al.^[^ ^419^ ^]^ also demonstrated through kinetic studies that rGO can accelerate the redox reactions on the electrode surface to improve the pseudo‐capacitance of the electrode by accelerating electron transport. In addition, as shown in Figure 26g, Niu et al.^[^ ^421^ ^]^ developed new layer‐by‐layer VN/N‐doped carbon hybrid nanosheets (VN/NC) as cathode materials through in situ thermal conversion of pyrolyzing pentyl viologen intercalated V_2_O_5_. At 0.2 A g^−1^, the VN/NC cathode shows a high discharge specific capacity of 566 mAh g^−1^ (Figure 26h) and superior rate capability. Moreover, after 1000 cycles at 10 A g^−1^, VN/NC cathode has a good cycle stability of 131 mAh g^−1^ with a capacity retention rate of 85% after more than 1000 cycles.

## 6 Summary and Outlook

To date, vanadium‐based compounds reported for use as AZIBs cathodes exhibit a variety of crystal structures and properties, including typical layered vanadium‐based compounds with high Zn^2+^ storage capacity, tunnel‐typed vanadium‐based compounds with high power density, and NASICON‐typed materials with stable frames and ideal thermal stability. In this review, the preparation methods, structural characteristics, electrochemical performance, energy storage mechanism, and various effective ways to improve the electrochemical performance of vanadium‐based compounds are reviewed, including vanadium phosphates, vanadium oxides, vanadates, vanadium sulfides, VNs. The main challenges can be summarized as follows:

1) Although Li^+^ (0.74 Å) and Zn^2+^ (0.76 Å) have similar ionic radii, however, the electrostatic interaction between divalent Zn^2+^ and cathode material framework is much stronger than that of Li^+^, and the larger zinc hydrate compounds is difficult to co‐intercalation. Therefore, Zn^2+^ diffuses slowly into the solid state of the cathode lattice.
2) Most layered vanadium‐based compounds are composed of VO_x_ layers with weak van der Waals interactions, which are prone to irreversible phase transitions and structural collapse during repeated Zn^2+^ (de)intercalation, thus limiting cyclic stability.
3) Most vanadium‐based compounds are soluble in acidic electrolyte solutions. Therefore, the dissociation and intercalation of H^+^ are likely to lead to framework collapse and capacity decay during repeated cycles. Therefore, vanadium‐based compounds have considerable structural instability, resulting in loss of active materials due to vanadium dissolution.

In order to overcome the above problems and realize the practical and large‐scale applications of vanadium‐based compounds in AZIBs, the following future research directions can be proposed:

1) The exploration of zinc storage mechanism is of great significance for the basic understanding of advanced AZIBs systems and their large‐scale applications in the future. There are three kinds of conventional Zn^2+^ (de)intercalation mechanisms based on vanadium‐based compounds (Zn^2+^ intercalation mechanism, H^+^/Zn^2+^ co‐intercalation mechanism, dual metal ion co‐intercalation mechanism). However, it is still difficult to explain the zinc storage mechanism of vanadium‐based compounds because of the lack of a reliable theoretical basis and advanced characterization techniques. Therefore, the development of more accurate characterization techniques combined with ab initio calculations will contribute to a better understanding of zinc storage mechanisms and the relationship between structure and properties, providing a good guide for understanding and designing more efficient AZIBs cathode materials.
2) The importance of electrolytes cannot be ignored. The solution of vanadium can be solved with a suitable electrolyte such as “water in salt” or Zn(CF_3_SO_3_)_2_. It is proved that the appropriate composition ratio in the multicomponent salt electrolyte can promote the (de)intercalation of Zn^2+^ at the cathode‐electrolyte interface by inhibiting water activity. The flexible choice of electrolyte types (e.g., gel, solid) offers the possibility for functionalized applications. However, how to balance interface stability, inhibition of side reactions with appropriate mobility, and energy storage media activity is the primary consideration in electrolyte design.
3) Hundreds of vanadium‐based compounds with various tunnel or layer spacing structures have been discovered, and they are highly likely to be the preferred candidates for AZIBs. Therefore, it is an important research direction to study the synthesis route and zinc storage mechanism of suitable vanadium‐based compounds. In addition, the pre‐intercalation of cation, H_2_O molecules, and conductive organic polymers can reduce the strong electrostatic interaction between the V—O layer and the highly polarized Zn^2+^, and then reduce the migration energy barrier of Zn^2+^. Pre‐intercalation is also an effective strategy to strengthen the layered structure of vanadium‐based oxides, expand the interlayer spacing and avoid structural collapse. The cations (or/and H_2_O molecules) pre‐intercalate between the V—O layers and act as pillars to chemically strengthen the layers, improve structural stability and inhibit destructive structural changes. In addition to having the above functions, the pre‐intercalation of conductive organic polymers can also be used as guest storage for Zn^2+^. In order to enrich the members of the vanadium‐based material family, more types of vanadium‐based compounds containing the pre‐intercalation of cations or/and conducting organic polymers should be developed.
4) In addition to the pre‐intercalation strategy, there are also two attractive ways to stabilize the host structure and enhance the electrical conductivity, which is called surface composite and heteroatoms doping. In addition, defect engineering, such as the creation of oxygen vacancies, can further enhance the Zn^2+^ storage behavior of vanadium‐based compounds for AZIBs by modulating the diffusion properties of electronic and ion diffusion by adsorbing Zn^2+^ on the surface of materials. Therefore, further development of surface engineering, heteroatom doping, and defect engineering are effective strategies to enhance the electronic conductivity of vanadium‐based compounds and promote the migration of ions and electrons in cathode.
5) It is also an important approach to develop vanadium‐based compounds with different morphology, including 1D, 2D nanostructures, and 3D nano/micro‐structures, hollow/porous structures. The nanostructures with 1D micron dimensions can facilitate current collection. 2D nanostructures not only have the advantages of 1D nanostructures, but also are more conducive to ions or electrons transport due to their ultra‐thin thickness. In addition, the highly exposed surface of 2D nanostructures can shorten the migration path of ions and provide more active sites for redox reactions. In summary, the nanostructure can inhibit volume change through local blank, thus achieving high structural stability and improving reversible capacity. However, reducing side reactions between cathode and electrolyte to achieve high cyclic stability remains a major challenge. 3D nano/micro‐structures composed of nanostructures not only have the advantages of nanomaterials, but also have higher bulk density. In addition, hollow/porous structures generally offer more possibilities for improving electrochemical performance by buffering volume expansion, providing more active sites, and facilitating electrolyte penetration. Therefore, the construction of 3D hollow/porous nano/micro‐structures may be an effective strategy to improve electrochemical performance, as this unique morphology can inhibit the agglomeration of nanostructures and regulate the volume changes during cycling. Therefore, precise structural designs with high surface area and abundant porosity can enhance electrochemical performance.

In addition to the above‐existing problems and future prospects, the authors are suggested to pay more attention to the following problems in the process of experimental exploration:

1) A stable frame during cycling is a priority in selecting suitable vanadium‐based compounds. Reversible changes in layer spacing have been observed in most studies, with water molecules or trapped cations playing a key role in stabilizing the crystal structure. The relationship between the electrolyte type and concentration, the solvation effect of Zn^2+^, and the crystal structure of the vanadium‐based compounds is interrelated and therefore requires further investigation. Changing any one of these three factors can lead to a different reaction process, which in turn affects the performance of AZIBs.
2) At high current densities, the GCD curves and CV responses in the first period sometimes differ from those in later periods. This may be related to the good self‐regulation of the crystal structure in the first cycle to serve the rapid insertion/removal of zinc ions. This autoregulatory process may be related to the changes in V—O polyhedra and their connection types. A deeper understanding of this process is important to improve productivity and cycle performance.

In general, AZIBs have the advantages of safety, environmental protection, low toxicity, simple manufacturing, and so on. Compared with other battery systems, AZIBs have become one of the most promising battery systems in recent years. Although it may be too early to commercialize, the development of high‐performance cathode materials could accelerate their commercialization process. Various vanadium‐based compounds with low cost, high theoretical capacity, and high power density have been widely used as cathodes for AZIBs. In this review, the advantages and disadvantages of vanadium‐based compounds are analyzed systematically as cathode materials, and the prospects of further development of vanadium‐based compounds and AZIBs are put forward. With the continuous innovation of advanced characterization techniques and the discovery of new materials, the future commercialization challenges of low‐cost AZIBs will be overcome one by one.

## Conflict of Interest

The authors declare no conflict of interest.
